# Chronic CBD treatment differentially modulates neurobehavioral outcomes and endocannabinoid signaling in an aged HIV-1 Tat transgenic mouse model

**DOI:** 10.1371/journal.pone.0353267

**Published:** 2026-07-20

**Authors:** Barkha J. Yadav-Samudrala, Morgan L. Johnson, Ashima Ale, Katherine Xiaofan Jiang, William W. Y. Lee, Amy Nguyen, Dorian Ho, Essie B. Acquah, Isabella C. Orsucci, Laith E. Sawaqed, Gabriella Boyer, Justin L. Poklis, Wei Jiang, Sylvia Fitting

**Affiliations:** 1 Department of Psychology and Neuroscience, University of North Carolina at Chapel Hill, Chapel Hill, North Carolina, United States of America; 2 Department of Pharmacology and Toxicology, Virginia Commonwealth University, Richmond, Virginia, United States of America; 3 Department of Microbiology and Immunology, Medical University of South Carolina, Charleston, South Carolina, United States of America; 4 Division of Infectious Diseases, Department of Medicine, Medical University of South Carolina, Charleston, South Carolina, United States of America; Nathan S Kline Institute for Psychiatric Research, UNITED STATES OF AMERICA

## Abstract

As the population of older individuals living with HIV continues to expand, identifying non-euphoric therapeutic interventions for HIV-associated complications is essential. While cannabidiol (CBD) has demonstrated neuroprotective potential, its effects following prolonged exposure in the context of an aging biological environment remain poorly understood. This study utilized aged (15–18 months) male and female HIV-1 Tat transgenic mice to evaluate the impact of chronic CBD (3 mg/kg, s.c., for 12 weeks). A behavioral battery was employed to assess object recognition memory, anxiety-like behavior, spontaneous nociception, and locomotor activity. Subsequently, liquid chromatography-tandem mass spectrometry and Western blotting were used to map endocannabinoid ligands (AEA, 2-AG, AA), metabolic enzymes (FAAH, MAGL), and receptors (CB_1_R, CB_2_R, GPR55) across the prefrontal cortex, amygdala, brainstem, and spinal cord. Results indicated that chronic CBD improved recognition memory specifically in female Tat(+) mice. While CBD treatment did not affect spinal cord-related tail flick sensitivity it universally increased supraspinal hot plate sensitivity. Chronic CBD treatment also elevated baseline body temperature and modulated body mass without impairing general locomotion. Furthermore, chronic CBD restructured the eCB signaling landscape across all examined CNS regions in a highly sex- and genotype-dependent manner. Notably, cannabinoid receptor and GPR55 expression exhibited distinct regulatory shifts governed by significant three-way interactions. These findings demonstrate that chronic CBD intervention interacts with the eCB system of aged mice in a region-specific manner. These results underscore the critical importance of considering age, sex, and treatment duration when evaluating cannabinoid-based therapies for the management of neuroHIV.

## Introduction

Cannabidiol (CBD) is one of the major constituents of Cannabis [1]. CBD is considered a psychoactive compound as it affects behavior [2–6]. However, it is considered a non-euphoric compound, unlike Δ^9^-tetrahydrocannabinol (THC), the second major constituent of Cannabis, as it does not possess rewarding and addictive properties [7–9]. Recent policy changes have facilitated access to cannabis-based therapies globally. With the approval of United States Agriculture Improvement Act of 2018, also known as the “Farm Bill,” hemp and its derivatives were removed from the U.S. Drug Enforcement Administration’s controlled substances list [10]. As of 2024, 38 states and the District of Columbia have legalized cannabis for medical use, and 24 states permit nonmedical use, making the cannabis market a $40 billion industry in the United States alone [11]. These legislative advancements have driven rapid expansion for cannabinoid-based products in retail market.

The exact molecular pathway and mechanism through which CBD acts have not been fully elucidated yet [12]; however, studies have suggested that CBD directly interacts with numerous receptors [13], engaging multiple pathways that contribute to different therapeutic applications [14,15]. With regards to the endocannabinoid (eCB) system, CBD has low binding affinity for cannabinoid type 1 and 2 receptors (CB_1_R and CB_2_R, respectively) [16] and therefore modulates CB_1_R and CB_2_R-related signaling via indirect pathways [17]. CBD also inhibits the enzymatic activity of fatty acid amide hydrolase (FAAH), the enzyme responsible for degrading the endogenous ligand *N*-arachidonoylethanolamine (AEA) [17]. As AEA functions as an agonist of cannabinoid receptors, the CBD-mediated increase in AEA levels may indirectly enhance CB_1_R and CB_2_R signaling [17]. Conversely, CBD may also reduce the binding affinity of CB_1_R agonists, thereby acting as a negative allosteric modulator [18]. CBD also interacts with a range of non-cannabinoid receptors such as transient receptor potential vanilloid (TRPV) channels [19], serotonin (5-HT1A) receptors [20,21], peroxisome proliferator-activated receptor gamma (PPARγ) [22–24], and the cannabinoid-related receptor G protein-coupled receptor 55 (GPR55) [25].

CBD has emerged as a compound with significant therapeutic potential, exhibiting antioxidant, anti-inflammatory, and neuroprotective properties [26–28]. A growing body of research supports its effectiveness in alleviating symptoms associated with neurological and neurodegenerative disorders, including chronic pain [29–32], epilepsy [33,34], Alzheimer’s disease [35–38], Parkinson’s disease [39–41], anxiety and stress [42–44], depression [45–48], and substance use disorders [28,49,50]. Moreover, recent findings from our laboratory have further highlighted the therapeutic benefits of CBD, specifically in the context of HIV-associated neurocognitive disorders (HAND). It was found that acute CBD treatment in the HIV-1 transactivator of transcription (Tat) transgenic mouse model differentially modulated the eCB system and related lipids in male and female mice, suggesting a sex-specific influence on neurobiological processes [51]. Additionally, acute CBD treatment altered locomotor activity and anxiety-like behavior, highlighting its potential impact on behavioral outcomes [51]. One particularly notable finding was the significant upregulation of the cannabinoid-related receptor GPR55 observed exclusively in female mice following acute CBD treatment [51]. This sex-specific receptor modulation provides further evidence of CBD’s diverse molecular targets and its ability to influence distinct signaling pathways.

Despite significant advancements in combined antiretroviral therapies (cART), HAND continues to affect approximately 30–50% of people living with HIV (PLWH) [52–54]. HAND encompasses a broad spectrum of central nervous system (CNS) symptoms, including deficits in attention, learning, and memory, impaired motor coordination [52,55,56], increased anxiety [57,58], and neuropathic pain [59]. These symptoms persist in the cART era due to chronic immune activation, neuroinflammation, and persistent low-level expression of viral proteins [60]. The widespread availability of effective cART has markedly increased the life expectancy of PLWH giving rise to a rapidly aging demographic that presents unique clinical challenges [61]. This population experiences a phenomenon of inflammaging, where persistent immune activation drives chronic systemic inflammation despite viral suppression [62,63]. Consequently, PLWH often exhibit accelerated aging, wherein neurocognitive decline progress more rapidly than in their HIV-negative counterparts [64].

One key contributor to HAND is the HIV-1 protein Tat [65], which remains elevated in the brains of PLWH exhibiting HAND symptoms [66]. Extensive *in vitro* and *in vivo* studies have established the neurotoxic properties of Tat, even in the absence of other HIV-1 viral proteins [67–69]. Tat is secreted by infected cells and exerts its effects both directly and indirectly within the CNS [70]. Directly, Tat damages neuronal structure and function [71,72], while indirectly, it activates glial cells, leading to increased proinflammatory signaling [73]. Studies employing HIV-1 Tat transgenic mouse models have demonstrated significant deficits in motor activity, learning and memory, motivation, anxiety, and HIV-related neuropathic pain, further underscoring Tat’s critical role in HAND pathophysiology [68,74–76]. Given the inflammatory nature of HAND, the eCB system has emerged as a promising therapeutic target for symptom management. The anti-inflammatory and neuroprotective properties of the eCB system make them particularly relevant for addressing HAND-associated neuropathology [77–81]. Clinical evidence indicates a severe disruption in eCB tone, marked by significantly lower levels of AEA [82] and OEA [83] potentially driven by enhanced FAAH-mediated degradation in addition to altered 2-AG dynamics [82]. Furthermore, post-mortem tissue from individuals with HIV encephalitis (HIVE) or HAND despite cART reveals a marked upregulation of CB_1_R and CB_2_R receptors in microglia, macrophages, and astrocytes [84,85], as well as a pathological shift in neuronal CB_1_R localization from a punctate distribution to the soma that correlates with cognitive decline [85]. This altered pathological landscape underscores the necessity of targeting the eCB system. Additionally, cannabis use is notably prevalent among PLWH, with usage rates estimated to be 2–3 times higher than in the general United States population, highlighting a potential interest in cannabinoid-based therapies [86,87].

**Fig 1:**
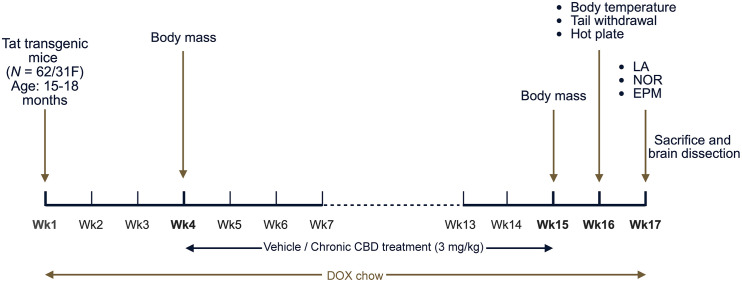
Experimental timeline for chronic CBD treatment and behavioral testing in HIV-1 Tat transgenic mice. To induce Tat expression, aging Tat transgenic mice (*N* = 62; 31f, 15–18 months old) were maintained on a doxycycline (DOX) diet for the duration of the study. Chronic CBD treatment (3 mg/kg) was administered from week 4 through week 15. Body mass was assessed longitudinally, with primary comparisons made between the acute phase (week 4) and chronic phase (week 15). At week 16, physiological and nociceptive measures were taken, including body temperature, tail withdrawal, and hot plate tests. Behavioral assays, consisting of locomotor activity (LA), novel object recognition (NOR), and the elevated plus maze (EPM), were conducted at week 17, followed by sacrifice and brain dissection for biochemical analysis (Tables 1 and 2).

Although CBD holds therapeutic promise as a non-euphoric cannabinoid, research examining its effects in the context of neuroHIV remains limited. To date, only a small number of *in vitro* and *in vivo* studies have explored the protective effects of CBD in neuroHIV [51,88,89], and none have examined the impact of prolonged CBD exposure alone. Furthermore, given that a significant and growing portion of the PLWH population is now over the age of 50, it is essential to evaluate therapeutic interventions in aging population. Therefore, in the present study we utilized an aged cohort of HIV-1 Tat transgenic mice (**Fig 1**) to investigate chronic (3 months treatment) effects of CBD (3 mg/kg) on various behavioral outcomes, including spontaneous nociception, motor activity, anxiety-like behavior, and object recognition memory. Further, chronic CBD effects on the eCB and related lipids were assessed by quantifying AEA, 2-arachidonoylglycerol (2-AG), *N*-palmitoylethanolamide (PEA), *N*-oleoylethanolamide (OEA), and their proinflammatory metabolite arachidonic acid (AA) in CNS regions, including the prefrontal cortex, striatum, hippocampus, amygdala, brainstem, and spinal cord. Lastly, protein levels of cannabinoid receptors (CB_1_R and CB_2_R), cannabinoid-like receptors (GPR55) and eCB degradative enzymes (FAAH and monoacylglycerol lipase, MAGL) were assessed in the prefrontal cortex, amygdala, brainstem, and spinal cord using western blot analysis (Tables 1 and 2).

**Table 1:** Tukey’s post hoc statistics for behavioral assessment following chronic CBD treatment.

Behavior	Sex	Genotype	Treatment		Interaction, *p* value	Tukey’s post hoc test*, p* value
			Vehicle mean ± SEM	CBD (3 mg/kg) mean ± SEM		
NOR: Total object exploration time (s)	Female	Tat(−)	222.86 ± 40.48	303.75 ± 44.59	**geno x treat,** ***p* = 0.01**	Tat(+) Veh > Tat(−) Veh, *p* = 0.04
		Tat(+)	272.50 ± 38.11	285.01 ± 38.11		
	Male	Tat(−)	177.50 ± 52.57	328.75 ± 36.81		
		Tat(+)	241.14 ± 51.50	241.14 ± 51.50		
NOR: Discrimination index (DI)	Female	Tat(−)	0.23 ± 0.09	0.68 ± 0.10	**sex x treat,** ***p* = 0.007**	Female CBD > Female Veh, *p* < 0.001 Female CBD > Male Veh, *p* = 0.006 Female CBD > Male CBD, *p* = 0.02
		Tat(+)	−0.07 ± 0.10	0.36 ± 0.14		
	Male	Tat(−)	0.29 ± 0.12	0.29 ± 0.06		
		Tat(+)	−0.009 ± 0.07	0.05 ± 0.06		

EPM: Total distance (m)	Female	Tat(−)	4.44 ± 0.73	3.88 ± 0.84	n.s.	n.s.
		Tat(+)	5.58 ± 1.02	5.35 ± 1.26		
	Male	Tat(−)	4.79 ± 1.11	6.27 ± 0.92		
		Tat(+)	8.06 ± 1.55	7.09 ± 1.71		
EPM: Average speed (m/s)	Female	Tat(−)	0.007 ± 0.00	0.006 ± 0.00	n.s.	n.s.
		Tat(+)	0.009 ± 0.00	0.008 ± 0.00		
	Male	Tat(−)	0.008 ± 0.00	0.010 ± 0.00		
		Tat(+)	0.013 ± 0.00	0.011 ± 0.00		
EPM: Open arm entries	Female	Tat(−)	1.86 ± 0.40	1.38 ± 0.62	n.s.	n.s.
		Tat(+)	6.13 ± 2.64	3.75 ± 0.75		
	Male	Tat(−)	2.50 ± 0.70	3.88 ± 0.29		
		Tat(+)	4.38 ± 1.23	5.00 ± 0.95		
EPM: open arm distance (m)	Female	Tat(−)	0.42 ± 0.17	0.22 ± 0.11	**sex x geno,** ***p* = 0.04**	n.s.
		Tat(+)	0.94 ± 0.49	0.40 ± 0.18		
	Male	Tat(−)	0.38 ± 0.17	0.83 ± 0.18		
		Tat(+)	0.33 ± 0.16	0.26 ± 0.07		

Tail withdrawal latency (s)	Female	Tat(−)	1.13 ± 0.03	1.02 ± 0.06	**sex x geno,** ***p* = 0.01**	Male Tat(+)> Female Tat(−), *p* < 0.001 Male Tat(+)> Female Tat(+), *p* < 0.001 Male Tat(+)> Male Tat(−), *p* = 0.001 = 2
		Tat(+)	1.14 ± 0.06	1.04 ± 0.10		
	Male	Tat(−)	1.15 ± 0.07	1.16 ± 0.11		
		Tat(+)	1.49 ± 0.16	1.58 ± 0.12		

Hot plate latency (s)	Female	Tat(−)	5.60 ± 0.68	5.00 ± 0.71	n.s.	n.s.
		Tat(+)	7.88 ± 1.18	5.07 ± 0.57		
	Male	Tat(−)	5.06 ± 0.58	4.80 ± 0.39		
		Tat(+)	7.31 ± 0.94	6.02 ± 0.63		

LA: Total activity	Female	Tat(−)	851.71 ± 45.76	832.5 ± 114.2	n.s.	n.s.
		Tat(+)	1081.38 ± 126.1	1049.25 ± 100.3		
	Male	Tat(−)	1199.00 ± 185.5	1109.75 ± 64.7		
		Tat(+)	1352.63 ± 119.2	1239.00 ± 153.2		
LA: Rearing episodes	Female	Tat(−)	69.43 ± 14.05	70.50 ± 14.90	n.s.	n.s.
		Tat(+)	83.00 ± 14.38	58.75 ± 14.89		
	Male	Tat(−)	74.62 ± 17.57	92.75 ± 12.38		
		Tat(+)	116.88 ± 16.73	101.86 ± 16.13		

CBD, cannabidiol; DI, discrimination index; EPM, elevated plus maze; geno, genotype; LA, locomotor activity; m, meter; m/s, meter/second; NOR, novel object recognition; n.s., not significant; s, second; Tat, transactivator of transcription; geno, genotype; treat, treatment; Veh, vehicle

**Table 2:** Tukey’s post hoc statistics for AEA, 2-AG and AA levels in prefrontal cortex, amygdala, brainstem, and spinal cord.

CNS region	Sex	Genotype	Treatment		Interaction, *p* value	Tukey’s post hoc test*, p* value
			Vehicle mean ± SEM	CBD (3 mg/kg) mean ± SEM		
PFC: AEA	Female	Tat(−)	0.26 ± 0.05	0.32 ± 0.06	n.s.	n.s.
		Tat(+)	0.21 ± 0.02	0.21 ± 0.03		
	Male	Tat(−)	0.17 ± 0.02	0.24 ± 0.02		
		Tat(+)	0.21 ± 0.01	0.25 ± 0.02		

PFC: 2-AG	Female	Tat(−)	2.29 ± 0.35	5.86 ± 1.16	**sex x geno x treat,** ***p* = 0.001**	Male Tat(−) Veh > Male Tat(+) Veh, *p* = 0.02 Male Tat(−) Veh > Male Tat(+) CBD, *p* = 0.02 Male Tat(−) Veh > Male Tat(−) CBD, *p* = 0.01 Male Tat(−) Veh > Female Tat(−) Veh, *p* = 0.03 Male Tat(−) Veh > Female Tat(+) CBD, *p* = 0.05
		Tat(+)	6.78 ± 2.22	2.79 ± 0.39		
	Male	Tat(−)	9.71 ± 3.45	1.63 ± 0.28		
		Tat(+)	2.21 ± 0.13	1.99 ± 0.20		

PFC: AA	Female	Tat(−)	454.8 ± 81.4	643.2 ± 105.6	**sex x treat,** ***p* = 0.03** **geno x treat, *p* = 0.01**	n.s. Tat(−) CBD > Tat(+) CBD, *p* = 0.04 Tat(+) Veh > Tat(+) CBD, *p* = 0.02
		Tat(+)	417.9 ± 33.2	356.1 ± 68.3		
	Male	Tat(−)	378.6 ± 50.8	442.2 ± 51.4		
		Tat(+)	691 ± 155.1	244.0 ± 24.6		

Amg: AEA	Female	Tat(−)	0.19 ± 0.04	0.15 ± 0.01	**sex x geno,** ***p* = 0.004** **sex x treat,** ***p* < 0.001** **sex x geno x treat,** ***p* = 0.04**	Male Tat(−)> Female Tat(−), *p* = 0.04 Female Veh > Female CBD, *p* = 0.002 Male CBD > Female CBD, *p* = 0.01 Female Tat(+) Veh > Female Tat(−) CBD, *p* = 0.001 Female Tat(+) Veh > Female Tat(+) CBD, *p* < 0.001 Female Tat(+) Veh > Male Tat(+) Veh, *p* = 0.01 Male Tat(−) CBD > Female Tat(+) CBD, *p* = 0.05
		Tat(+)	0.31 ± 0.02	0.14 ± 0.01		
	Male	Tat(−)	0.25 ± 0.01	0.26 ± 0.03		
		Tat(+)	0.17 ± 0.02	0.22 ± 0.03		

Amg: 2-AG	Female	Tat(−)	2.71 ± 0.35	6.13 ± 1.44	**geno x treat,** ***p* = 0.02**	Tat(+) Veh > Tat(−) Veh, *p* < 0.001 Tat(+) Veh > Tat(−) CBD, *p* = 0.04
		Tat(+)	11.47 ± 2.10	7.69 ± 2.12		
	Male	Tat(−)	3.05 ± 0.34	3.62 ± 0.82		
		Tat(+)	5.81 ± 0.79	4.83 ± 0.92		

Amg: AA	Female	Tat(−)	360.57 ± 48.1	368.28 ± 46.6	n.s.	n.s.
		Tat(+)	551.20 ± 22.6	369.37 ± 69.9		
	Male	Tat(−)	465.42 ± 60.2	550.42 ± 52.6		
		Tat(+)	734.00 ± 167.1	193.28 ± 10.9		

Spinal cord: AEA	Female	Tat(−)	0.006 ± 0.00	0.006 ± 0.00	**sex x geno,** ***p* = 0.009** **sex x treat,** ***p* = 0.01** **geno x treat,** ***p* = 0.02**	Female Tat(+)> Female Tat(−), *p* = 0.01 n.s. Tat(+) Veh > Tat(−), *p* = 0.03
		Tat(+)	0.026 ± 0.00	0.008 ± 0.00		
	Male	Tat(−)	0.013 ± 0.00	0.017 ± 0.00		
		Tat(+)	0.012 ± 0.00	0.015 ± 0.00		

Spinal cord: 2-AG	Female	Tat(−)	0.855 ± 0.13	5.66 ± 0.65	**sex x geno,** ***p* = 0.03** **sex x geno x treat,** ***p* < 0.001**	n.s. Female Tat(+) Veh > Female Tat(−) Veh, *p* < 0.001 Female Tat(+) Veh > Female Tat(−) CBD, *p* = 0.005 Female Tat(+) Veh > Female Tat(+) CBD, *p* < 0.001 Female Tat(+) Veh > Male Tat(−) CBD, *p* < 0.001 Female Tat(+) Veh > Male Tat(+) Veh, *p* < 0.001 Male Tat(−) Veh > Female Tat(−) Veh, *p* = 0.01 Male Tat(−) Veh > Female Tat(+) CBD, *p* = 0.01 Male Tat(−) Veh > Male Tat(−) CBD, *p* = 0.02 Male Tat(−) Veh > Male Tat(+) Veh, *p* = 0.02 Male Tat(+) CBD > Female Tat(−) Veh, *p* = 0.02 Male Tat(+) CBD > Female Tat(+) CBD, *p* = 0.02 Male Tat(+) CBD > Male Tat(+) Veh, *p* = 0.04
		Tat(+)	23.79 ± 5.51	1.27 ± 0.32		
	Male	Tat(−)	17.96 ± 5.02	2.70 ± 0.67		
		Tat(+)	2.26 ± 0.25	17.24 ± 5.51		

Spinal cord: AA	Female	Tat(−)	38.27 ± 3.30	65.01 ± 13.72	**sex x treat,** ***p* = 0.03**	n.s.
		Tat(+)	121.59 ± 16.21	60.23 ± 12.19		
	Male	Tat(−)	106.08 ± 8.86	183.22 ± 69.98		
		Tat(+)	122.93 ± 23.70	98.81 ± 28.78		

Brainstem: AEA	Female	Tat(−)	0.03 ± 0.00	0.01 ± 0.00	n.s.	n.s.
		Tat(+)	0.02 ± 0.00	0.02 ± 0.00		
	Male	Tat(−)	0.03 ± 0.00	0.04 ± 0.00		
		Tat(+)	0.03 ± 0.00	0.03 ± 0.00		

Brainstem: 2-AG	Female	Tat(−)	1.32 ± 0.26	19.17 ± 3.75	**sex x geno x treat,** ***p* < 0.001**	Female Tat(−) CBD > Female Tat(−) Veh, *p* < 0.001 Female Tat(−) CBD > Female Tat(+) CBD, *p* < 0.001 Female Tat(−) CBD > Male Tat(−) CBD, *p* < 0.001 Female Tat(−) CBD > Male Tat(+) Veh, *p* < 0.001 Male Tat(+) CBD > Female Tat(−) Veh, *p* = 0.006 Male Tat(+) CBD > Female Tat(+) CBD, *p* = 0.004 Male Tat(+) CBD > Male Tat(−) CBD, *p* = 0.008 Male Tat(+) CBD > Male Tat(+) Veh, *p* = 0.007 Female Tat(+) Veh > Female Tat(−) Veh, *p* = 0.03 Female Tat(+) Veh > Female Tat(+) CBD, *p* = 0.02 Female Tat(+) Veh > Male Tat(−) CBD, *p* = 0.04 Female Tat(+) Veh > Male Tat(+) Veh, *p* = 0.03 Male Tat(−) Veh > Female Tat(−) Veh, *p* = 0.04 Male Tat(−) Veh > Female Tat(+) CBD, *p* = 0.03
		Tat(+)	13.92 ± 2.64	1.19 ± 0.177		
	Male	Tat(−)	13.37 ± 3.42	2.23 ± 0.36		
		Tat(+)	2.04 ± 0.29	16.61 ± 4.96		

Brainstem: AA	Female	Tat(−)	133.30 ± 40.78	133.42 ± 28.29	**geno x treat,** ***p* = 0.001**	Tat(−) CBD > Tat(+) CBD, *p* = 0.03
		Tat(+)	162.77 ± 18.89	94.04 ± 16.28		
	Male	Tat(−)	115.26 ± 10.32	253.37 ± 56.50		
		Tat(+)	200.53 ± 30.41	113.75 ± 18.19		

2-AG, 2-arachidonoylglycerol; AA, arachidonic acid; AEA, *N*-arachidonoylethanolamine; Amg, amygdala; CBD, cannabidiol; CNS, central nervous system; geno, genotype; n.s., not significant; PFC, prefrontal cortex; Tat, transactivator of transcription; geno, genotype; treat, treatment; Veh, vehicle.

## Result

### Chronic CBD differentially attenuates Tat-induced cognitive impairments in females and alters the PFC endocannabinoid profile based on sex and Tat expression

**Fig 2:**
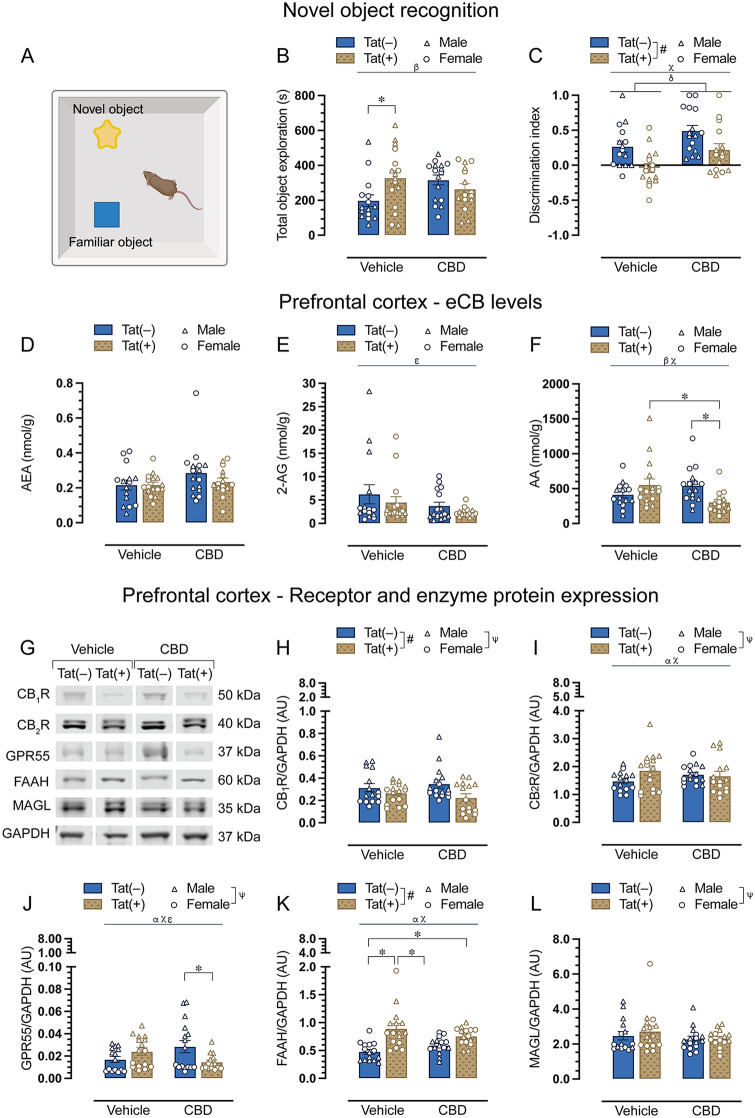
Chronic CBD treatment modulates prefrontal cortex-mediated memory and endocannabinoid signaling in a sex- and genotype-dependent manner. (A) Schematic representation of the novel object recognition (NOR) task. (B) Total object exploration time; chronic CBD treatment abolished the genotype-driven differences in exploration observed in vehicle treated mice. (C) Discrimination index (DI); chronic CBD treatment enhanced preference for the novel object for all mice, with the most pronounced effects observed in Tat(+) female mice. (D) AEA levels in the PFC; no significant differences were observed across groups. (E) 2-AG levels in the PFC; expression was modulated by a complex interaction between sex, genotype, and CBD treatment. (F) AA levels in the PFC; chronic CBD significantly reduced AA levels in Tat(+) mice compared to Tat(–) and vehicle-treated counterparts. (G) Representative Western blot bands for CB_1_R, CB_2_R, GPR55, FAAH, MAGL, and loading control GAPDH. (H) CB_1_R protein expression; levels were higher in males and Tat(–) mice regardless of treatment. (I) CB_2_R protein expression; levels were influenced by sex and a genotype x treatment interaction. (J) GPR55 protein expression; levels were sensitive to sex and genotype, with CBD inducing a genotype-specific shift in receptor density. (K) FAAH protein expression; CBD treatment abolished the Tat-induced elevation of FAAH observed in vehicle-treated mice. (L) MAGL protein expression; levels were significantly higher in males compared to females across all groups. Data represented as mean ± SEM. Statistical significance was assessed by overall ANOVAs, ^#^*p* < 0.05 main effect of genotype, ^ψ^*p* < 0.05 main effect of sex, ^δ^*p* < 0.05 main effect of treatment, ^α^*p* < 0.05 sex x genotype interaction, ^β^*p* < 0.05 sex x treatment interaction, ^χ^*p* < 0.05 genotype x treatment interaction, ^ε^*p* < 0.05 sex x genotype x treatment interaction. CBD dose = 3 mg/kg. *N* = 62(31f).

To evaluate the effects of chronic CBD treatment and Tat expression on prefrontal cortex (PFC) mediated memory, the novel object recognition (NOR) task was performed (Fig 2A). Cognitive performance was assessed using total object exploration time and the discrimination index (DI), where a DI of 1 represents a complete preference for the novel object, 0 represents no preference, and −1 represents a complete preference for the familiar object. To maintain clarity and conciseness, all significant Tukey’s post-hoc comparisons are detailed separately in Tables 1–3.

**Table 3:** Tukey’s post hoc statistics for CB_1_R, CB_2_R, GPR55, FAAH, and MAGL levels in prefrontal cortex, amygdala, brainstem, and spinal cord.

CNS region	Sex	Genotype	Treatment		Interaction, *p* value	Tukey’s post hoc test*, p* value
			Vehicle mean ± SEM	CBD (3 mg/kg) mean ± SEM		
PFC: CB_1_R	Female	Tat(−)	0.20 ± 0.01	0.26 ± 0.01	n.s.	n.s.
		Tat(+)	0.19 ± 0.02	0.10 ± 0.01		
	Male	Tat(−)	0.41 ± 0.04	0.43 ± 0.05		
		Tat(+)	0.33 ± 0.01	0.35 ± 0.01		
PFC: CB_2_R	Female	Tat(−)	1.23 ± 0.12	1.69 ± 0.14	**sex x geno,** ***p* < 0.001** **geno x treat,** ***p* = 0.04**	Male Tat(+)> Male Tat(−), *p* = 0.002 Male Tat(+)> Female Tat(−), *p* < 0.001 Male Tat(+)> Female Tat(+), *p* < 0.001 Male Tat(−)> Female Tat(+), *p* = 0.02 n.s.
		Tat(+)	1.39 ± 0.16	1.19 ± 0.07		
	Male	Tat(−)	1.68 ± 0.07	1.73 ± 0.09		
		Tat(+)	2.31 ± 0.18	2.19 ± 0.21		
PFC: GPR55	Female	Tat(−)	0.006 ± 0.00	0.009 ± 0.00	**sex x geno,** ***p* = 0.003** **geno x treat, *p* < 0.001** **sex x geno x treat,** ***p* < 0.001**	Male Tat(−)> Female Tat(−), *p* < 0.001 Male Tat(−)> Female Tat(+), *p* < 0.001 Male Tat(+)> Female Tat(−), *p* < 0.001 Male Tat(+)> Female Tat(+), *p* < 0.001 Tat(−) CBD > Tat(+) CBD, *p* = 0.04 Male Tat(−) CBD > Male Tat(−) Veh, *p* < 0.001 Male Tat(−) CBD > Male Tat(+) CBD, *p* < 0.001 Male Tat(−) CBD > Male Tat(+) Veh, *p* < 0.001 Male Tat(−) CBD > Female Tat(−) Veh, *p* < 0.001 Male Tat(−) CBD > Female Tat(−) CBD, *p* < 0.001 Male Tat(−) CBD > Female Tat(+) Veh, *p* < 0.001 Male Tat(−) CBD > Female Tat(+) CBD, *p* < 0.001 Male Tat(+) Veh > Female Tat(−) Veh, *p* < 0.001 Male Tat(+) Veh > Female Tat(−) CBD, *p* < 0.001 Male Tat(+) Veh > Female Tat(+) Veh, *p* < 0.001 Male Tat(+) Veh > Female Tat(+) CBD, *p* < 0.001 Male Tat(+) Veh > Male Tat(+) CBD, *p* = 0.001 Male Tat(+) CBD > Female Tat(−) Veh, *p* < 0.01 Male Tat(−) Veh > Female Tat(−) Veh, *p* < 0.001 Male Tat(−) Veh > Female Tat(−) CBD, *p* < 0.001 Male Tat(−) Veh > Female Tat(+) Veh, *p* 0.007 Male Tat(−) Veh > Female Tat(+) CBD, *p* < 0.001
		Tat(+)	0.01 ± 0.00	0.009 ± 0.00		
	Male	Tat(−)	0.02 ± 0.00	0.04 ± 0.00		
		Tat(+)	0.03 ± 0.00	0.02 ± 0.00		
PFC: FAAH	Female	Tat(−)	0.62 ± 0.04	0.66 ± 0.04	**sex x geno,** ***p* = 0.01** **geno x treat, *p* = 0.03**	Female Tat(−)> Male Tat(−), *p* = 0.05 Female Tat(+)> Male Tat(−), *p* < 0.001 Male Tat(+)> Female Tat(−), *p* = 0.03 Male Tat(+)> Male Tat(−), *p* < 0.001 Tat(+) Veh > Tat(−) CBD, *p* = 0.002 Tat(+) Veh > Tat(−) Veh, *p* < 0.001 Tat(+) CBD > Tat(−) Veh, *p* = 0.007
		Tat(+)	0.80 ± 0.16	0.76 ± 0.04		
	Male	Tat(−)	0.34 ± 0.02	0.51 ± 0.48		
		Tat(+)	0.98 ± 0.07	0.74 ± 0.75		
PFC: MAGL	Female	Tat(−)	1.93 ± 0.11	1.91 ± 0.13	n.s.	n.s.
		Tat(+)	2.60 ± 0.60	1.98 ± 0.14		
	Male	Tat(−)	2.94 ± 0.36	2.65 ± 0.23		
		Tat(+)	2.84 ± 0.15	2.67 ± 0.12		
Amg: CB_1_R	Female	Tat(−)	0.61 ± 0.03	0.52 ± 0.30	**sex x geno,** ***p* < 0.001**	Female Tat(−)> Female Tat(+), *p* < 0.001 Female Tat(−)> Male Tat(−), *p* < 0.001 Male Tat(+)> Female Tat(+), *p* < 0.001 Male Tat(+)> Male Tat(−), *p* < 0.001
		Tat(+)	0.33 ± 0.02	0.37 ± 0.05		
	Male	Tat(−)	0.27 ± 0.02	0.31 ± 0.00		
		Tat(+)	0.61 ± 0.05	0.58 ± 0.03		
Amg: CB_2_R	Female	Tat(−)	0.98 ± 0.04	1.70 ± 0.18	**sex x geno,** ***p* = 0.004** **sex x treat,** ***p* = 0.001** **geno x treat,** ***p* = 0.02**	Female Tat(−)> Female Tat(+), *p* = 0.006 Male Tat(+)> Female Tat(+), *p* = 0.004 Male Tat(−)> Female Tat(+), *p* = 0.01 Female CBD > Female Veh, *p* = 0.002 Male Veh > Female Veh, *p* = 0.002 Male CBD > Female Veh, *p* = 0.01 Tat(−) CBD > Tat(−) Veh, *p* = 0.03 Tat(−) CBD > Tat(+) Veh, *p* = 0.03 Tat(−) CBD > Tat(+) CBD, *p* = 0.03
		Tat(+)	0.65 ± 0.07	1.02 ± 0.13		
	Male	Tat(−)	1.22 ± 0.05	1.39 ± 0.15		
		Tat(+)	1.55 ± 0.19	1.19 ± 0.10		
Amg: GPR55	Female	Tat(−)	0.10 ± 0.00	0.11 ± 0.00	**sex x geno,** ***p* < 0.001** **sex x treat,** ***p* = 0.04**	Female Tat(−)> Female Tat(+), *p* < 0.001 Female Tat(−)> Male Tat(−), *p* < 0.001 Female Tat(−)> Male Tat(+), *p* < 0.001 Male Tat(−)> Female Tat(+), *p* < 0.001 Male Tat(+)> Female Tat(+), *p* = 0.01 n.s.
		Tat(+)	0.01 ± 0.00	0.02 ± 0.00		
	Male	Tat(−)	0.05 ± 0.00	0.03 ± 0.00		
		Tat(+)	0.04 ± 0.00	0.03 ± 0.00		
Amg: FAAH	Female	Tat(−)	0.82 ± 0.08	0.99 ± 0.09	**sex x geno,** ***p* = 0.01** **sex x treat,** ***p* = 0.01**	Female Tat(−)> Female Tat(+), *p* < 0.001 Female Tat(−)> Male Tat(+), *p* = 0.01 n.s.
		Tat(+)	0.39 ± 0.03	0.52 ± 0.08		
	Male	Tat(−)	0.70 ± 0.06	0.66 ± 0.09		
		Tat(+)	0.76 ± 0.17	0.41 ± 0.05		
Amg: MAGL	Female	Tat(−)	1.97 ± 0.27	2.11 ± 0.11	**sex x geno,** ***p* < 0.001**	Female Tat(−)> Female Tat(+), *p* < 0.001 Female Tat(−)> Male Tat(−), *p* < 0.001 Female Tat(−)> Male Tat(+), *p* < 0.001 Female Tat(+)> Male Tat(−), *p* < 0.001 Female Tat(+)> Male Tat(+), *p* < 0.001
		Tat(+)	1.22 ± 0.09	1.39 ± 0.16		
	Male	Tat(−)	0.60 ± 0.06	0.71 ± 0.06		
		Tat(+)	0.80 ± 0.06	0.77 ± 0.11		
Spinal cord: CB_1_R	Female	Tat(−)	0.47 ± 0.04	0.47 ± 0.03	n.s.	n.s.
		Tat(+)	0.44 ± 0.03	0.47 ± 0.02		
	Male	Tat(−)	0.54 ± 0.06	0.64 ± 0.05		
		Tat(+)	0.54 ± 0.05	0.61 ± 0.03		
Spinal cord: CB_2_R	Female	Tat(−)	0.36 ± 0.03	0.50 ± 0.02	**sex x geno,** ***p* < 0.001** **geno x treat,** ***p* < 0.001** **sex x geno x treat,** ***p* = 0.007**	Male Tat(−)> Female Tat(−), *p* < 0.001 Male Tat(−)> Female Tat(+), *p* < 0.001 Male Tat(−)> Male Tat(+), *p* < 0.001 Tat(−) CBD > Tat(+) CBD, *p* = 0.01 Tat(−) CBD > Tat(−) Veh, *p* = 0.05 Male Tat(−) CBD > Female Tat(−) CBD, *p* < 0.001 Male Tat(−) CBD > Female Tat(−) Veh, *p* < 0.001 Male Tat(−) CBD > Female Tat(+) CBD, *p* < 0.001 Male Tat(−) CBD > Female Tat(+) Veh, *p* < 0.001 Male Tat(−) CBD > Male Tat(−) Veh, *p* < 0.001 Male Tat(−) CBD > Male Tat(+) CBD, *p* < 0.001 Male Tat(−) CBD > Male Tat(+) Veh, *p* < 0.001 Male Tat(−) Veh > Female Tat(−) Veh, *p* = 0.009
		Tat(+)	0.65 ± 0.04	0.60 ± 0.04		
	Male	Tat(−)	0.96 ± 0.17	1.77 ± 0.17		
		Tat(+)	0.82 ± 0.15	0.56 ± 0.03		
Spinal cord: GPR55	Female	Tat(−)	0.36 ± 0.03	0.03 ± 0.00	**geno x treat,** ***p* < 0.001**	n.s.
		Tat(+)	0.02 ± 0.00	0.02 ± 0.00		
	Male	Tat(−)	0.02 ± 0.00	0.04 ± 0.00		
		Tat(+)	0.05 ± 0.00	0.03 ± 0.00		
Spinal cord: FAAH	Female	Tat(−)	0.34 ± 0.01	0.28 ± 0.01	**sex x geno,** ***p* < 0.001** **sex x geno x treat,** ***p* < 0.001**	Male Tat(−)> Male Tat(+), *p* < 0.001 Female Tat(−)> Male Tat(+), *p* = 0.006 Female Tat(+)> Male Tat(+), *p* = 0.02 Male Tat(−) CBD > Female Tat(−) CBD, *p* = 0.008 Male Tat(−) CBD > Female Tat(+) CBD, *p* = 0.03 Male Tat(−) CBD > Female Tat(+) Veh, *p* = 0.008 Male Tat(−) CBD > Male Tat(−) Veh, *p* = 0.001 Male Tat(−) CBD > Male Tat(+) CBD, *p* < 0.001 Male Tat(−) CBD > Male Tat(+) Veh, *p* < 0.001 Female Tat(−) Veh > Male Tat(+) CBD, *p* = 0.003 Female Tat(+) CBD > Male Tat(+) CBD, *p* = 0.03
		Tat(+)	0.28 ± 0.02	0.30 ± 0.01		
	Male	Tat(−)	0.29 ± 0.04	0.44 ± 0.05		
		Tat(+)	0.22 ± 0.02	0.16 ± 0.00		
Spinal cord: MAGL	Female	Tat(−)	0.65 ± 0.16	0.41 ± 0.02	n.s.	n.s.
		Tat(+)	0.28 ± 0.01	0.29 ± 0.01		
	Male	Tat(−)	0.46 ± 0.03	0.73 ± 0.20		
		Tat(+)	0.33 ± 0.02	0.33 ± 0.03		
Brainstem: CB_1_R	Female	Tat(−)	0.27 ± 0.05	0.31 ± 0.01	n.s.	n.s.
		Tat(+)	0.24 ± 0.02	0.22 ± 0.01		
	Male	Tat(−)	0.50 ± 0.05	0.61 ± 0.11		
		Tat(+)	0.55 ± 0.04	0.58 ± 0.05		
Brainstem: CB_2_R	Female	Tat(−)	1.45 ± 0.06	1.35 ± 0.05	n.s.	n.s.
		Tat(+)	1.13 ± 0.05	1.08 ± 0.03		
	Male	Tat(−)	1.66 ± 0.11	1.76 ± 0.08		
		Tat(+)	1.77 ± 0.13	1.55 ± 0.16		
Brainstem: GPR55	Female	Tat(−)	0.03 ± 0.00	0.03 ± 0.00	**sex x treat,** ***p* < 0.001** **sex x geno x treat,** ***p* = 0.05**	Female Tat(−)> Male Tat(−), *p* < 0.001 Female Tat(−)> Male Tat(+), *p* < 0.001 Female Tat(+)> Female Tat(−), *p* = 0.02 Female Tat(+)> Male Tat(−), *p* < 0.001 Female Tat(+)> Male Tat(+), *p* < 0.001 Female Tat(+) Veh > Female Tat(−) Veh, *p* = 0.03 Female Tat(+) Veh > Male Tat(−) CBD, *p* < 0.001 Female Tat(+) Veh > Male Tat(−) Veh, *p* < 0.001 Female Tat(+) Veh > Male Tat(+) CBD, *p* < 0.001 Female Tat(+) Veh > Male Tat(+) Veh, *p* < 0.001 Female Tat(+) CBD > Male Tat(−) CBD, *p* < 0.001 Female Tat(+) CBD > Male Tat(−) Veh, *p* < 0.001 Female Tat(+) CBD > Male Tat(+) CBD, *p* < 0.001 Female Tat(+) CBD > Male Tat(+) Veh, *p* < 0.001 Female Tat(−) CBD > Male Tat(−) CBD, *p* < 0.001 Female Tat(−) CBD > Male Tat(−) Veh, *p* = 0.01 Female Tat(−) CBD > Male Tat(+) CBD, *p* < 0.001 Female Tat(−) CBD > Male Tat(+) Veh, *p* < 0.001 Female Tat(−) Veh > Male Tat(−) CBD, *p* = 0.02 Female Tat(−) Veh > Male Tat(+) CBD, *p* = 0.003 Female Tat(−) Veh > Male Tat(+) Veh, *p* = 0.002
		Tat(+)	0.04 ± 0.00	0.04 ± 0.00		
	Male	Tat(−)	0.02 ± 0.00	0.02 ± 0.00		
		Tat(+)	0.01 ± 0.00	0.01 ± 0.00		
Brainstem: FAAH	Female	Tat(−)	0.27 ± 0.01	0.28 ± 0.01	**geno x treat,** ***p* = 0.004**	Tat(−) CBD > Tat(+) Veh, *p* = 0.008 Tat(−) CBD > Tat(+) CBD, *p* < 0.001 Tat(−) Veh > Tat(+) CBD, *p* < 0.001 Tat(+) CBD > Tat(+) CBD, *p* = 0.03
		Tat(+)	0.21 ± 0.01	0.19 ± 0.00		
	Male	Tat(−)	0.29 ± 0.01	0.32 ± 0.01		
		Tat(+)	0.28 ± 0.01	0.21 ± 0.02		
Brainstem: MAGL	Female	Tat(−)	0.57 ± 0.10	0.51 ± 0.02	**sex x treat,** ***p* = 0.04** **geno x treat,** ***p* = 0.05** **sex x geno x treat,** ***p* = 0.02**	n.s. Tat(−) CBD > Tat(+) CBD, *p* < 0.001 Tat(−) CBD > Tat(+) Veh, *p* < 0.001 Male Tat(−) CBD > Female Tat(−) CBD, *p* = 0.01 Male Tat(−) CBD > Female Tat(+) CBD, *p* < 0.001 Male Tat(−) CBD > Female Tat(+) Veh, *p* = 0.002 Male Tat(−) CBD > Male Tat(−) Veh, *p* = 0.002 Male Tat(−) CBD > Male Tat(+) CBD, *p* < 0.001 Male Tat(−) CBD > Male Tat(+) Veh, *p* < 0.001
		Tat(+)	0.34 ± 0.04	0.33 ± 0.01		
	Male	Tat(−)	0.45 ± 0.04	0.91 ± 0.17		
		Tat(+)	0.30 ± 0.02	0.27 ± 0.01		

Amg, amygdala; CB_1_R, cannabinoid receptor type 1; CB_2_R, cannabinoid receptor type 2; CBD, cannabidiol; CNS, central nervous system; FAAH, fatty acid amide hydrolase; geno, genotype; MAGL, monoacylglycerol lipase; n.s., not significant; PFC, prefrontal cortex; Tat, transactivator of transcription; geno, genotype; treat, treatment; Veh, vehicle

For total exploration time (Fig 2B), a three-way ANOVA revealed a significant genotype x treatment interaction [*F*(1,54) = 6.94, *p* = 0.01]. Specifically, vehicle treated Tat(+) mice exhibited higher exploration times compared to their vehicle treated Tat(–) counterparts, an effect that was not observed following chronic CBD treatment. Regarding the DI (Fig 2C), significant main effects were observed for both genotype [*F*(1,54) = 16.15, *p* < 0.001] and treatment [*F*(1,54) = 10.83, *p* = 0.002]. Overall, Tat(–) mice and CBD treated mice demonstrated a stronger preference for the novel object compared to Tat(+) and vehicle-treated mice, respectively. Additionally, a significant sex x treatment interaction was observed for DI [*F*(1,54) = 10.83, *p* = 0.002] with treatment differentially affecting the object preference based on sex.

To determine if behavioral findings were associated with biochemical alterations, we quantified eCB levels and protein expression within the PFC. Analysis of AEA levels revealed no significant effects or interactions (Fig 2D). In contrast, 2-AG levels were subject to a significant three-way sex x genotype x treatment interaction [*F*(1,54) = 11.84, *p* = 0.001; Fig 2E], indicating that the impact of chronic treatment on 2-AG was uniquely dependent upon the combination of sex and Tat status. For AA levels (Fig 2F), no significant main effects were observed; however, the data revealed two significant interactions. A significant sex x treatment interaction [*F*(1,54) = 4.68, *p* = 0.03] suggested that CBD differentially modulated AA levels in males versus females. Furthermore, a significant genotype x treatment interaction [*F*(1,54) = 10.43, *p* = 0.002] was driven by chronic CBD treatment significantly decreasing AA levels in Tat(+) mice relative to Tat(–) mice, a differential effect that was absent in vehicle-treated mice.

Western blot analysis revealed distinct regulatory patterns for cannabinoid receptors (Fig 2G). For CB_1_R (Fig 2H), significant main effects of sex [*F*(1,54) = 74.6, *p* < 0.001] and genotype [*F*(1,54) = 12.13, *p* < 0.001] were observed, with higher expression found in males and Tat(–) mice compared to females and Tat(+) mice, respectively. For CB_2_R (Fig 2I), a significant main effect of sex [*F*(1,54) = 35.7, *p* < 0.001] showed higher expression in males, while a significant sex x genotype interaction [*F*(1,54) = 13.0, *p* < 0.001] indicated that the influence of the Tat on CB_2_R expression was sex specific. A genotype x treatment [*F*(1,54) = 4.25, *p* = 0.04] was also noted indicating that chronic CBD treatment differentially modulated CB_2_R protein levels depending on genotype.

GPR55 expression (Fig 2J) exhibited high sensitivity to all variables. Beyond a main effect of sex [*F*(1,54) = 161.1, *p* < 0.001] with males expressing higher GPR55 as compared to the females, we observed significant sex x genotype [*F*(1,54) = 9.48, *p* = 0.003] and genotype × treatment [*F*(1,54) = 36.1, *p* < 0.001] interactions. The latter reflected a genotype-specific shift in receptor expression following CBD exposure. Most notably, a significant sex x genotype x treatment interaction [*F*(1,54). = 19.86, *p* < 0.001] revealed that CBD’s effect on GPR55 was simultaneously governed by both sex and genotype.

Finally, we assessed the metabolic enzymes FAAH and MAGL. For FAAH (Fig 2K), a significant main effect of genotype [*F*(1,54) = 27.20, *p* < 0.001] showed higher expression in Tat(+) mice. This was further qualified by a genotype x sex interaction [*F*(1,54) = 6.66, *p* = 0.001] and a genotype x treatment interaction [*F*(1,54) = 4.83, *p* = 0.03]. Specifically, vehicle treatment resulted in elevated FAAH levels in Tat(+) mice compared to Tat(–) mice, but this genotype-driven difference was abolished by chronic CBD treatment. In contrast, MAGL expression was only influenced by a main effect of sex [*F*(1,54) = 10.41, *p* = 0.002; Fig 2L], with higher levels observed in males compared to females and no further significant interactions. Raw and unedited western blots for PFC shown in **S4_Fig**.

### Chronic CBD treatment modulates anxiety-like behavior and normalizes Tat-induced endocannabinoid dysregulation in the amygdala through sex-specific mechanisms

**Fig 3:**
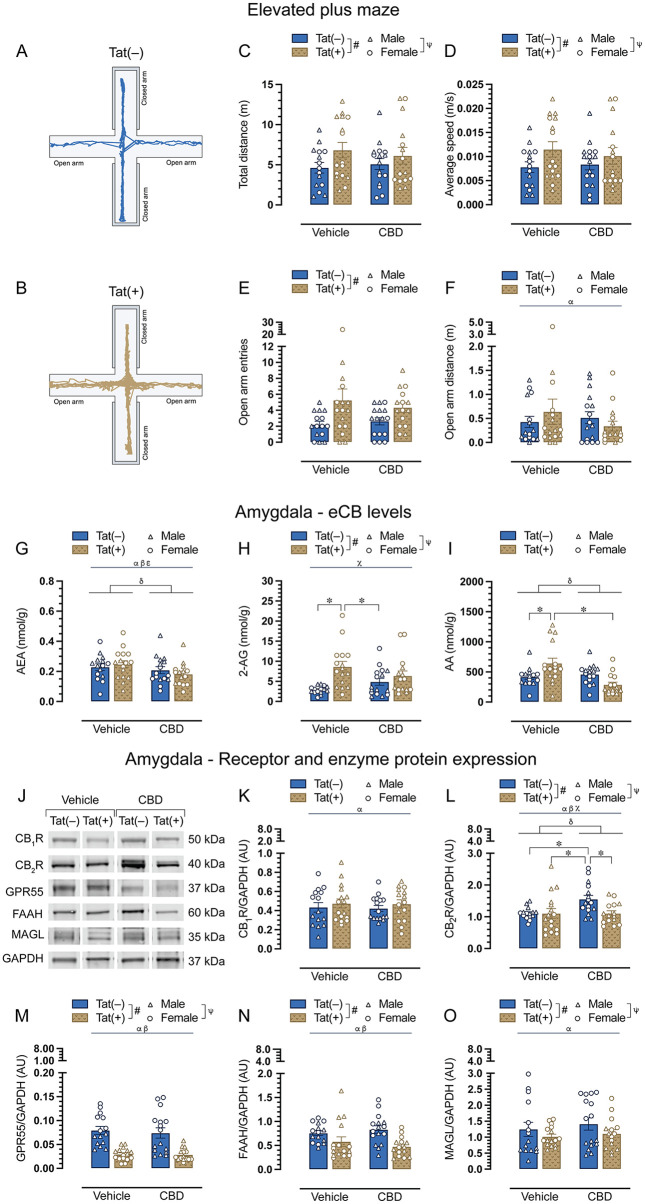
Chronic CBD treatment and Tat expression alter anxiety-like behavior and endocannabinoid signaling within the amygdala. (A) Representative movement trace of a Tat(–) mouse in the elevated plus maze (EPM) apparatus. (B) Representative movement trace of a Tat(+) mouse in the EPM. (C) Total distance traveled in the EPM; males and Tat(+) mice exhibited higher locomotor activity compared to females and Tat(–) counterparts. (D) Average speed in the EPM; average speed was significantly higher in male and Tat(+) groups. (E) Open arm entries; Tat(+) mice entered the open arms more frequently than Tat(–) mice. (F) Open arm distance; distance in the open arms was differentially influenced by a sex x genotype interaction. (G) AEA levels in the amygdala; levels were governed by a three-way interaction between sex, genotype, and treatment, with CBD generally reducing AEA compared to vehicle. (H) 2-AG levels in the amygdala; CBD treatment abolished the Tat-induced elevation of 2-AG observed in vehicle-treated mice. (I) AA levels in the amygdala; CBD treatment significantly lowered AA levels and eliminated genotype driven differences seen in the vehicle group. (J) Representative Western blot bands for CB_1_R, CB_2_R, GPR55, FAAH, MAGL, and loading control GAPDH. (K) CB_1_R protein expression; levels were contingent upon a significant sex x genotype interaction. (L) CB_2_R protein expression; CBD treatment increased expression, though a genotype × treatment interaction revealed this effect was more pronounced in Tat(–) mice. (M) GPR55 protein expression; levels were higher in males and Tat(–) mice, with receptor density modulated by sex-specific interactions with genotype and treatment. (N) FAAH protein expression; Tat(–) mice exhibited higher expression than Tat(+) mice, with levels refined by sex-specific responses to treatment. (O) MAGL protein expression; levels were significantly higher in females and Tat(–) mice, with the impact of the Tat transgene being fundamentally dependent on the sex of the animal. Data represented as mean ± SEM. Statistical significance was assessed by overall ANOVAs, ^#^*p* < 0.05 main effect of genotype, ^ψ^*p* < 0.05 main effect of sex, ^δ^*p* < 0.05 main effect of treatment, ^α^*p* < 0.05 sex x genotype interaction, ^β^*p* < 0.05 sex x treatment interaction, ^χ^*p* < 0.05 genotype x treatment interaction, ^ε^*p* < 0.05 sex x genotype x treatment interaction. CBD dose = 3 mg/kg. *N* = 62(31f).

To evaluate the effects of chronic CBD treatment and Tat expression on anxiety-like behavior, mice were tested in the elevated plus maze [EPM; Fig 3A for Tat(–) and Fig 3B) for Tat(+)]. Additionally, eCB and protein levels were quantified in the amygdala to assess cellular level changes associated with this behavior. Assessment of total distance (Fig 3C) within the maze revealed significant main effects of sex [*F*(1,54) = 4.24, *p* = 0.04] and genotype [*F*(1,54) = 3.91, *p* = 0.05]. Specifically, males and Tat(+) mice traveled a significantly greater distance than females and Tat(–) mice, respectively. Similarly, for average speed (Fig 3D), a main effect of sex [*F*(1,54) = 4.33, *p* = 0.04] and genotype [*F*(1,54) = 3.96, *p* = 0.05] was found with males and Tat(+) mice traveling more as compared to females and Tat(–) mice, respectively. With regards to anxiety-like metrics, a significant main effect of genotype was observed for the total number of open arm entries [*F*(1,54) = 7.97, *p* = 0.007; Fig 3E]. Tat(+) mice entered the open arms more frequently than Tat(–) mice, suggesting a reduction in anxiety-like behavior in the presence of the Tat transgene. Lastly, analysis of total open arm distance (Fig 3F) revealed a significant sex x genotype interaction [*F*(1,54) = 4.03, *p* = 0.04], indicating that the distance traveled within the open arms was differentially influenced by genotype depending on the sex of the animal.

To investigate the biochemical correlates of the observed behavioral phenotypes, AEA, 2-AG, and AA levels were quantified within the amygdala. For AEA (Fig 3G), a significant main effect of treatment was observed [*F*(1,54) = 4.62, *p* = 0.03], with vehicle-treated mice exhibiting higher levels compared to the CBD group. This was further supported by significant sex x genotype [*F*(1,54) = 9.15, *p* = 0.004] and sex x treatment [*F*(1,54) = 12.48, *p* < 0.001] interactions. Ultimately, a significant sex x genotype x treatment interaction [*F*(1,54) = 4.17, *p* = 0.04] was found, indicating that AEA levels in the amygdala are governed by a complex interplay between sex, genotype, and chronic CBD exposure. Analysis of 2-AG (Fig 3H) revealed significant main effects of sex [*F*(1,54) = 8.09, *p* = 0.006] and genotype [*F*(1,54) = 14.4, *p* < 0.001], characterized by higher levels in females and Tat(+) mice compared to males and Tat(–) mice, respectively. Additionally, a significant genotype x treatment interaction was noted [*F*(1,54) = 5.40, *p* = 0.02]. Specifically, vehicle-treated Tat(+) mice showed elevated 2-AG levels relative to vehicle-treated Tat(–) mice, a genotype-driven disparity that was abolished following chronic CBD administration. For AA (Fig 3I), a significant main effect of treatment was observed [*F*(1,54) = 8.44, *p* = 0.005], with CBD treatment leading to significantly lower levels compared to vehicle-treated mice. Similar to the pattern observed for 2-AG, AA levels exhibited a significant genotype x treatment interaction [*F*(1,54) = 14.14, *p* < 0.001]. In the vehicle group, Tat(+) mice showed significantly higher AA levels than Tat(–) mice, whereas no such difference was detected in CBD-treated mice. Finally, a significant sex x genotype x treatment [*F*(1,54) = 4.04, *p* = 0.04] interaction was observed, suggesting that AA alterations in the amygdala are simultaneously modulated by all three independent variables.

Protein expression analysis (Fig 3J) within the amygdala revealed distinct regulatory profiles for the primary cannabinoid receptors and their associated metabolic enzymes. For CB_1_R (Fig 3K), no main effects were observed; however, a significant sex x genotype interaction [*F*(1,54) = 91.57, *p* < 0.001] was detected, indicating that CB_1_R levels are contingent upon the specific combination of host sex and Tat status. Analysis of CB_2_R (Fig 3L) revealed significant main effects for all three factors: sex [*F*(1,54) = 6.94, *p* = 0.01], genotype [*F*(1,54) = 5.40, *p* = 0.02], and treatment [*F*(1,54) = 5.87, *p* = 0.01]. Specifically, expression was higher in males, Tat(–) mice, and CBD-treated mice compared to their respective counterparts. These effects were contextualized by significant interactions across all factors: sex x genotype [*F*(1,54) = 9.27, *p* = 0.004], sex x treatment [*F*(1,54) = 12.0, *p* = 0.001], and genotype x treatment [*F*(1,54) = 5.73, *p* = 0.02]. Notably, the genotype x treatment interaction was driven by Tat(–) CBD-treated mice expressing higher CB_2_R levels than Tat(+) CBD-treated mice, a disparity that was absent in vehicle-treated mice. For GPR55 (Fig 3M), significant main effects of sex [*F*(1,54) = 38.77, *p* < 0.001] and genotype [*F*(1,54) = 163.5, *p* < 0.001] were observed, with higher expression in males and Tat(–) mice. These findings were modulated by a significant sex x genotype interaction [*F*(1,54) = 114.0, *p* < 0.001] and a sex x treatment interaction [*F*(1,54) = 4.13, *p* = 0.04], suggesting that GPR55 density in males and females is differentially sensitive to both genotype and chronic CBD exposure. Regarding FAAH expression (Fig 3N), a significant main effect of genotype [*F*(1,54) = 16.14, *p* < 0.001] was found, with Tat(–) mice exhibiting higher levels than Tat(+) mice. This relationship was refined by significant sex x genotype [*F*(1,54) = 6.81, *p* = 0.01] and sex x treatment [*F*(1,54) = 6.73, *p* = 0.01] interactions, reflecting sex-specific responses to the Tat transgene and CBD treatment. Finally, MAGL expression (Fig 3O) was influenced by significant main effects of sex [*F*(1,54) = 107.8, *p* < 0.001] and genotype [*F*(1,54) = 10.64, *p* = 0.002], with higher levels found in females and Tat(–) mice. These main effects were superseded by a significant sex x genotype interaction [*F*(1,54) = 22.32, *p* < 0.001], indicating that the impact of the Tat transgene on MAGL levels is fundamentally different between males and females. Raw and unedited western blots for amygdala shown in **S6_Fig**.

### Chronic CBD treatment influence nociceptive processing and regulates spinal cord cannabinoid receptor expression and endocannabinoid tone

**Fig 4:**
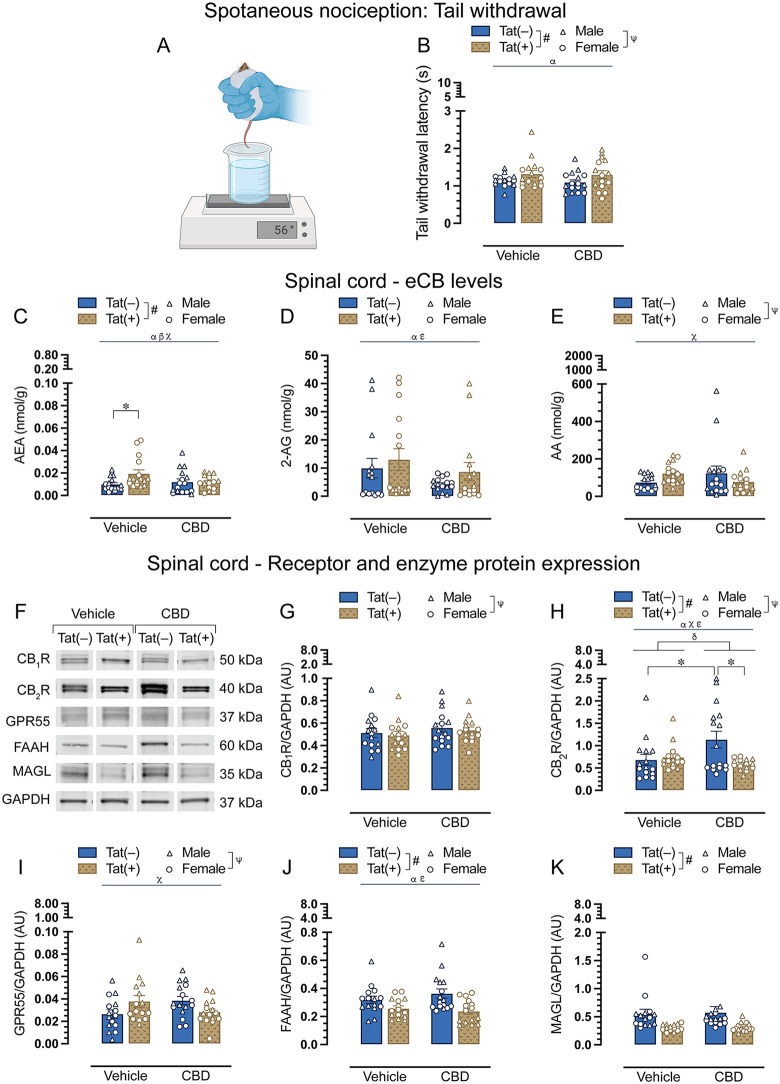
Chronic CBD treatment and Tat expression modulate thermal nociception and endocannabinoid signaling within the spinal cord. (A) Schematic representation of the tail withdrawal assay used to assess thermal nociceptive sensitivity. (B) Tail withdrawal latency; males and Tat(+) mice exhibited significantly higher latencies compared to females and Tat(–) counterparts. (C) AEA levels in the spinal cord; levels were significantly higher in Tat(+) mice and were subject to complex interactions between sex, genotype, and treatment. (D) 2-AG levels in the spinal cord; were uniquely governed by a significant interaction of sex, genotype, and CBD treatment. (E) AA levels in the spinal cord; levels were significantly higher in males compared to females and were differentially modulated by a genotype x treatment interaction. (F) Representative western blot bands for CB_1_R, CB_2_R, GPR55, FAAH, MAGL, and loading control GAPDH. (G) CB_1_R protein expression; levels were significantly higher in males than in females across all groups. (H) CB_2_R protein expression; CBD treatment generally increased expression and were dependent on an interaction between all factors. (I) GPR55 protein expression; expression was higher in males and was differentially affected by the interplay between the Tat transgene and chronic CBD treatment. (J) FAAH protein expression; levels were higher in Tat(–) mice and were refined by a significant three-way interaction of sex, genotype, and treatment. (K) MAGL protein expression; Tat(–) mice exhibited significantly higher levels compared to Tat(+) mice regardless of sex or treatment. Data represented as mean ± SEM. Statistical significance was assessed by overall ANOVAs, ^#^*p* < 0.05 main effect of genotype, ^ψ^*p* < 0.05 main effect of sex, ^δ^*p* < 0.05 main effect of treatment, ^α^*p* < 0.05 sex x genotype interaction, ^β^*p* < 0.05 sex x treatment interaction, ^χ^*p* < 0.05 genotype x treatment interaction, ^ε^*p* < 0.05 sex x genotype x treatment interaction. CBD dose = 3 mg/kg. *N* = 62(31f).

To evaluate the impact of chronic CBD treatment on spontaneous nociception, tail withdrawal latency was assessed (Fig 4A). Additionally, eCB and protein levels were quantified within the spinal cord to identify the biochemical correlates of these nociceptive responses. Analysis of tail withdrawal latency revealed significant main effects of sex [*F*(1,54) = 13.12, *p* < 0.001] and genotype [*F*(1,54) = 7.42, *p* = 0.009; Fig 4B]. Overall, males and Tat(+) mice exhibited higher latency thresholds, indicating they were less sensitive to thermal stimuli compared to females and Tat(–) mice, respectively. These main effects were contextualized by a significant sex x genotype interaction [*F*(1,54) = 6.32, *p* = 0.01], which suggested that the influence of the Tat expression on nociceptive sensitivity was fundamentally different between males and females.

To explore the biochemical environment of the spinal cord in relation to nociceptive sensitivity, AEA, 2-AG, and AA levels were quantified. For AEA (Fig 4C), a significant main effect of genotype was observed [*F*(1,54) = 4.83, *p* = 0.03], with Tat(+) mice exhibiting higher levels compared to Tat(–) mice. This main effect was modulated by a series of significant interactions, including sex x genotype [*F*(1,54) = 7.24, *p* = 0.009], sex x treatment [*F*(1,54) = 7.01, *p* = 0.01], and genotype × treatment [*F*(1,54) = 5.25, *p* = 0.02]. These findings suggest that AEA concentration in the spinal cord is contingent upon a delicate balance between host sex, genotype, and chronic CBD exposure. While no significant main effects were detected for 2-AG (Fig 4D), the data revealed a significant sex x genotype interaction [*F*(1,54) = 4.55, *p* = 0.03]. This was further contextualized by a significant sex x genotype x treatment interaction [*F*(1,54) = 38.81, *p* < 0.001], indicating that 2-AG levels in the spinal cord are simultaneously governed by the interplay of all three independent variables. For AA (Fig 4E), a significant main effect of sex [*F*(1,54) = 7.02, *p* = 0.01] was found, with males maintaining higher levels in the spinal cord compared to females. Additionally, a significant genotype x treatment interaction [*F*(1,54) = 4.93, *p* = 0.03] was observed, suggesting that AA levels are differentially modulated by chronic CBD treatment depending on the animal’s Tat status.

Western blot analysis of the spinal cord (Fig 4F) revealed distinct regulatory patterns for cannabinoid receptors and metabolic enzymes, characterized by prominent sex and genotype differences alongside CBD mediated changes. For CB_1_R (Fig 4G), a significant main effect of sex was observed [*F*(1,54) = 12.98, *p* < 0.001], with males exhibiting higher expression levels compared to females. No other significant main effects or interactions were detected for this receptor. In contrast, CB_2_R expression (Fig 4H) was influenced by significant main effects of sex [*F*(1,54) = 41.05, *p* < 0.001], genotype [*F*(1,54) = 9.22, *p* = 0.004], and treatment [*F*(1,54) = 3.95, *p* = 0.05]. Higher levels of CB_2_R were found in males, Tat(–) mice, and CBD treated mice compared to their respective female, Tat(+), and vehicle treated counterparts. These effects were superseded by a significant interactions for sex x genotype [*F*(1,54) = 30.73, *p* < 0.001] and genotype x treatment [*F*(1,54) = 16.39, *p* < 0.001], alongside a sex x genotype x treatment interaction [*F*(1,54) = 8.00, *p* = 0.007]. This indicates that CB_2_R expression in the spinal cord is contingent upon specific combinations of all three independent variables. For GPR55 (Fig 4I), a significant main effect of sex was observed [*F*(1,54) = 14.64, *p* < 0.001], with males expressing higher levels than females. This relationship was modulated by a significant genotype x treatment interaction [*F*(1,54) = 9.84, *p* = 0.003], indicating that GPR55 expression is differentially regulated by the Tat transgene and chronic CBD exposure.

FAAH expression in the spinal cord (Fig 4J) showed a significant main effect of genotype [*F*(1,54) = 20.82, *p* < 0.001], with Tat(–) mice exhibiting higher levels than Tat(+) mice. This effect was refined by a significant sex x genotype x treatment interaction [*F*(1,54) = 13.30, *p* < 0.001], suggesting that FAAH levels are sensitive to the interplay between sex, genotype, and treatment status. Finally, MAGL expression (Fig 4K) demonstrated a significant main effect of genotype [*F*(1,54) = 14.40, *p* < 0.001], where Tat(–) mice maintained higher levels in the spinal cord compared to Tat(+) mice. No additional significant interactions were observed for MAGL. Raw and unedited western blots for spinal cord shown in **S7_Fig**.

### Chronic CBD administration selectively modulates supraspinal nociceptive sensitivity in Tat(+) mice and restructures the brainstem endocannabinoid profile

**Fig 5:**
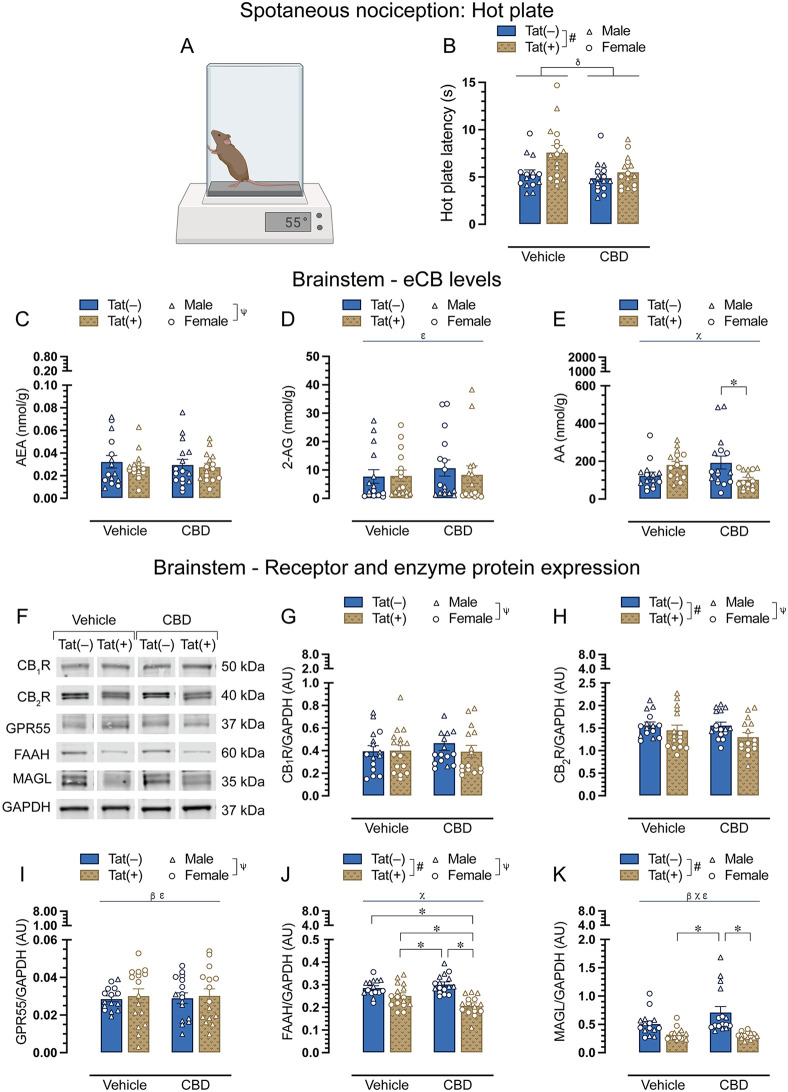
Chronic CBD treatment and Tat expression influence supraspinal nociception and biochemical signaling within the brainstem. (A) Schematic representation of the hot plate assay used to evaluate supraspinal thermal nociceptive thresholds. (B) Hot plate latency; significant main effects of genotype and treatment were observed, with Tat(–) and CBD-treated mice exhibiting higher sensitivity (shorter latencies) compared to Tat(+) and vehicle groups. (C) AEA levels in the brainstem; a prominent main effect of sex was detected, with males showing significantly higher levels than females. (D) 2-AG levels in the brainstem; levels were governed by a complex interaction of sex, genotype, and CBD treatment. (E) AA levels in the brainstem; CBD treatment selectively reduced AA levels in Tat(+) mice compared to CBD-treated Tat(–) counterparts, a genotype-specific effect not seen in vehicle-treated mice. (F) Representative Western blot bands for CB_1_R, CB_2_R, GPR55, FAAH, MAGL, and loading control GAPDH. (G) CB_1_R protein expression; males exhibited significantly higher expression levels than females. (H) CB_2_R protein expression; significant main effects of sex and genotype were observed, with lower expression found in females and Tat(+) mice. (I) GPR55 protein expression; females expressed higher levels than males, with expression further modulated by a three-way interaction of all variables. (J) FAAH protein expression; CBD treatment significantly decreased FAAH expression specifically in Tat(+) mice relative to Tat(–) mice. (K) MAGL protein expression; Tat(–) mice showed higher expression than Tat(+) mice, with levels ultimately determined by an intricate interaction between sex, genotype, and treatment. Data represented as mean ± SEM. Statistical significance was assessed by overall ANOVAs, ^#^*p* < 0.05 main effect of genotype, ^ψ^*p* < 0.05 main effect of sex, ^δ^*p* < 0.05 main effect of treatment, ^α^*p* < 0.05 sex x genotype interaction, ^β^*p* < 0.05 sex x treatment interaction, ^χ^*p* < 0.05 genotype x treatment interaction, ^ε^*p* < 0.05 sex x genotype x treatment interaction. CBD dose = 3 mg/kg. *N* = 62(31f).

To further evaluate the impact of chronic CBD treatment on spontaneous nociception, mice were assessed using the hot plate assay (Fig 5A). Additionally, eCB levels and receptor expression were quantified within the brainstem to identify biochemical correlates associated with supraspinal nociceptive processing. Analysis of the hot plate assay (Fig 5B) revealed significant main effects of both genotype [*F*(1,54) = 7.37, *p* = 0.009] and treatment [*F*(1,54) = 5.36, *p* = 0.02]. Specifically, Tat(–) mice and CBD treated mice exhibited significantly shorter latencies or higher sensitivity compared to Tat(+) and vehicle treated counterparts. These results suggest that Tat expression and chronic vehicle treatment is associated with an increase in hot plate latency, significantly modulating thermal nociceptive thresholds. No further significant interactions were observed for hot plate latency.

To investigate the biochemical underpinnings of supraspinal nociception, AEA, 2-AG, and AA levels were quantified within the brainstem. For AEA (Fig 5C), no significant interactions were detected; however, a prominent main effect of sex was observed [*F*(1,54) = 13.80, *p* < 0.001]. Specifically, males exhibited significantly higher AEA concentrations in the brainstem compared to females. Analysis of 2-AG (Fig 5D) revealed a significant sex x genotype x treatment interaction [*F*(1,54) = 57.1, *p* < 0.001]. This result indicates that 2-AG levels within the brainstem are governed by an intricate and interdependent balance between the sex, the presence of the Tat transgene, and chronic CBD exposure. Regarding AA (Fig 5E), no significant main effects were observed. However, the data revealed a significant genotype x treatment interaction [F(1,54) = 11.24, p = 0.001]. This interaction was primarily driven by CBD’s selective effect on Tat status with chronic CBD treatment significantly decreased AA levels in Tat(+) mice compared to CBD treated Tat(–) counterparts, a differential effect that was absent in the vehicle treated control groups.

Western blot analysis of the brainstem (Fig 5F) was conducted to characterize the expression profiles of cannabinoid receptors and their primary metabolic enzymes. For CB_1_R (Fig 5G), a significant main effect of sex [*F*(1,54) = 54.03, *p* < 0.001] was identified, characterized by higher expression in males compared to females, with no other significant effects observed. Similarly, CB_2_R expression (Fig 5H) demonstrated main effects of sex [*F*(1,54) = 39.50, *p* < 0.001] and genotype [*F*(1,54) = 6.15, *p* = 0.01], with higher levels found in males and Tat(–) mice compared to females and Tat(+) mice, respectively. Analysis of GPR55 (Fig 5I) revealed a distinct pattern, with a significant main effect of sex [*F*(1,54) = 120.4, *p* < 0.001] showing that females expressed higher levels of the receptor than males. This was further contextualized by a significant sex x genotype interaction [*F*(1,54) = 14.5, *p* < 0.001] and a significant sex x genotype x treatment interaction [*F*(1,54) = 3.96, *p* = 0.05], indicating that GPR55 expression in the brainstem is fundamentally dependent on the interplay between all three independent variables.

Regarding FAAH expression (Fig 5J), significant main effects of sex [*F*(1,54) = 15.25, *p* < 0.001] and genotype [*F*(1,54) = 42.2, *p* < 0.001] were observed, with males and Tat(–) mice exhibiting higher FAAH expression than females and Tat(+) mice. These effects were further modulated by a significant genotype x treatment interaction [*F*(1,54) = 9.17, *p* = 0.004]. Specifically, chronic CBD treatment significantly reduced FAAH expression in Tat(+) mice relative to Tat(–) mice, a genotype-driven disparity that was not observed in the vehicle treated group. Finally, MAGL expression (Fig 5K) showed a significant main effect of genotype [*F*(1,54) = 29.40, *p* < 0.001], with higher expression in Tat(–) mice compared to Tat(+) mice. This relationship was superseded by significant two-way interactions for sex x treatment [*F*(1,54) = 4.93, *p* = 0.03] and genotype x treatment [*F*(1,54) = 4.00, *p* = 0.05], and ultimately a significant three-way sex x genotype x treatment interaction [*F*(1,54) = 5.77, *p* = 0.02]. This complex interaction highlights that MAGL levels in the brainstem are tuned by the specific combination of sex, Tat status, and CBD exposure. Raw and unedited western blots for brainstem shown in **S8_Fig**.

### Chronic CBD treatment increased baseline body temperature without affecting body mass and locomotor activity

**Fig 6:**
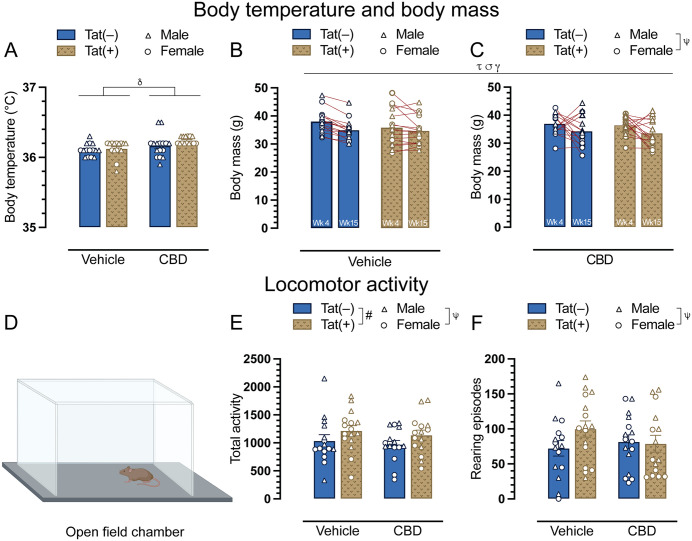
Physiological and locomotor activity effects of chronic CBD treatment in HIV-1 Tat tg mice. (A) Post-behavioral body temperature; chronic CBD increased baseline temperature regardless of sex or genotype. (B–C) Longitudinal body mass assessment at acute (week 4) and chronic (week 15) time points for vehicle (B) and CBD (C) treated mice where CBD treatment significantly reduced body mass over time, with effect dependent upon sex and genotype. (D) Schematic representation of the locomotor activity chamber. (E) Total locomotor activity; Tat(+) mice and male mice exhibited higher activity levels, though no effect of CBD treatment was observed. (F) Frequency of rearing behavior; males displayed significantly higher rearing episodes compared to females, with no significant impact from genotype or CBD treatment. Data represented as mean ± SEM. Statistical significance was assessed by overall ANOVAs, ^#^*p* < 0.05 main effect of genotype, ^ψ^*p* < 0.05 main effect of sex, ^δ^*p* < 0.05 main effect of treatment. A four-way mixed ANOVA for body mass; ^τ^*p* < 0.05 main effect of time, ^σ^*p* < 0.05 main time x sex x treatment interaction and ^γ^*p* < 0.05 main time x genotype x treatment interaction. CBD dose = 3 mg/kg. *N* = 62(31f).

Following the conclusion of behavioral testing, body temperature was assessed in all subjects (Fig 6A). Two-way ANOVA revealed a significant main effect of treatment [*F*(1, 54) = 8.68, *p* = 0.005] characterized by an elevation in baseline temperature in CBD-treated mice relative to the vehicle group, regardless of genotype or sex. Body mass was monitored weekly throughout the duration of CBD administration and behavioral testing (Fig 6B, C). A four-way mixed ANOVA comparing acute (week 4) and chronic (week 15) administration revealed a significant main effect of time [*F*(1,54) = 28.95, *p* < 0.001] with body mass decreasing over time across all groups. Further a significant time x treatment interaction [*F*(1,54) = 7.87, *p* = 0.007] was seen reflecting a significant reduction in body mass following chronic CBD treatment (week 15) compared to the acute phase (week 4). Additionally, the impact of chronic CBD was found to be dependent on both sex and genotype, as evidenced by significant time x sex x treatment [*F*(1,54) = 6.48, *p* = 0.01] and time x genotype x treatment [*F*(1,54) = 4.60, *p* = 0.03] interactions, respectively. These results indicate that while CBD generally reduced body mass over time and magnitude of this effect was differentially expressed based on sex and Tat expression.

Regarding locomotor activity, chronic CBD treatment had no significant effect on total activity across genotypes or sexes (Fig 6D, E). However, significant main effects were observed for both genotype [*F*(1, 54) = 9.87, *p* = 0.003] and sex [*F*(1, 54) = 4.45, *p* = 0.03] with Tat(+) mice and male mice exhibiting higher total activity compared to Tat(–) and female counterparts, respectively. Furthermore, analysis of exploratory behavior revealed a significant main effect of sex [*F*(1, 54) = 5.84, *p* = 0.01] for rearing behavior (Fig 6F), where males displayed more frequent rearing episodes than females. No significant effects of genotype or treatment were detected for rearing behavior.

## Discussion

The primary objective of this study was to evaluate the impact of chronic CBD (3 mg/kg) administration within an aged (15−18 months) cohort of HIV-1 Tat transgenic mice. Utilizing a longitudinal experimental design, we integrated behavioral assays with biochemical quantification to assess changes in object recognition, anxiety-like behaviors, spontaneous nociception, and eCB signaling. While previous research has largely focused on acute or short-term CBD exposure [51,90,91], this framework employed a 12-week treatment protocol to specifically target four CNS regions essential for modulating cognition, affect, and pain: the PFC, amygdala, spinal cord, and brainstem. Furthermore, by incorporating sex as a biological variable and examining multiple eCB ligands and related lipids (AEA, 2-AG, and AA) alongside their metabolic enzymes (FAAH, MAGL) and receptors (CB_1_R, CB_2_R, GPR55), this work provides a comprehensive map of how prolonged CBD intervention interacts with an aging neuroHIV mouse model.

In the present study, chronic CBD administration effectively mitigated cognitive deficits and restructured the biochemical landscape in a manner that is dependent on both sex and Tat expression. The NOR task demonstrated that while Tat exposure significantly impaired recognition memory, a finding consistent with established literature [67,68,92,93], chronic CBD treatment enhanced the preference for the novel object as compared to the familiar object across genotypes. Notably, these beneficial effects were particularly pronounced in female mice, suggesting a sex-specific sensitivity to cannabinoid-mediated cognitive recovery in the context of neuroHIV. While numerous preclinical investigations have established that CBD can restore memory function across various neuropathological models [5–9], a previous study from our laboratory [51] found that acute CBD administration failed to improve recognition memory in Tat transgenic mice. This discrepancy strongly suggests that the neuroprotective benefits of CBD in the PFC require a chronic dosing regimen to achieve the necessary steady-state concentrations or to induce long-term neuroplastic changes that acute interventions cannot trigger. While not directly evaluated in this study, literature suggests these cognitive enhancements may be supported by CBD’s established antioxidant and anti-inflammatory pathways [94–97], which could counter the age- and viral-induced oxidative stress known to damage memory processing [94–96]. Furthermore, anti-inflammatory profile of CBD likely plays a fundamental role in restoring cognitive function. Previous research has demonstrated that CBD inhibits the elevation of proinflammatory cytokines, such as TNF-α and IL-6, within the PFC and hippocampus in models of cerebral malaria [97] and hepatic encephalopathy [98,99]. These effects may potentially be mediated through the 5-HT_1A_ [99] and A2A adenosine receptors [98], which may facilitate a reduction in neuroinflammation and a subsequent recovery of synaptic signaling.

It is further suggested by prior literature that CBD can improve cognitive performance by modulating cerebral blood flow in regions critical for memory [90]. Clinical evidence in healthy subjects has shown that acute CBD (600 mg) significantly increased blood flow to the hippocampus, which correlated with reduced reaction times in working memory tasks [90]. While the present study did not look at blood flow, the behavioral improvements appear to be linked to a region-specific restructuring of the eCB system which may be heavily influenced by biological sex. Importantly, in the PFC we observed that chronic CBD treatment induced only subtle, nuanced changes in eCB ligands, receptors, and metabolic enzymes rather than a global overhaul of the system [91]. Our results demonstrate that CBD’s cognitive rescue was specifically pronounced in females, a finding that correlates with the normalization of FAAH expression. Although the present study did not show Tat-induced upregulation of AEA, the Tat-induced upregulation of FAAH in the PFC likely creates a “local endocannabinoid deficiency” by accelerating the degradation of AEA [100,101]. By abolishing this genotype-driven FAAH elevation, chronic CBD may have stabilized AEA tone to facilitate the restoration of recognition memory. Furthermore, while males exhibited higher baseline CB_1_R, CB_2_R, and GPR55 levels in the PFC, CBD’s ability to decrease AA levels specifically in Tat(+) mice suggests an anti-inflammatory effect that may be more critical for cognitive recovery in the female phenotype [102,103].

While most literature suggests that the Tat protein significantly increases anxiety-like behavior [104–108], the present results demonstrate a paradoxical effect. In the EMP task, Tat(+) mice exhibited increased total distance traveled, higher average speeds, and a greater number of open arm entries compared to Tat(–) controls. This divergence likely stems from the use of an aged cohort (15–18 months) in the present study, as Tat has been shown to modulate cognition and behavior differently across ages [106,109]. In senescent models, advanced neurodegeneration may manifest as behavioral disinhibition or apathy rather than the hyper-vigilance typically observed in young adults [109]. Furthermore, the duration of Tat exposure must be considered; prolonged, low-level expression over 3 months may trigger compensatory adaptations within the amygdala that are absent in acute or young-adult models [104]. Regarding the therapeutic intervention, the absence of CBD-induced anxiolytic effects stands in contrast to several previous reports [43,44,110–114]. This lack of efficacy may be attributed to the well-documented biphasic dose-response curve of CBD [115,116]. The dosage utilized here (3 mg/kg) may fall below the therapeutic threshold required to elicit affective changes in the CNS of an aged cohort. Additionally, the route of administration likely played a critical role. While previous studies utilized either intraperitoneal [44,112–114,117] or intracerebroventricular [43,118] routes which produce rapid peak plasma concentrations, the s.c. route used here results in slower absorption. In aged mice, who exhibit reduced metabolic clearance [119], chronic s.c. administration over 12 weeks may have led to a steady-state accumulation of CBD, potentially inducing pharmacological tolerance or the desensitization of downstream signaling pathways.

Finally, the failure of chronic CBD to attenuate anxiety-like behavior may be a consequence of age-related decline in the serotonergic system. While CBD’s anti-anxiety profiles are frequently mediated by 5-HT_1A_ receptor agonism [43,44], aging significantly reduces 5-HT_1A_ receptor density and binding affinity within the CNS [120–122]. This diminished receptor population could render such pathways less responsive to CBD, even as distinct eCB targets successfully modulated cognitive outcomes. In the amygdala, the biochemical landscape may help explain the “behavioral disinhibition” and increased locomotor activity observed in males and Tat(+) mice. While Tat(+) mice exhibited elevated 2-AG and AA levels, female mice also maintained higher baseline 2-AG and MAGL expression compared to males, contrasting with prior studies that indicate females generally have lower eCB levels [123,124]. This difference may arise due to the presence of Tat, as previous studies demonstrate that Tat expression can increase eCB levels [125,126] and suggests that the female amygdala may exhibit a higher eCB turnover rate in the context of neuroHIV. While CBD treatment successfully reduced AA and 2-AG levels in Tat(+) mice, it also significantly lowered AEA levels through a complex sex x genotype x treatment interaction. The dramatic sex-specific sensitivity of CB_1_R, CB_2_R, and GPR55 in this region suggests that while CBD may have shifted the amygdala toward a more homeostatic state, the underlying Tat-mediated damage to the amygdala circuits may be too robust, particularly in males, for CBD to show traditional anxiolytic effect.

Spontaneous nociception was evaluated using the tail-flick and hot-plate assays, reflecting the clinical reality of chronic pain as a primary ailment among PLWH that significantly impairs daily functioning [127–129]. A robust genotype effect was observed, characterized by significantly higher withdrawal latencies in both assays for Tat(+) mice compared to Tat(–) controls. This inherent thermal hypoalgesia aligns with previous preclinical findings in the Tat transgenic mouse model reporting mechanical allodynia and nerve fiber damage [75,130,131]. Mechanistically, Tat is known to induce “leaky” calcium signaling and mitochondrial dysfunction, which lowers the threshold for nociceptive firing in both spinal and supraspinal regions of the CNS [76,132,133]. Notably, a previous study from our laboratory indicated that acute CBD treatment (3, 10, 30 mg/kg) failed to alter thermal latencies [51]. This discrepancy likely arises from the chronic versus acute nature of the administration; acute dosing may be insufficient to remodel pain pathways, whereas 12 weeks of chronic exposure appears to elicit distinct pharmacological shifts. Interestingly, while chronic CBD treatment had no significant effect on the spinal tail-flick reflex, it paradoxically reduced withdrawal latencies in the hot-plate test, indicating increased supraspinal thermal sensitivity. This divergence is characteristic of agents that modulate higher-order brain centers without impacting basic spinal reflexes. The tail-withdrawal response is a purely spinal reflex [134], whereas the hot-plate response is a supraspinal behavior [135] requiring conscious perception and motor coordination mediated by the thalamus and periaqueductal gray [136].

The reduction in hot-plate latency suggests that chronic CBD may have induced supraspinal sensitization to thermal stimuli. This effect may be driven by CBD’s interaction with TRPV1 (vanilloid) receptors [137–139]. Although CBD is often recognized as an analgesic via TRPV1 desensitization [140,141], chronic low-dose exposure particularly in the presence of the Tat protein can lead to receptor sensitization. Tat has been shown to synergistically interact with cannabinoids to increase the excitability of TRPV1-expressing neurons [142]. Although not directly assessed in the present study, it is possible that the s.c. administration utilized here resulted in a steady-state brain concentration that favored TRPV1 sensitization over desensitization, which may potentially manifest as the hyperalgesia observed in the supraspinal mediated hot-plate test while leaving the spinal mediated tail-withdrawal sensitivity, unchanged. Hyposensitivity to thermal stimuli due to senescence has been reported previously [143,144]; however, those effects were observed in both males and females, which is in contrast to the sex-specific pattern seen in the present study. In the spinal cord, males exhibited higher tail-withdrawal latencies (lower sensitivity) alongside higher levels of AA and CB_1_R expression. This suggests a higher baseline “nociceptive set-point” in males that persists into aging. The fact that AEA levels were higher in Tat(+) mice but modulated by a three-way interaction with sex and treatment, indicates that the spinal eCB system’s response to viral proteins is not uniform. Furthermore, the inability of CBD to modulate AEA and 2-AG levels in the spinal cord mirrors the lack of metabolic recovery seen in the brainstem, indicating a shared resistance to CBD-mediated homeostasis in the lower-level CNS. CBD’s increase of CB_2_R expression across both sexes in the spinal cord appears insufficient to overcome the persistent inflammatory markers, explaining why the tail-withdrawal reflex remained unchanged by treatment.

A critical physiological finding in the present study was the observation of chronic CBD-induced hyperthermia. While most literature suggests that CBD alone does not significantly alter body temperature [145–147], our chronic 3 mg/kg s.c. regimen in an aged model resulted in a subtle elevation of baseline body temperature. It is important to note that while chronic CBD induced a statistically significant elevation in body temperature, the difference between vehicle- and CBD-treated groups was only 0.09 °C; this negligible (<0.5%) shift in the physical thermal gradient between the paw and the 55 °C testing surface cannot mathematically account for the robust reduction in hot-plate latencies, confirming a true alteration in supraspinal nociceptive sensitivity. Therefore, we hypothesize that this thermoregulatory shift may involve an imbalance between 5-HT_1A_-mediated cooling and TRPV1-mediated thermogenesis [148,149]. As established, aging significantly declines 5-HT_1A_ receptor density, which may compromise the brain’s primary cooling mechanism [150,151], leaving unmonitored TRPV1 pathways highly sensitive to cannabinoid-driven or Tat-sensitized thermogenic signaling [142] Furthermore, steady-state pharmacokinetic accumulation from chronic s.c. administration in senescent animals with reduced metabolic clearance could continuously drive these metabolic or adipose shifts [152,153], though direct receptor kinetics remain to be verified.

Beyond thermoregulation, a significant genotype effect was observed in general motor activity. While some studies suggest Tat exposure decreases locomotion [79,154–156], our Tat(+) mice exhibited a marked increase in total distance traveled compared to Tat(–) controls. We hypothesize that this hyperactivity may be driven by Tat-mediated dysregulation of the dopaminergic system, specifically by interfering with dopamine transporters to increase synaptic dopamine levels, yielding a psychostimulant-like effect [157–163]. Interestingly, chronic CBD treatment did not modulate this locomotor activity, aligning with past models of hyper- and hypolocomotion [41,113,164]. The lack of effect may stem from CBD’s nature as a partial agonist for D2 dopamine receptors or tolerance, which primarily drive hyperlocomotion, suggesting the current dose was insufficient to override the robust dopaminergic disruption caused by chronic Tat expression [165,166]. However, because direct dopamine kinetics were not quantified in the current study, this mechanism remains speculative and requires future empirical testing. Most strikingly, the brainstem results provide a biochemical basis for the observed hyperthermia and hot plate hyperalgesia. Unlike other regions where males showed higher receptor density, the brainstem GPR55 expression was significantly higher in females. We observed a profound three-way interaction for 2-AG and MAGL, indicating that brainstem lipid metabolism is highly sensitive to the combination of sex and CBD [167,168]. While males exhibited higher baseline AEA and FAAH in this region, the CBD-driven decrease in FAAH and AA in Tat(+) mice suggests a profound “rewiring” of supraspinal thermoregulatory centers [169]. Ultimately, the CBD-driven restructuring of the brainstem eCB system favored metabolic heat production over thermal pain suppression, a physiological shift that occurred in both sexes but may be driven by different receptor densities such as GPR55 in females versus CB_1_R/CB_2_R in males.

While the present study offers a comprehensive mapping of the CBD-Tat interaction in the aging CNS, several limitations should be considered. First, it is important to contextualize the temporal relationship between our dosing regimen and behavioral testing. All assessment were conducted approximately 72 hours after the final chronic CBD treatment. Given the highly lipophilic nature of CBD, prolonged 12-week treatment should cause significant accumulation in lipid-rich compartments, including adipose tissue and the CNS, which can substantially extend the drug’s elimination kinetics via redistribution from tissue depots [170,171]. While the acute plasma half-life of CBD in mice is relatively brief, prolonged chronic dosing regimens yield highly persistent, measurable concentrations of the drug directly in brain tissue [172]. Consequently, it remains unclear whether the reported behavioral and biochemical outcomes reflect the continuous, steady-state presence of residual CBD, permanent long-term neuroplastic adaptations within the eCB system, or a combination of both. Future studies incorporating explicit plasma and brain tissue clearance kinetics at multiple post-dosing intervals will be necessary to fully dissociate the acute physiological levels of CBD from enduring structural neuroplasticity. Second, our biochemical analyses were performed on tissue homogenates, which lack the cellular resolution to determine whether the observed eCB shifts occur specifically within neurons or glia. To address this, future research should utilize *in vivo* calcium imaging [93,173] or eCB GRAB sensors [174,175] to observe real-time neuronal activity and ligand dynamics. This functional approach would help clarify if the “cognitive rescue” observed in females corresponds to specific neural firing patterns during memory tasks. Third, while the inducible Tat transgenic mouse is a robust model for neuroHIV, it lacks other viral proteins such as gp120 and Nef [176] that contribute to HIV-associated neuroinflammation. Since Tat expression in this model is specifically astrocyte-driven, it may not fully replicate the complex multi-cellular interplay seen in PLWH. Future studies employing models like EcoHIV [177,178] could better capture the synergistic effects of the full viral proteome. Fourth, while utilizing aged mice (15–18 months) is a significant strength for modeling the aging population of PLWH, senescent animals exhibit inherent age-related physical declines that can confound behavioral data [179]. Fifth, it is important to note that the current study focused exclusively on behavioral outcomes and the molecular profiling of the eCB system. Therefore, future studies integrating high-throughput eCB ligand analysis with localized cytokine profiling will be essential to fully elucidate the reciprocal relationship between CBD-induced eCB modulation and the attenuation of neuroinflammation in the Tat model. Sixth, although the study was sufficiently powered to detect major effects, some regional molecular shifts achieved significance with power below 80%. Future investigations with expanded sample sizes should be used to further confirm the strength of these specific three-way interactions. Finally, our findings highlight a “tipping point” in the brainstem where chronic CBD transitioned from being neuroprotective to inducing physiological sensitivity. This suggests that a broader dose-response curve is necessary in future investigations to identify the precise therapeutic window that provides cognitive benefits without exacerbating thermal sensitivity or hyperthermia. By moving toward live-cell imaging and more complex viral models, we can further refine the safety and efficacy of cannabinoid therapies for the aging brain.

## Materials and methods

All experiments described here were approved by the University of North Carolina at Chapel Hill Institutional Animal Care and Use Committees (IACUC Protocol No. 23.056) and conducted following the National Institutes of Health Guide for the Care and Use of Laboratory Animals (NIH Publication No. 85−23).

### Animals

Doxycycline (DOX)-inducible, brain-restricted HIV-1_IIIB_ Tat_1–86_ transgenic mice were generated on a hybrid C57BL/6J background, as previously described [19,20]. DOX-induced Tat expression in the brain has been validated using both DOX chow [19,20] and DOX intraperitoneal injection models [67]. Tat expression is driven by a tetracycline-responsive promoter regulated by glial fibrillary acidic protein (GFAP) and induced through a specially formulated chow containing 6 mg/g DOX (product TD.09282; Inotiv, Indianapolis, IN). Genotyping at four weeks of age identified Tat(+) and Tat(−) mice. Tat(+) mice express both the GFAP-rtTA and TRE-tat genes, while Tat(−) control mice express only the GFAP-rtTA gene. For this study, aged mice (*N* = 62, 31 females) 15–18 months were used. Both Tat(−) and Tat(+) mice were maintained on ad libitum DOX chow for one month before undergoing chronic CBD injections which was followed by behavioral experiments. Mice were housed on a reversed 12-hour light/dark cycle. After behavioral experiments, animals were deeply anesthetized with isoflurane, and anesthesia depth was confirmed using the toe-pinch response. Unresponsive animals were decapitated, and brain and spinal cord tissues were collected for further analysis.

### Drug treatment

For behavioral experiments, animals were chronically injected subcutaneously (s.c.) for 3 months with either vehicle [1:1:18; ethanol, Kolliphor, 0.9% saline] or 3 mg/kg CBD (Cat#90080, Cayman Chemical, Ann Arbor, MI) dissolved in vehicle. CBD dose was chosen as it has shown to improve cognition in rodents [21,23,51] and in healthy aged participants [180,181] without adverse effects [22]. Chronic vehicle or CBD treatments were randomized in all the experiments.

### Experimental design

The experimental timeline is presented in Fig 1. Male and female Tat(−) and Tat(+) mice (15−18 months of age) were administered DOX chow for four weeks starting at week 1 to induce Tat expression. From week 4, mice received chronic s.c. injections of either vehicle or CBD (3 mg/kg). Injections were continued once a day / 5 days a week (Mon – Fri) for 12 consecutive weeks (weeks 4−15). Body mass was recorded at week 4, prior to initiation of injections, and again at the conclusion of treatment (week 15). Behavioral assessments were conducted following the completion of the injection regimen. At week 16 (at least 72 hours after the final chronic drug injection), body temperature, tail withdrawal latency, and hot plate tests were performed and at week 17, locomotor activity, elevated plus maze, and novel object recognition tasks were conducted. At the conclusion of behavioral testing, mice were anesthetized and sacrificed by rapid decapitation. Brains were immediately extracted, and the prefrontal cortex, amygdala, brainstem, and spinal cord were dissected, snap-frozen, and stored at −80 °C until further processing. The right hemisphere was allocated for mass spectrometry analysis, while the left hemisphere was used for western blot analysis.

### Behavioral experiments

#### Body mass.

To monitor health, animals were weighed regularly. Mice were gently picked up and placed in a transparent container positioned on a portable scale balance (Fisher Scientific, Cat# S94793A, Waltham, MA). Once body mass was recorded, the mice were promptly returned to their home cages.

### Spontaneous heat-evoked nociception

The tail-withdrawal and hot-plate assays were used to assess spontaneous heat-evoked nociception, targeting spinal and supraspinal pathways, respectively [24]. In the tail-withdrawal test, the distal one-third of each mouse’s tail was submerged in a water bath maintained at 56°C ± 0.1°C (Thermo Scientific, Precision General-Purpose Water Bath, Model 181, Waltham, MA). The latency to withdraw the tail was recorded as a measure of nociception, with a maximum cut-off time of 10 seconds to prevent tissue damage. Immediately after the tail-withdrawal test, the hot-plate assay was performed. Mice were placed on a heated surface (55°C ± 0.1°C; IITC, Inc., MOD 39, Woodland Hills, CA) enclosed within a Plexiglas™ chamber (15 cm height, 10 cm diameter) to prevent escape. Nociceptive responses, such as paw withdrawal, paw licking, or jumping, were used to determine the time spent on the hot plate. To prevent tissue damage, a maximum cut-off time of 15 seconds was enforced.

### Locomotor activity

Spontaneous motor activity was assessed using an open field activity chamber (SD Instruments, Photobeam Activity System–Open Field, CA, USA). Mice were habituated to the chamber 24 hours before testing to minimize stress-related effects. The chamber consisted of a 16 cm x 16 cm Plexiglas™ enclosure equipped with 16 photo-beam sensors along both the x- and y-axes, connected to a computer console for precise recording of ambulatory movements. On the test day, locomotor activity was recorded over a 10-minute period, capturing the ambulatory movements of each mouse within the enclosure. Both the habituation and testing sessions were conducted in a dark room to reduce external stimuli and ensure consistency.

### Novel object recognition

The novel object recognition (NOR) task is a well-established method for assessing object recognition memory, based on the natural tendency of mice to explore new objects or stimuli [182,183]. The NOR task was conducted in the open field activity chamber (SD Instruments, Photobeam Activity System–Open Field, CA, USA), which was also used for the locomotor activity task. The NOR task consisted of three phases: habituation, training, and testing, as previously described [183]. In the training phase, two identical (familiar) objects were placed in the arena, and mice were allowed to explore for 10 minutes. The testing phase began 60 minutes after training in which one familiar object was replaced (randomized for each mouse) with a new (novel) object, and mice were given 10 minutes to explore the arena. Photo-beam breaks recorded the time spent exploring the familiar and novel objects during the session. Since mice naturally prefer novel stimuli, their preference for the novel object was used to quantify object recognition memory. The objects used are equal in size but differ in shape and color and held no natural significance for the mice. Total exploration time for both objects was calculated, and a discrimination index (DI) was computed as: [(time spent exploring the novel object) – (time spent exploring the familiar object)] / total time spent exploring both objects. A DI of 0 indicates no preference, 1 indicates complete preference for the novel object, and −1 indicates complete preference for the familiar object.

### Elevated plus maze

The elevated plus maze (EPM) is a commonly used test to assess anxiety-like behavior in rodents, with increased anxiety typically indicated by a stronger preference for the enclosed, darker arms of the maze [184]. The maze consists of a plus-shaped platform elevated 38 cm above the ground, featuring two open arms (without walls) and two closed arms, each 30 cm long and 5 cm wide. The closed arms are surrounded by 15 cm tall beige walls, while the open arms remain unprotected. The maze was positioned under indirect lighting to ensure consistent illumination across trials. Mice were placed in the center of the maze and allowed to explore freely for 10 minutes. Their behavior was recorded via a video camera (GoPro Hero 6 Black, v2.10, CA, USA) mounted above the maze. Anxiety levels were measured by the proportion of time spent in the open arms, with results are expressed as a percentage of total exploration time.

### Endocannabinoids and related lipids analysis

Endogenous cannabinoid ligands, including the primary endocannabinoids *N*-arachidonoylethanolamine (AEA) and 2-arachidonoylglycerol (2-AG), as well as the minor endocannabinoids *N*-oleoylethanolamide (OEA), *N*-palmitoylethanolamide (PEA), and arachidonic acid (AA), were quantified using ultraperformance liquid chromatography-tandem mass spectrometry (UPLC-MS/MS) in CNS regions including prefrontal cortex, amygdala, brainstem and spinal cord, from both female and male Tat transgenic mice. The extraction and quantification of endocannabinoids and related lipids were performed as outlined previously [69,77,185].

### Western blot analysis

CNS regions, including the prefrontal cortex, amygdala, brainstem and spinal cord from female and male Tat transgenic mice, were analyzed for cannabinoid and cannabinoid-like receptor protein expression (CB_1_R, CB_2_R, and GPR55), and endocannabinoid-degrading enzymes (FAAH and MAGL). Tissue samples from the left hemisphere were homogenized on ice in an appropriate volume of ice-cold Pierce™ RIPA lysis and extraction buffer, supplemented with Halt™ phosphatase and protease inhibitor cocktail. After homogenization, samples were centrifuged at 10,000 g for 10 minutes at 4°C. The protein concentration of the tissue lysates was measured using the Pierce™ BCA protein assay kit. Protein lysates were then mixed with NuPAGE™ LDS sample buffer and XT Reducing Agent in a 1:2.5 ratio and denatured at 85°C for 10 minutes. Equal amounts of protein (20 μg per lane) were resolved on 10% Bis-Tris Criterion™ XT precast gels using XT MOPS running buffer at 120 volts for 1.5 hours in a Criterion™ vertical electrophoresis cell. The proteins were transferred from the gel onto nitrocellulose membranes in 10x Tris/Glycine buffer at 1–4°C and 100 volts for 1 hour using a Criterion™ blotter. After transfer, the blots were rinsed with phosphate-buffered saline (PBS) and blocked with Intercept^®^ blocking buffer at room temperature for 1 hour. The membranes were then incubated overnight at 4°C with primary antibodies against anti-CB_1_R (rabbit polyclonal; Proteintech, Cat# 17978–1-AP, 1:1000 dilution), anti-CB_2_R (rabbit polyclonal; AbClonal, Cat# A1762, 1:1000 dilution), anti-GPR55 (rabbit polyclonal; ABclonal, Cat# A12890, 1:1,000 dilution), anti-FAAH (mouse monoclonal; Abcam, Cat# ab54615, 1:1000 dilution), and anti-MAGL (rabbit polyclonal; Abcam, Cat# ab24701, 1:1000 dilution). Anti-GAPDH antibody (mouse monoclonal; Abcam, Cat# ab125247, 1:15,000 dilution) was used as a loading control. After primary antibody incubation, the blots were washed three times with PBS containing 0.1% Tween-20 (PBST) and then incubated with IRDye® 680RD Donkey anti-Mouse IgG (LI-COR Biosciences, Cat# 926–68072, 1:15,000 dilution) and IRDye® 800CW Donkey anti-Rabbit IgG (LI-COR Biosciences, Cat# 925–32213, 1:15,000 dilution) secondary antibodies for 1 hour at room temperature in Intercept^®^ blocking buffer with 0.2% Tween-20 and 0.01% SDS. Following another set of washes with PBST, protein bands were detected using the Odyssey^®^ CLx infrared imaging system (LI-COR Biosciences) and analyzed using Empiria Studio® software (version 2.3.0). Data presented are normalized to the housekeeping gene GAPDH.

### Statistical analysis

Data are presented as mean ± standard error of the mean (SEM). Body mass was analyzed using a four-way mixed ANOVA between acute and chronic drug treatment with time (2 levels: week 4, week 15) as a within-subjects factor, and sex (2 levels: females and males), genotype [2 levels: Tat(–) and Tat(+)], and treatment (2 levels: vehicle, CBD) as between-subjects factors. Spontaneous nociception (tail withdrawal and hot plate assay), behavioral experiments (locomotor activity, elevated plus maze, novel object recognition), eCB levels (AEA, 2-AG, and AA) and protein quantification (CB_1_R, CB_2_R, GPR55, FAAH, and MAGL) were analyzed by three-way ANOVAs with sex, genotype, and treatment as between-subjects factors. Main or interaction effects were followed by Tukey’s post hoc tests where applicable. An alpha level of *p* ≤ 0.05 was considered significant for all statistical tests. To assess the statistical reliability of the reported three-way interactions (sex x genotype x treatment), a post-hoc power analysis was conducted using G*Power 3.1. Achieved power for these interactions is reported in Supplemental S1–S3 File. SPSS Statistics 31 (IBM, Chicago, IL) and Prism GraphPad 10.6.1 (San Diego, CA) were used for data analysis and graphing, respectively.

## Supporting information

S1_File Three-way ANOVA showing the effect of chronic CBD on behavior in Tat tg mice. (PDF)

S2_File Three-way ANOVA showing the effect of chronic CBD on eCB levels in Tat tg mice. (PDF)

S3_File Three-way ANOVA showing the effect of chronic CBD on CB_1_R, CB_2_R, and GPR55 levels in Tat tg mice. (PDF)

S4_File Two-way ANOVA showing CBD and its metabolites in cortex and plasma. (PDF)

S1_Fig CBD and CBD-COOH metabolite in the cortex and plasma. (A) Levels of CBD (ng/mL) in (A) cortex and (B) plasma. Levels of CBD-COOH (ng/mL) in plasma (C). Note: CBD-COOH levels in the cortex were not detected. Data represented as mean ± SEM. Statistical significance was assessed by overall ANOVAs, ^#^*p* < 0.05 main effect of genotype, ^ψ^*p* < 0.05 main effect of sex, ^α^*p* < 0.05 sex x genotype interaction. CBD dose = 3 mg/kg. *N* = 30(16f).
(TIF)

S2_Fig Endocannabinoid and AA levels in the hippocampus. (A) AEA, (B) 2-AG, and (C) AA levels in the hippocampus. (D) Representative Western blot bands for CB_1_R, CB_2_R, GPR55, FAAH, MAGL, and loading control GAPDH. (E) CB_1_R (F) CB_2_R, (G) GPR55, (H) FAAH, and (I) MAGL protein expression in the hippocampus. Data represented as mean ± SEM. Statistical significance was assessed by overall ANOVAs, ^#^*p* < 0.05 main effect of genotype, ^ψ^*p* < 0.05 main effect of sex, ^δ^*p* < 0.05 main effect of treatment, ^α^*p* < 0.05 sex x genotype interaction, ^β^*p* < 0.05 sex x treatment interaction, ^χ^*p* < 0.05 genotype x treatment interaction, ^ε^*p* < 0.05 sex x genotype x treatment interaction. CBD dose = 3 mg/kg. *N* = 62(31f) for eCB data and *N* = 59(29f) for western blot data.
(TIF)

S3_Fig Related eCB lipids levels in CNS regions. PEA and OEA levels in (A) PFC, (B) hippocampus, (C) amygdala, (D) brainstem, and (E) spinal cord. Data represented as mean ± SEM. Statistical significance was assessed by overall ANOVAs, ^#^*p* < 0.05 main effect of genotype, ^ψ^*p* < 0.05 main effect of sex, ^δ^*p* < 0.05 main effect of treatment. A four-way mixed ANOVA for body mass; ^τ^*p* < 0.05 main effect of time, ^σ^*p* < 0.05 main time x sex x treatment interaction and ^γ^*p* < 0.05 main time x genotype x treatment interaction. CBD dose = 3 mg/kg. *N* = 62(31f).
(TIF)

S4_Fig Original and unedited western blots for the prefrontal cortex. Images show (A) CB_1_R, MAGL, and GAPDH, (B) CB_2_R, FAAH, and GAPDH, and (C) GPR55 for female Tat(–), male Tat(–), female Tat(+), and male Tat(+) mice. C: positive control; M: molecular weights of marker protein (kDa).
(TIF)

S5_Fig Original and unedited western blots for the hippocampus. Images show (A) CB_1_R, MAGL, and GAPDH, (B) CB_2_R, FAAH, and GAPDH, and (C) GPR55 for female Tat(–), male Tat(–), female Tat(+), and male Tat(+) mice. M: molecular weights of marker protein (kDa). Note, 2 samples in female Tat(+) group and 1 sample in male Tat(+) group were lost during harvest.
(TIF)

S6_Fig Original and unedited western blots for the amygdala. Images show (A) CB_1_R, MAGL, and GAPDH, (B) CB_2_R, FAAH, and GAPDH, and (C) GPR55 for female Tat(–), male Tat(–), female Tat(+), and male Tat(+) mice. M: molecular weights of marker protein (kDa).
(TIF)

S7_Fig Original and unedited western blots for the spinal cord. Images show (A) CB_1_R, MAGL, and GAPDH, (B) CB_2_R, FAAH, and GAPDH, and (C) GPR55 for female Tat(–), male Tat(–), female Tat(+), and male Tat(+) mice. M: molecular weights of marker protein (kDa).
(TIF)

S8_Fig Original and unedited western blots for the brainstem. Images show (A) CB_1_R, MAGL, and GAPDH, (B) CB_2_R, FAAH, and GAPDH, and (C) GPR55 for female Tat(–), male Tat(–), female Tat(+), and male Tat(+) mice. M: molecular weights of marker protein (kDa).
(TIF)
