# Supplementary material for: Chronic CBD treatment differentially modulates neurobehavioral outcomes and endocannabinoid signaling in an aged HIV-1 Tat transgenic mouse model
Source: PLoS One. 2026 Jul 20;21(7):e0353267. doi: 10.1371/journal.pone.0353267 (PMC13384326; doi:10.1371/journal.pone.0353267)
Supplement: S1_File — (PDF) [file pone.0353267.s001.pdf]

Supplemental Information

S1_Table: Three-way ANOVA showing the effect of chronic CBD on behavior in Tat tg mice.

Behavior	Sex effect *F, p*	Genotype effect *F, p*	Treatment effect *F, p*	Sex x Genotype *F, p*	Sex x Treatment *F, p*	Genotype x Treatment *F, p*	Sex x Genotype x Treatment *F, p* (post-hoc power analysis)
Body mass (g) [Before CBD treatment]	$F(1,54) = 0.38$<br>$p = 0.53$	$F(1,54) = 2.90$<br>$p = 0.09$	*NA*	$F(1,54) = 1.87$<br>$p = 0.17$	*NA*	*NA*	*NA*
Body mass (g) [After first CBD treatment]	$F(1,54) = 1.14$<br>$p = 0.28$	$F(1,54) = 3.30$<br>$p = 0.07$	$F(1,54) = 0.82$<br>$p = 0.36$	$F(1,54) = 1.16$<br>$p = 0.28$	$F(1,54) = 4.22$<br>**$p = 0.04$**	$F(1,54) = 0.008$<br>$p = 0.92$	$F(1,54) = 1.91$<br>$p = 0.17$<br>(30%)
Body mass (g) [After last CBD treatment]	$F(1,54) = 4.55$<br>**$p = 0.03$**<br>**F < M**	$F(1,54) = 1.90$<br>$p = 0.17$	$F(1,54) = 0.63$<br>$p = 0.17$	$F(1,54) = 1.90$<br>$p = 0.17$	$F(1,54) = 1.61$<br>$p = 0.21$	$F(1,54) = 1.31$<br>$p = 0.25$	$F(1,54) = 1.72$<br>$p = 0.19$<br>(39%)
Body temperature (°C)	$F(1,54) = 1.55$<br>$p = 0.21$	$F(1,54) = 2.79$<br>$p = 0.10$	$F(1,54) = 8.68$<br>**$p = 0.005$**<br>**Vehicle < CBD**	$F(1,54) = 0.48$<br>$p = 0.49$	$F(1,54) = 0.33$<br>$p = 0.56$	$F(1,54) = 1.00$<br>$p = 0.32$	$F(1,54) = 0.001$<br>$p = 0.97$<br>(5%)
Tail withdrawal latency (s)	$F(1,54) = 13.12$<br>**$p < 0.001$**<br>**F < M**	$F(1,54) = 7.42$<br>**$p = 0.009$**<br>**Tat(–) < Tat(+)**	$F(1,54) = 0.13$<br>$p = 0.71$	$F(1,54) = 6.32$<br>**$p = 0.01$**	$F(1,54) = 1.17$<br>$p = 0.28$	$F(1,54) = 0.10$<br>$p = 0.74$	$F(1,54) = 0.04$<br>$p = 0.82$<br>(5%)
Hot plate latency (s)	$F(1,54) = 0.02$<br>$p = 0.86$	$F(1,54) = 7.37$<br>**$p = 0.009$**<br>**Tat(–) < Tat(+)**	$F(1,54) = 5.36$<br>**$p = 0.02$**<br>**Vehicle > CBD**	$F(1,54) = 0.26$<br>$p = 0.60$	$F(1,54) = 0.75$<br>$p = 0.38$	$F(1,54) = 2.27$<br>$p = 0.13$	$F(1,54) = 0.29$<br>$p = 0.58$<br>(8%)
LA: Total activity	$F(1,54) = 9.87$<br>**$p = 0.003$**<br>**F < M**	$F(1,54) = 4.45$<br>**$p = 0.03$**<br>**Tat(–) < Tat(+)**	$F(1,54) = 0.54$<br>$p = 0.46$	$F(1,54) = 0.22$<br>$p = 0.63$	$F(1,54) = 0.19$<br>$p = 0.66$	$F(1,54) = 0.01$<br>$p = 0.91$	$F(1,54) = 0.001$<br>$p = 0.97$<br>(5%)
LA: Rearing	$F(1,54) = 5.84$<br>**$p = 0.01$**<br>**F < M**	$F(1,54) = 1.51$<br>$p = 0.22$<br>**Tat(–) < Tat(+)**	$F(1,54) = 0.21$<br>$p = 0.64$	$F(1,54) = 1.31$<br>$p = 0.25$	$F(1,54) = 0.37$<br>$p = 0.54$	$F(1,54) = 1.83$<br>$p = 0.18$	$F(1,54) = 0.03$<br>$p = 0.85$<br>(5%)

NOR: Total object exploration time (s)	$F(1,54) = 0.12$ $p = 0.72$	$F(1,54) = 1.19$ $p = 0.27$	$F(1,54) = 0.53$ $p = 0.46$	$F(1,54) = 0.41$ $p = 0.52$	$F(1,54) = 0.38$ $p = 0.53$	$F(1,54) = 6.94$ **$p = 0.01$**	$F(1,54) = 2.70$ $p = 0.10$ (41%)
NOR: Discrimination index	$F(1,54) = 3.82$ $p = 0.06$	$F(1,54) = 16.15$ **$p < 0.001$** **Tat(–) > Tat(+)**	$F(1,54) = 10.83$ **$p = 0.002$** **Vehicle < CBD**	$F(1,54) = 0.07$ $p = 0.79$	$F(1,54) = 8.00$ **$p = 0.007$**	$F(1,54) = 0.02$ $p = 0.87$	$F(1,54) = 0.08$ $p = 0.77$ (6%)
EPM: Total distance (m)	$F(1,54) = 4.24$ **$p = 0.04$** **F < M**	$F(1,54) = 3.91$ **$p = 0.05$** **Tat(–) < Tat(+)**	$F(1,54) = 0.01$ $p = 0.92$	$F(1,54) = 0.17$ $p = 0.67$	$F(1,54) = 0.13$ $p = 0.71$	$F(1,54) = 0.42$ $p = 0.51$	$F(1,54) = 0.72$ $p = 0.39$ (14%)
EPM: Average speed (m/s)	$F(1,54) = 4.33$ **$p = 0.04$** **F < M**	$F(1,54) = 3.96$ **$p = 0.05$** **Tat(–) < Tat(+)**	$F(1,54) = 0.05$ $p = 0.80$	$F(1,54) = 0.24$ $p = 0.62$	$F(1,54) = 0.16$ $p = 0.68$	$F(1,54) = 0.44$ $p = 0.51$	$F(1,54) = 0.75$ $p = 0.38$ (15%)
EPM: Open arm entries	$F(1,54) = 0.59$ $p = 0.44$	$F(1,54) = 7.97$ **$p = 0.007$** **Tat(–) < Tat(+)**	$F(1,54) = 0.06$ $p = 0.80$	$F(1,54) = 1.13$ $p = 0.29$	$F(1,54) = 2.02$ $p = 0.16$	$F(1,54) = 0.59$ $p = 0.44$	$F(1,54) = 0.11$ $p = 0.73$ (6%)
EPM: Open arm distance (m)	$F(1,54) = 0.08$ $p = 0.77$	$F(1,54) = 0.01$ $p = 0.92$	$F(1,54) = 0.29$ $p = 0.59$	$F(1,54) = 4.03$ **$p = 0.04$**	$F(1,54) = 2.82$ $p = 0.09$	$F(1,54) = 1.6$ $p = 0.20$	$F(1,54) = 0.08$ $p = 0.77$ (5%)

LA, locomotor activity; NOR, novel object recognition; EPM, elevated plus maze.
