# Supplementary material for: Chronic CBD treatment differentially modulates neurobehavioral outcomes and endocannabinoid signaling in an aged HIV-1 Tat transgenic mouse model
Source: PLoS One. 2026 Jul 20;21(7):e0353267. doi: 10.1371/journal.pone.0353267 (PMC13384326; doi:10.1371/journal.pone.0353267)
Supplement: S2_File — (PDF) [file pone.0353267.s002.pdf]

S2_Table: Three-way ANOVA showing the effect of chronic CBD on eCB levels in Tat tg mice.

Brain region	Ligand	Sex effect *F, p*	Genotype effect *F, p*	Treatment effect *F, p*	Sex x Genotype *F, p*	Sex x Treatment *F, p*	Genotype x Treatment *F, p*	Sex x Genotype x Treatment *F, p*
PFC	AEA	*F*(1,54) = 1.49 *p* = 0.22	*F*(1,54) = 0.97 *p* = 0.32	*F*(1,54) = 2.70 *p* = 0.10	*F*(1,54) = 3.71 *p* = 0.06	*F*(1,54) = 0.14 *p* = 0.70	*F*(1,54) = 0.82 *p* = 0.36	*F*(1,54) = 0.81 *p* = 0.77 (6%)
	2-AG	*F*(1,54) = 0.23 *p* = 0.62	*F*(1,54) = 1.63 *p* = 0.20	*F*(1,54) = 3.78 *p* = 0.06	*F*(1,54) = 3.66 *p* = 0.06	*F*(1,54) = 3.09 *p* = 0.08	*F*(1,54) = 0.004 *p* = 0.94	*F*(1,54) = 11.84 ***p* = 0.001** (95%)
	PEA	*F*(1,54) = 3.02 *p* = 0.08	*F*(1,54) = 0.05 *p* = 0.80	*F*(1,54) = 0.27 *p* = 0.60	*F*(1,54) = 2.29 *p* = 0.13	*F*(1,54) = 0.65 *p* = 0.42	*F*(1,54) = 1.65 *p* = 0.20	*F*(1,54) = 52.6 ***p* < 0.001** (100%)
	OEA	*F*(1,54) = 3.76 *p* = 0.06	*F*(1,54) = 0.16 *p* = 0.69	*F*(1,54) = 0.16 *p* = 0.68	*F*(1,54) = 3.83 *p* = 0.06	*F*(1,54) = 0.97 *p* = 0.32	*F*(1,54) = 0.007 *p* = 0.93	*F*(1,54) = 51.5 ***p* < 0.001** (100%)
	AA	*F*(1,54) = 0.24 *p* = 0.62	*F*(1,54) = 0.79 *p* = 0.37	*F*(1,54) = 1.18 *p* = 0.28	*F*(1,54) = 3.46 *p* = 0.06	*F*(1,54) = 4.68 ***p* = 0.03**	*F*(1,54) = 10.4 ***p* = 0.002**	*F*(1,54) = 1.22 *p* = 0.27 (21%)
Hip	AEA	*F*(1,54) = 18.51 ***p* < 0.001** **F < M**	*F*(1,54) = 0.41 *p* = 0.52	*F*(1,54) = 6.04 ***p* = 0.01** **Vehicle < CBD**	*F*(1,54) = 0.62 *p* = 0.43	*F*(1,54) = 2.95 *p* = 0.09	*F*(1,54) = 1.73 *p* = 0.19	*F*(1,54) = 3.25 *p* = 0.07 (47%)
	2-AG	*F*(1,54) = 1.91 *p* = 0.17	*F*(1,54) = 0.15 *p* = 0.69	*F*(1,54) = 2.15 *p* = 0.14	*F*(1,54) = 0.73 *p* = 0.39	*F*(1,54) = 0.19 *p* = 0.66	*F*(1,54) = 1.44 *p* = 0.23	*F*(1,54) = 31.88 ***p* < 0.001** (99%)
	PEA	*F*(1,54) = 6.62 ***p* = 0.01** **F < M**	*F*(1,54) = 5.62 ***p* = 0.02** **Tat(–) < Tat(+)**	*F*(1,54) = 4.01 ***p* = 0.05** **Vehicle < CBD**	*F*(1,54) = 6.09 ***p* = 0.01**	*F*(1,54) = 0.007 *p* = 0.934	*F*(1,54) = 0.18 *p* = 0.67	*F*(1,54) = 50.09 ***p* < 0.001** (100%)
	OEA	*F*(1,54) = 7.74 ***p* = 0.007** **F < M**	*F*(1,54) = 4.36 ***p* = 0.04** **Tat(–) < Tat(+)**	*F*(1,54) = 0.91 *p* = 0.34	*F*(1,54) = 0.90 *p* = 0.34	*F*(1,54) = 0.22 *p* = 0.63	*F*(1,54) = 0.11 *p* = 0.73	*F*(1,54) = 27.04 ***p* < 0.001** (99%)
	AA	*F*(1,54) = 5.57 ***p* = 0.02** **F < M**	*F*(1,54) = 0.51 *p* = 0.47	*F*(1,54) = 1.41 *p* = 0.24	*F*(1,54) = 0.42 *p* = 0.51	*F*(1,54) = 1.29 *p* = 0.26	*F*(1,54) = 9.32 ***p* = 0.004**	*F*(1,54) = 1.37 *p* = 0.24 (23%)

Amg	AEA	$F(1,54) = 1.81$<br>$p = 0.18$	$F(1,54) = 0.01$<br>$p = 0.90$	$F(1,54) = 4.62$<br>**$p = 0.03$**<br>**Vehicle > CBD**	$F(1,54) = 9.15$<br>**$p = 0.004$**	$F(1,54) = 12.48$<br>**$p < 0.001$**	$F(1,54) = 1.34$<br>$p = 0.25$	$F(1,54) = 4.17$<br>**$p = 0.04$**<br>(57%)
	2-AG	$F(1,54) = 8.09$<br>**$p = 0.006$**<br>**F > M**	$F(1,54) = 14.4$<br>**$p < 0.001$**<br>**Tat(–) < Tat(+)**	$F(1,54) = 0.04$<br>$p = 0.83$	$F(1,54) = 2.83$<br>$p = 0.09$	$F(1,54) = 0.00$<br>$p = 0.99$	$F(1,54) = 5.40$<br>**$p = 0.02$**	$F(1,54) = 2.25$<br>$p = 0.13$<br>(35%)
	PEA	$F(1,54) = 13.4$<br>**$p < 0.001$**<br>**F < M**	$F(1,54) = 2.09$<br>$p = 0.15$	$F(1,54) = 11.2$<br>**$p = 0.001$**<br>**Vehicle > CBD**	$F(1,54) = 19.6$<br>**$p < 0.001$**	$F(1,54) = 0.46$<br>$p = 0.49$	$F(1,54) = 0.10$<br>$p = 0.72$	$F(1,54) = 51.4$<br>**$p < 0.001$**<br>(100%)
	OEA	$F(1,54) = 5.28$<br>**$p = 0.02$**<br>**F < M**	$F(1,54) = 0.75$<br>$p = 0.39$	$F(1,54) = 8.71$<br>**$p = 0.005$**<br>**Vehicle > CBD**	$F(1,54) = 8.10$<br>**$p = 0.006$**	$F(1,54) = 0.33$<br>$p = 0.56$	$F(1,54) = 0.003$<br>$p = 0.95$	$F(1,54) = 32.6$<br>**$p < 0.001$**<br>(99%)
	AA	$F(1,54) = 1.83$<br>$p = 0.18$	$F(1,54) = 0.22$<br>$p = 0.63$	$F(1,54) = 8.44$<br>**$p = 0.005$**<br>**Vehicle > CBD**	$F(1,54) = 1.67$<br>$p = 0.20$	$F(1,54) = 1.68$<br>$p = 0.19$	$F(1,54) = 14.14$<br>**$p < 0.001$**	$F(1,54) = 4.04$<br>**$p = 0.04$**<br>(56%)
BS	AEA	$F(1,54) = 13.80$<br>**$p < 0.001$**<br>**F < M**	$F(1,54) = 0.43$<br>$p = 0.51$	$F(1,54) = 0.11$<br>$p = 0.73$	$F(1,54) = 0.04$<br>$p = 0.83$	$F(1,54) = 2.74$<br>$p = 0.10$	$F(1,54) = 0.09$<br>$p = 0.76$	$F(1,54) = 2.12$<br>$p = 0.15$<br>(33%)
	2-AG	$F(1,54) = 0.03$<br>$p = 0.85$	$F(1,54) = 0.09$<br>$p = 0.75$	$F(1,54) = 1.31$<br>$p = 0.25$	$F(1,54) = 1.27$<br>$p = 0.26$	$F(1,54) = 0.05$<br>$p = 0.82$	$F(1,54) = 0.42$<br>$p = 0.51$	$F(1,54) = 57.1$<br>**$p < 0.001$**<br>(100%)
	PEA	$F(1,54) = 6.51$<br>**$p = 0.01$**<br>**F < M**	$F(1,54) = 5.59$<br>**$p = 0.02$**<br>**Tat(–) > Tat(+)**	$F(1,54) = 3.24$<br>$p = 0.07$	$F(1,54) = 2.54$<br>$p = 0.11$	$F(1,54) = 4.03$<br>**$p = 0.05$**	$F(1,54) = 5.76$<br>**$p = 0.02$**	$F(1,54) = 40.2$<br>**$p < 0.001$**<br>(100%)
	OEA	$F(1,54) = 5.63$<br>**$p = 0.02$**<br>**F < M**	$F(1,54) = 0.81$<br>$p = 0.37$	$F(1,54) = 0.07$<br>$p = 0.78$	$F(1,54) = 0.39$<br>$p = 0.53$	$F(1,54) = 0.55$<br>$p = 0.45$	$F(1,54) = 4.87$<br>**$p = 0.03$**	$F(1,54) = 34.1$<br>**$p < 0.001$**<br>(100%)
	AA	$F(1,54) = 3.31$<br>$p = 0.07$	$F(1,54) = 0.53$<br>$p = 0.46$	$F(1,54) = 0.39$<br>$p = 0.84$	$F(1,54) = 0.25$<br>$p = 0.61$	$F(1,54) = 1.87$<br>$p = 0.17$	$F(1,54) = 11.24$<br>**$p = 0.001$**	$F(1,54) = 3.17$<br>$p = 0.08$<br>(46%)

SC	AEA	$F(1,54) = 1.50$<br>$p = 0.22$	$F(1,54) = 4.83$<br>**$p = 0.03$**<br>**Tat(–) < Tat(+)**	$F(1,54) = 1.43$<br>$p = 0.23$	$F(1,54) = 7.24$<br>**$p = 0.009$**	$F(1,54) = 7.01$<br>**$p = 0.01$**	$F(1,54) = 5.25$<br>**$p = 0.02$**	$F(1,54) = 3.75$<br>$p = 0.06$<br>(6%)
	2-AG	$F(1,54) = 0.86$<br>$p = 0.35$	$F(1,54) = 3.54$<br>$p = 0.06$	$F(1,54) = 3.79$<br>$p = 0.06$	$F(1,54) = 4.55$<br>**$p = 0.03$**	$F(1,54) = 3.56$<br>$p = 0.06$	$F(1,54) = 0.09$<br>$p = 0.75$	$F(1,54) = 38.81$<br>**$p < 0.001$**<br>(99%)
	PEA	$F(1,54) = 9.82$<br>**$p = 0.003$**<br>**F < M**	$F(1,54) = 0.001$<br>$p = 0.97$	$F(1,54) = 0.08$<br>$p = 0.77$	$F(1,54) = 0.42$<br>$p = 0.51$	$F(1,54) = 0.54$<br>$p = 0.46$	$F(1,54) = 7.07$<br>**$p = 0.01$**	$F(1,54) = 15.1$<br>**$p < 0.001$**<br>(98%)
	OEA	$F(1,54) = 13.8$<br>**$p < 0.001$**<br>**F < M**	$F(1,54) = 0.16$<br>$p = 0.68$	$F(1,54) = 0.18$<br>$p = 0.66$	$F(1,54) = 1.03$<br>$p = 0.31$	$F(1,54) = 0.05$<br>$p = 0.81$	$F(1,54) = 9.29$<br>**$p = 0.004$**	$F(1,54) = 17.8$<br>**$p < 0.001$**<br>(95%)
	AA	$F(1,54) = 7.02$<br>**$p = 0.01$**<br>**F < M**	$F(1,54) = 0.01$<br>$p = 0.89$	$F(1,54) = 0.04$<br>$p = 0.83$	$F(1,54) = 2.93$<br>$p = 0.09$	$F(1,54) = 1.05$<br>$p = 0.30$	$F(1,54) = 4.93$<br>**$p = 0.03$**	$F(1,54) = 0.02$<br>$p = 0.87$<br>(5%)

2-AG, 2-arachidonoylglycerol; AEA, *N*-arachidonoylethanolamine; AA, arachidonic acid; Amg, amygdala; BS, brainstem; F, female; M, male; PEA, palmitoylethanolamide; PFC, prefrontal cortex; OEA, oleoylethanolamide; SC, spinal cord.
