# Supplementary material for: Chronic CBD treatment differentially modulates neurobehavioral outcomes and endocannabinoid signaling in an aged HIV-1 Tat transgenic mouse model
Source: PLoS One. 2026 Jul 20;21(7):e0353267. doi: 10.1371/journal.pone.0353267 (PMC13384326; doi:10.1371/journal.pone.0353267)
Supplement: S3_File — (PDF) [file pone.0353267.s003.pdf]

S3_Table: Three-way ANOVA showing the effect of chronic CBD on CB_1R, CB_2R, and GPR55 levels in Tat tg mice.

Brain region	Receptors / Enzymes	Sex effect *F, p*	Genotype effect *F, p*	Treatment effect *F, p*	Sex x Genotype *F, p*	Sex x Treatment *F, p*	Genotype x Treatment *F, p*	Sex x Genotype x Treatment *F, p*
PFC	CB_1R	$F(1,54) = 74.6$ **$p < 0.001$** **F < M**	$F(1,54) = 12.3$ **$p < 0.001$** **Tat(–) > Tat(+)**	$F(1,54) = 0.04$ $p = 0.82$	$F(1,54) = 0.002$ $p = 0.96$	$F(1,54) = 0.73$ $p = 0.39$	$F(1,54) = 2.74$ $p = 0.10$	$F(1,54) = 2.79$ $p = 0.10$ (42%)
	CB_2R	$F(1,54) = 35.7$ **$p < 0.001$** **F < M**	$F(1,54) = 3.47$ $p = 0.06$	$F(1,54) = 0.22$ $p = 0.63$	$F(1,54) = 13.0$ **$p < 0.001$**	$F(1,54) = 0.65$ $p = 0.42$	$F(1,54) = 4.25$ **$p = 0.04$**	$F(1,54) = 1.46$ $p = 0.23$ (24%)
	GPR55	$F(1,54) = 161.1$ **$p < 0.001$** **F < M**	$F(1,54) = 2.60$ $p = 0.11$	$F(1,54) = 0.71$ $p = 0.40$	$F(1,54) = 9.48$ **$p = 0.003$**	$F(1,54) = 0.53$ $p = 0.46$	$F(1,54) = 36.1$ **$p < 0.001$**	$F(1,54) = 19.86$ **$p < 0.001$** (99%)
	FAAH	$F(1,54) = 1.39$ $p = 0.24$	$F(1,54) = 27.20$ **$p < 0.001$** **Tat(–) < Tat(+)**	$F(1,54) = 0.08$ $p = 0.77$	$F(1,54) = 6.66$ **$p = 0.01$**	$F(1,54) = 0.13$ $p = 0.72$	$F(1,54) = 4.83$ **$p = 0.03$**	$F(1,54) = 2.34$ $p = 0.13$ (36%)
	MAGL	$F(1,54) = 10.41$ **$p = 0.002$** **F < M**	$F(1,54) = 0.66$ $p = 0.41$	$F(1,54) = 1.79$ $p = 0.18$	$F(1,54) = 0.98$ $p = 0.32$	$F(1,54) = 0.44$ $p = 0.83$	$F(1,54) = 0.35$ $p = 0.55$	$F(1,54) = 0.76$ $p = 0.38$ (15%)
Hip	CB_1R	$F(1,52) = 120.1$ **$p < 0.001$** **F < M**	$F(1,52) = 0.36$ $p = 0.54$	$F(1,52) = 2.62$ $p = 0.11$	$F(1,52) = 0.82$ $p = 0.36$	$F(1,52) = 4.67$ **$p = 0.03$**	$F(1,52) = 0.62$ $p = 0.43$	$F(1,52) = 0.46$ $p = 0.49$ (11%)
	CB_2R	$F(1,52) = 2.87$ $p = 0.09$	$F(1,52) = 5.99$ **$p = 0.01$**	$F(1,52) = 0.76$ $p = 0.38$	$F(1,52) = 26.$ **$p < 0.001$**	$F(1,52) = 1.61$ $p = 0.20$	$F(1,52) = 0.00$ $p = 0.98$	$F(1,52) = 0.80$ $p = 0.37$ (16%)
	GPR55	$F(1,52) = 3.33$ $p = 0.73$	$F(1,52) = 1.72$ $p = 0.19$	$F(1,52) = 1.92$ $p = 0.17$	$F(1,52) = 1.81$ $p = 0.18$	$F(1,52) = 1.98$ $p = 0.16$	$F(1,52) = 2.33$ $p = 0.13$	$F(1,52) = 2.35$ $p = 0.13$ (37%)

	FAAH	$F(1,52) = 98.5$<br>**$p < 0.001$**	$F(1,52) = 0.63$<br>$p = 0.42$	$F(1,52) = 1.23$<br>$p = 0.27$	$F(1,52) = 45.4$<br>**$p < 0.001$**	$F(1,52) = 0.48$<br>$p = 0.48$	$F(1,52) = 4.30$<br>**$p = 0.04$**	$F(1,52) = 0.99$<br>$p = 0.32$<br>(19%)
	MAGL	$F(1,52) = 21.1$<br>**$p < 0.001$**	$F(1,52) = 13.3$<br>**$p < 0.001$**	$F(1,52) = 3.54$<br>$p = 0.06$	$F(1,52) = 20.5$<br>**$p < 0.001$**	$F(1,52) = 2.62$<br>$p = 0.11$	$F(1,52) = 0.94$<br>$p = 0.33$	$F(1,52) = 3.68$<br>$p = 0.06$<br>(54%)
Amg	CB_1R	$F(1,54) = 0.22$<br>$p = 0.64$	$F(1,54) = 2.34$<br>$p = 0.13$	$F(1,54) = 0.14$<br>$p = 0.70$	$F(1,54) = 91.57$<br>**$p < 0.001$**	$F(1,54) = 0.33$<br>$p = 0.56$	$F(1,54) = 0.22$<br>$p = 0.63$	$F(1,54) = 3.26$<br>$p = 0.77$<br>(47%)
	CB_2R	$F(1,54) = 6.94$<br>**$p = 0.01$**<br>**F < M**	$F(1,54) = 5.40$<br>**$p = 0.02$**<br>**Tat(–) > Tat(+)**	$F(1,54) = 5.87$<br>**$p = 0.01$**<br>**Vehicle < CBD**	$F(1,54) = 9.27$<br>**$p = 0.004$**	$F(1,54) = 12.00$<br>**$p = 0.001$**	$F(1,54) = 5.73$<br>**$p = 0.02$**	$F(1,54) = 0.24$<br>$p = 0.622$<br>(8%)
	GPR55	$F(1,54) = 38.77$<br>**$p < 0.001$**<br>**F < M**	$F(1,54) = 163.5$<br>**$p < 0.001$**<br>**Tat(–) > Tat(+)**	$F(1,54) = 1.01$<br>$p = 0.31$	$F(1,54) = 114.0$<br>**$p < 0.001$**	$F(1,54) = 4.13$<br>**$p = 0.04$**	$F(1,54) = 0.82$<br>$p = 0.36$	$F(1,54) = 0.69$<br>$p = 0.40$<br>(14%)
	FAAH	$F(1,54) = 0.48$<br>$p = 0.49$	$F(1,54) = 16.14$<br>**$p < 0.001$**<br>**Tat(–) > Tat(+)**	$F(1,54) = 0.07$<br>$p = 0.78$	$F(1,54) = 6.81$<br>**$p = 0.01$**	$F(1,54) = 6.73$<br>**$p = 0.01$**	$F(1,54) = 1.75$<br>$p = 0.19$	$F(1,54) = 1.12$<br>$p = 0.29$<br>(19%)
	MAGL	$F(1,54) = 107.8$<br>**$p < 0.001$**<br>**F > M**	$F(1,54) = 10.64$<br>**$p = 0.002$**<br>**Tat(–) > Tat(+)**	$F(1,54) = 1.09$<br>$p = 0.30$	$F(1,54) = 22.32$<br>**$p < 0.001$**	$F(1,54) = 0.42$<br>$p = 0.51$	$F(1,54) = 0.07$<br>$p = 0.78$	$F(1,54) = 0.21$<br>$p = 0.64$<br>(7%)
BS	CB_1R	$F(1,54) = 54.03$<br>**$p < 0.001$**<br>**F < M**	$F(1,54) = 0.37$<br>$p = 0.54$	$F(1,54) = 0.99$<br>$p = 0.32$	$F(1,54) = 0.76$<br>$p = 0.38$	$F(1,54) = 0.52$<br>$p = 0.47$	$F(1,54) = 0.91$<br>$p = 0.34$	$F(1,54) = 0.04$<br>$p = 0.83$<br>(5%)
	CB_2R	$F(1,54) = 39.5$<br>**$p < 0.001$**<br>**F < M**	$F(1,54) = 6.15$<br>**$p = 0.01$**<br>**Tat(–) > Tat(+)**	$F(1,54) = 0.96$<br>$p = 0.33$	$F(1,54) = 3.10$<br>$p = 0.08$	$F(1,54) = 0.02$<br>$p = 0.87$	$F(1,54) = 1.00$<br>$p = 0.32$	$F(1,54) = 1.80$<br>$p = 0.18$<br>(29%)
	GPR55	$F(1,54) = 120.4$<br>**$p < 0.001$**<br>**F > M**	$F(1,54) = 0.29$<br>$p = 0.59$	$F(1,54) = 0.02$<br>$p = 0.88$	$F(1,54) = 14.5$<br>**$p < 0.001$**	$F(1,54) = 2.64$<br>$p = 0.11$	$F(1,54) = 0.08$<br>$p = 0.77$	$F(1,54) = 3.96$<br>**$p = 0.05$**<br>(55%)

	FAAH	$F(1,54) = 15.25$<br>**$p < 0.001$**<br>**F < M**	$F(1,54) = 42.2$<br>**$p < 0.001$**<br>**Tat(–) > Tat(+)**	$F(1,54) = 1.71$<br>$p = 0.19$	$F(1,54) = 0.48$<br>$p = 0.49$	$F(1,54) = 0.57$<br>$p = 0.45$	$F(1,54) = 9.17$<br>**$p = 0.004$**	$F(1,54) = 2.90$<br>$p = 0.09$<br>(43%)
	MAGL	$F(1,54) = 0.70$<br>$p = 0.40$	$F(1,54) = 29.40$<br>**$p < 0.001$**<br>**Tat(–) > Tat(+)**	$F(1,54) = 2.59$<br>$p = 0.11$	$F(1,54) = 2.86$<br>$p = 0.09$	$F(1,54) = 4.93$<br>**$p = 0.03$**	$F(1,54) = 4.00$<br>**$p = 0.05$**	$F(1,54) = 5.77$<br>**$p = 0.02$**<br>(71%)
SC	CB$_1$R	$F(1,54) = 12.98$<br>**$p < 0.001$**<br>**F < M**	$F(1,54) = 0.23$<br>$p = 0.62$	$F(1,54) = 2.03$<br>$p = 0.15$	$F(1,54) = 0.002$<br>$p = 0.96$	$F(1,54) = 1.01$<br>$p = 0.31$	$F(1,54) = 0.00$<br>$p = 0.99$	$F(1,54) = 0.21$<br>$p = 0.64$<br>(7%)
	CB$_2$R	$F(1,54) = 41.05$<br>**$p < 0.001$**<br>**F < M**	$F(1,54) = 9.22$<br>**$p = 0.004$**<br>**Tat(–) > Tat(+)**	$F(1,54) = 3.95$<br>**$p = 0.05$**<br>**Vehicle < CBD**	$F(1,54) = 30.73$<br>**$p < 0.001$**	$F(1,54) = 2.28$<br>$p = 0.13$	$F(1,54) = 16.39$<br>**$p < 0.001$**	$F(1,54) = 8.00$<br>**$p = 0.007$**<br>(85%)
	GPR55	$F(1,54) = 14.64$<br>**$p < 0.001$**<br>**F < M**	$F(1,54) = 0.116$<br>$p = 0.73$	$F(1,54) = 0.10$<br>$p = 0.74$	$F(1,54) = 2.65$<br>$p = 0.10$	$F(1,54) = 0.004$<br>$p = 0.94$	$F(1,54) = 9.84$<br>**$p = 0.003$**	$F(1,54) = 2.55$<br>$p = 0.11$<br>(39%)
	FAAH	$F(1,54) = 1.18$<br>$p = 0.28$	$F(1,54) = 20.82$<br>**$p < 0.001$**<br>**Tat(–) > Tat(+)**	$F(1,54) = 0.23$<br>$p = 0.63$	$F(1,54) =12.63$<br>**$p < 0.001$**	$F(1,54) = 2.02$<br>$p = 0.16$	$F(1,54) = 2.27$<br>$p = 0.13$	$F(1,54) = 13.30$<br>**$p < 0.001$**<br>(96%)
	MAGL	$F(1,54) = 0.67$<br>$p = 0.41$	$F(1,54) = 14.40$<br>**$p < 0.001$**<br>**Tat(–) > Tat(+)**	$F(1,54) = 0.02$<br>$p = 0.87$	$F(1,54) = 0.03$<br>$p = 0.85$	$F(1,54) = 3.48$<br>$p = 0.06$	$F(1,54) = 0.005$<br>$p = 0.94$	$F(1,54) = 3.57$<br>$p = 0.06$<br>(51%)

PFC, prefrontal cortex; Amg, amygdala; BS, brainstem, CB$_1$R, cannabinoid receptor type 1; CB$_2$R, cannabinoid receptor type 2; FAAH, fatty acid amide hydrolase; MAGL, monoacylglycerol lipase; SC, spinal cord.
