# Supplementary material for: Chronic CBD treatment differentially modulates neurobehavioral outcomes and endocannabinoid signaling in an aged HIV-1 Tat transgenic mouse model
Source: PLoS One. 2026 Jul 20;21(7):e0353267. doi: 10.1371/journal.pone.0353267 (PMC13384326; doi:10.1371/journal.pone.0353267)
Supplement: S4_File — (PDF) [file pone.0353267.s004.pdf]

S4_Table: Two-way ANOVA showing CBD and its metabolites in cortex and plasma.

Sample	CBD / Metabolite	Sex effect *F, p*	Genotype effect *F, p*	Sex x Genotype *F, p*
Cortex	CBD (ng/mL)	$F(1,26) = 0.65$<br>$p = 0.42$	$F(1,26) = 4.60$<br>**$p = 0.04$**<br><br>**Tat(–) > Tat(+)**	$F(1,26) = 4.47$<br>**$p = 0.04$**
	CBD-COOH (ng/mL)	ND	ND	ND
Plasma	CBD (ng/mL)	$F(1,26) = 1.56$<br>$p = 0.22$	$F(1,26) = 0.90$<br>$p = 0.34$	$F(1,26) = 5.28$<br>**$p = 0.03$**
	CBD-COOH (ng/mL)	$F(1,26) = 8.46$<br>**$p = 0.007$**<br><br>**F > M**	$F(1,26) = 0.02$<br>$p = 0.88$	$F(1,26) = 0.36$<br>$p = 0.55$

ND, not detected
