# Supplementary figures and images for: Chronic CBD treatment differentially modulates neurobehavioral outcomes and endocannabinoid signaling in an aged HIV-1 Tat transgenic mouse model
Source: PLoS One. 2026 Jul 20;21(7):e0353267. doi: 10.1371/journal.pone.0353267 (PMC13384326; doi:10.1371/journal.pone.0353267)

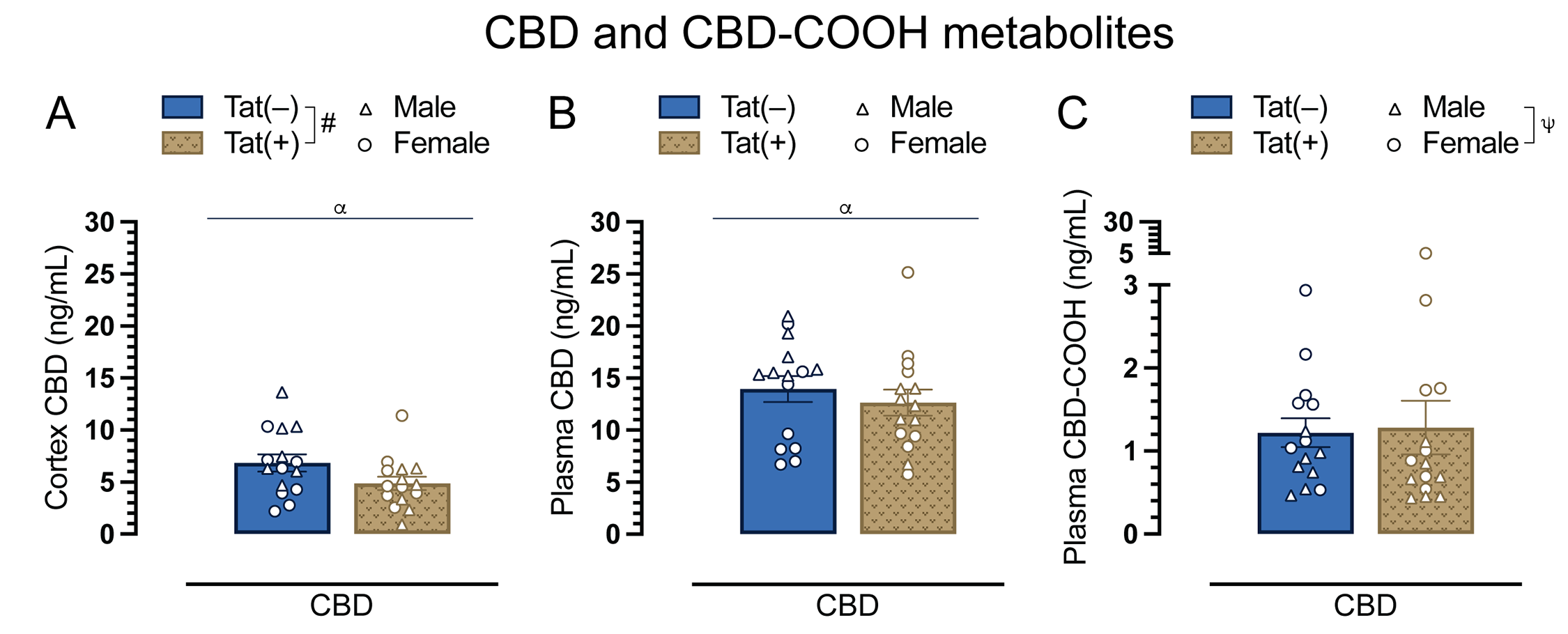

Supplement: S1_Fig — (A) Levels of CBD (ng/mL) in (A) cortex and (B) plasma. Levels of CBD-COOH (ng/mL) in plasma (C). Note: CBD-COOH levels in the cortex were not detected. Data represented as mean ± SEM. Statistical significance was assessed by overall ANOVAs, #p < 0.05 main effect of genotype, ψp < 0.05 main effect of sex, αp < 0.05 sex x genotype interaction. CBD dose = 3 mg/kg. N = 30(16f). (TIF) [file pone.0353267.s005.tif]

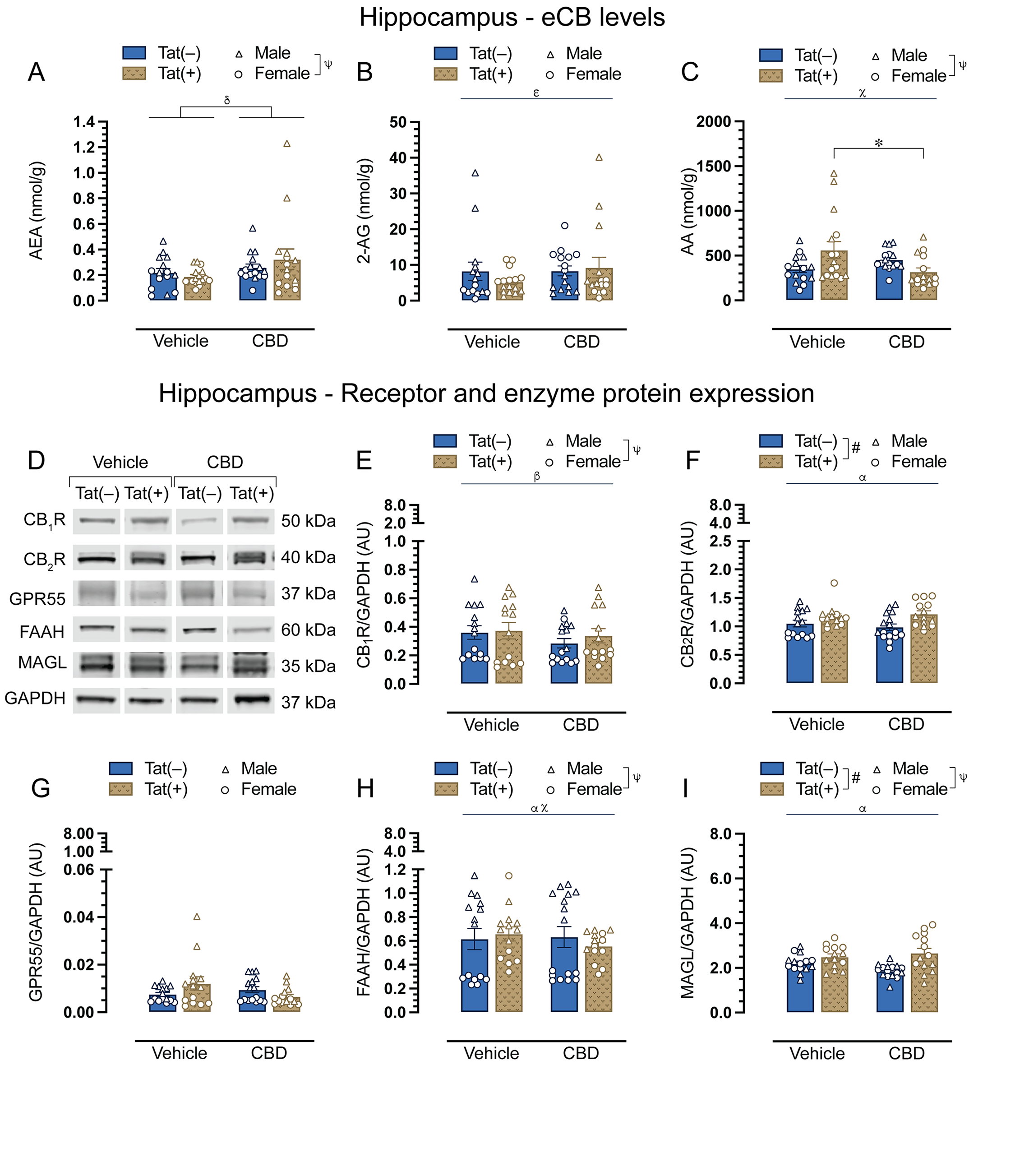

Supplement: S2_Fig — (A) AEA, (B) 2-AG, and (C) AA levels in the hippocampus. (D) Representative Western blot bands for CB1R, CB2R, GPR55, FAAH, MAGL, and loading control GAPDH. (E) CB1R (F) CB2R, (G) GPR55, (H) FAAH, and (I) MAGL protein expression in the hippocampus. Data represented as mean ± SEM. Statistical significance was assessed by overall ANOVAs, #p < 0.05 main effect of genotype, ψp < 0.05 main effect of sex, δp < 0.05 main effect of treatment, αp < 0.05 sex x genotype interaction, βp < 0.05 sex x treatment interaction, χp < 0.05 genotype x treatment interaction, εp < 0.05 sex x genotype x treatment interaction. CBD dose = 3 mg/kg. N = 62(31f) for eCB data and N = 59(29f) for western blot data. (TIF) [file pone.0353267.s006.tif]

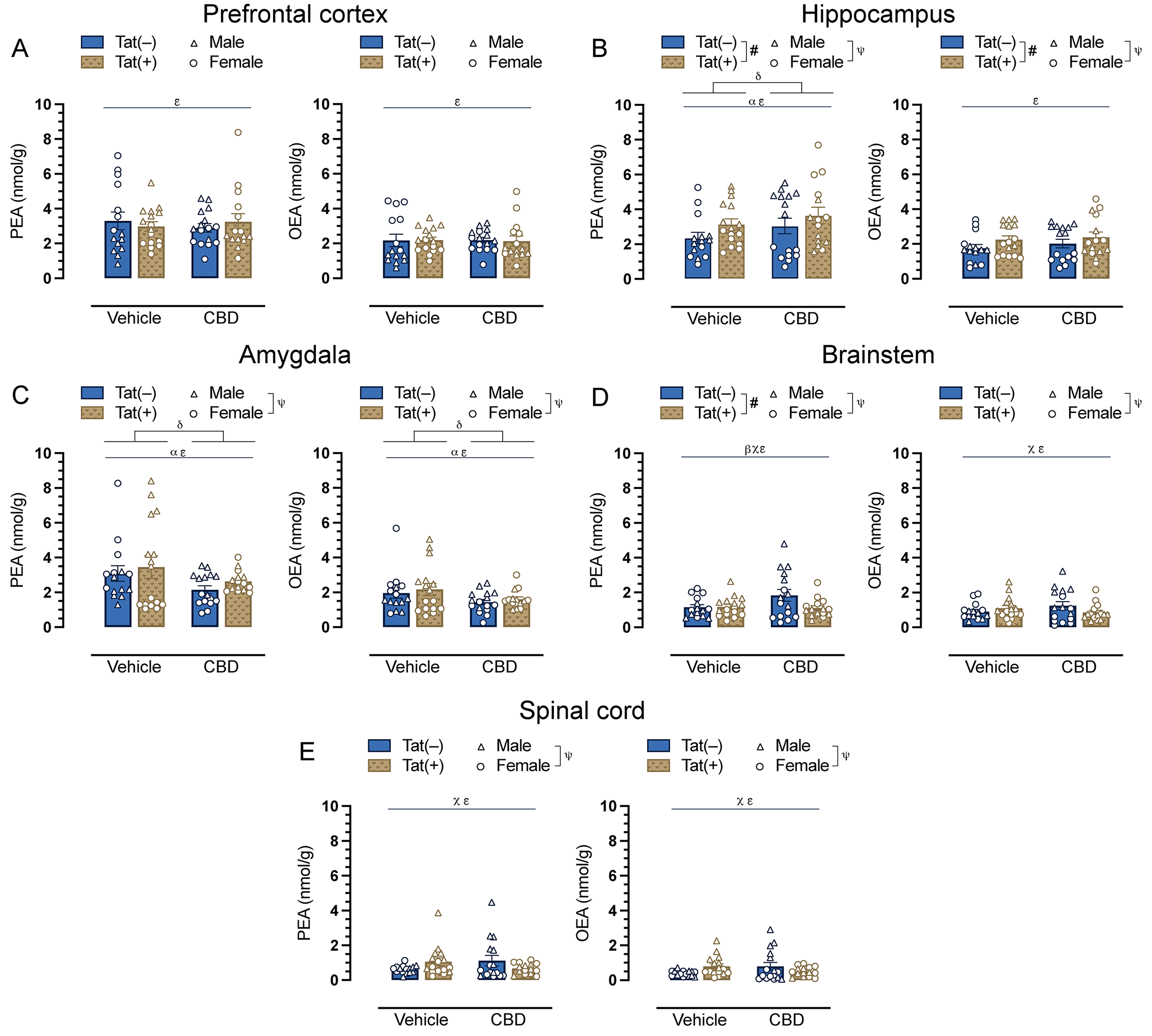

Supplement: S3_Fig — PEA and OEA levels in (A) PFC, (B) hippocampus, (C) amygdala, (D) brainstem, and (E) spinal cord. Data represented as mean ± SEM. Statistical significance was assessed by overall ANOVAs, #p < 0.05 main effect of genotype, ψp < 0.05 main effect of sex, δp < 0.05 main effect of treatment. A four-way mixed ANOVA for body mass; τp < 0.05 main effect of time, σp < 0.05 main time x sex x treatment interaction and γp < 0.05 main time x genotype x treatment interaction. CBD dose = 3 mg/kg. N = 62(31f). (TIF) [file pone.0353267.s007.tif]

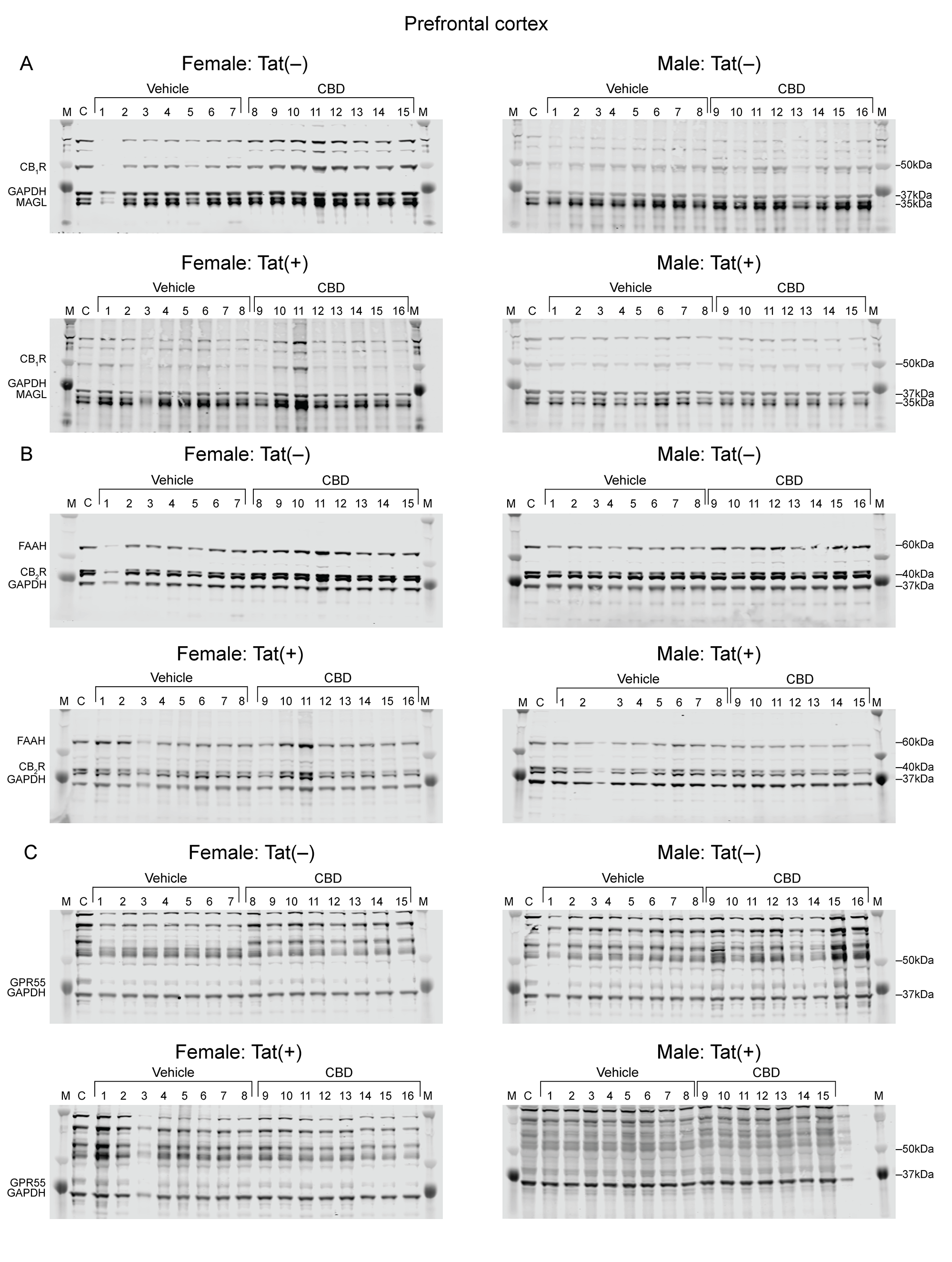

Supplement: S4_Fig — Images show (A) CB1R, MAGL, and GAPDH, (B) CB2R, FAAH, and GAPDH, and (C) GPR55 for female Tat(–), male Tat(–), female Tat(+), and male Tat(+) mice. C: positive control; M: molecular weights of marker protein (kDa). (TIF) [file pone.0353267.s008.tif]

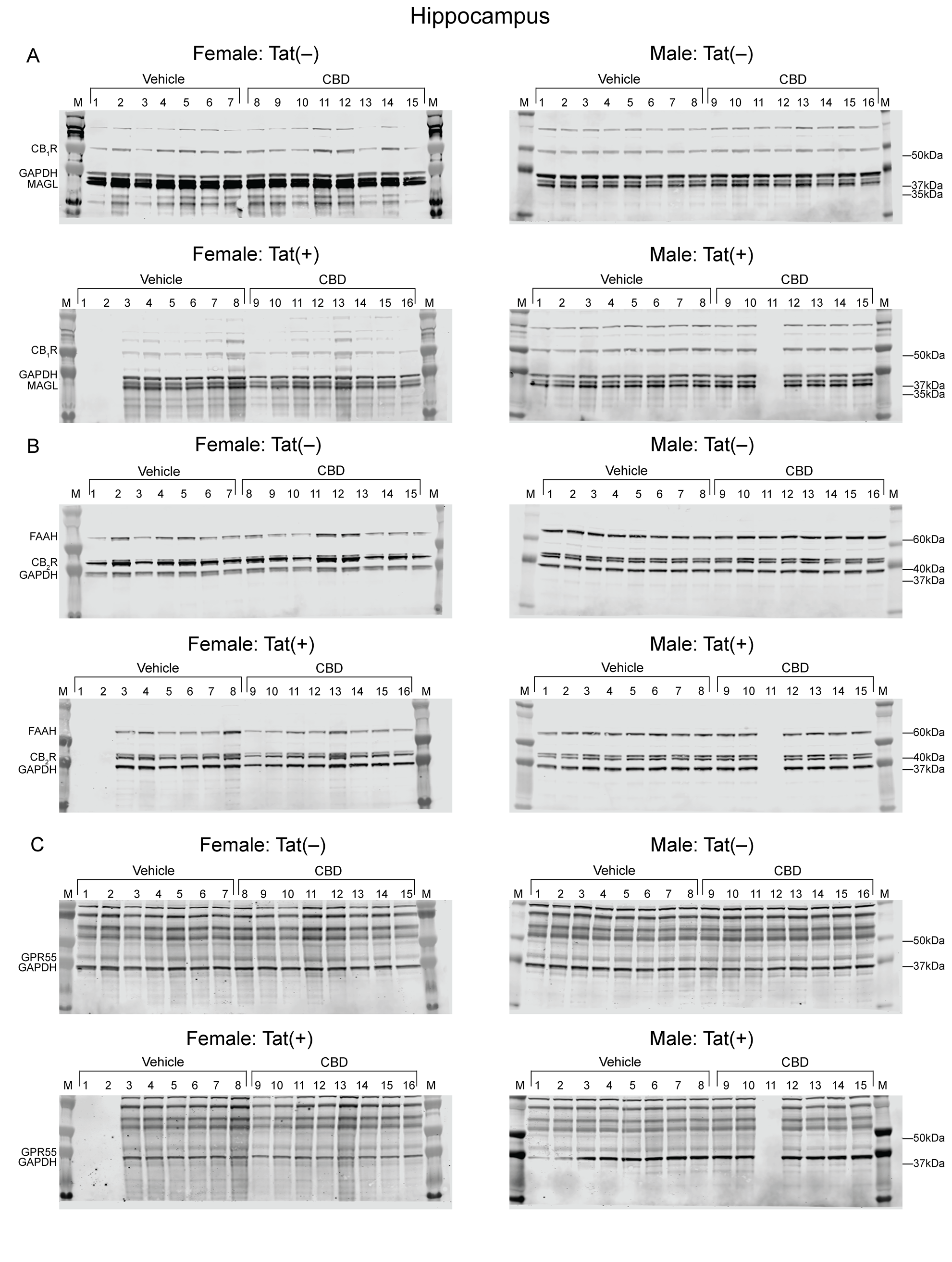

Supplement: S5_Fig — Images show (A) CB1R, MAGL, and GAPDH, (B) CB2R, FAAH, and GAPDH, and (C) GPR55 for female Tat(–), male Tat(–), female Tat(+), and male Tat(+) mice. M: molecular weights of marker protein (kDa). Note, 2 samples in female Tat(+) group and 1 sample in male Tat(+) group were lost during harvest. (TIF) [file pone.0353267.s009.tif]

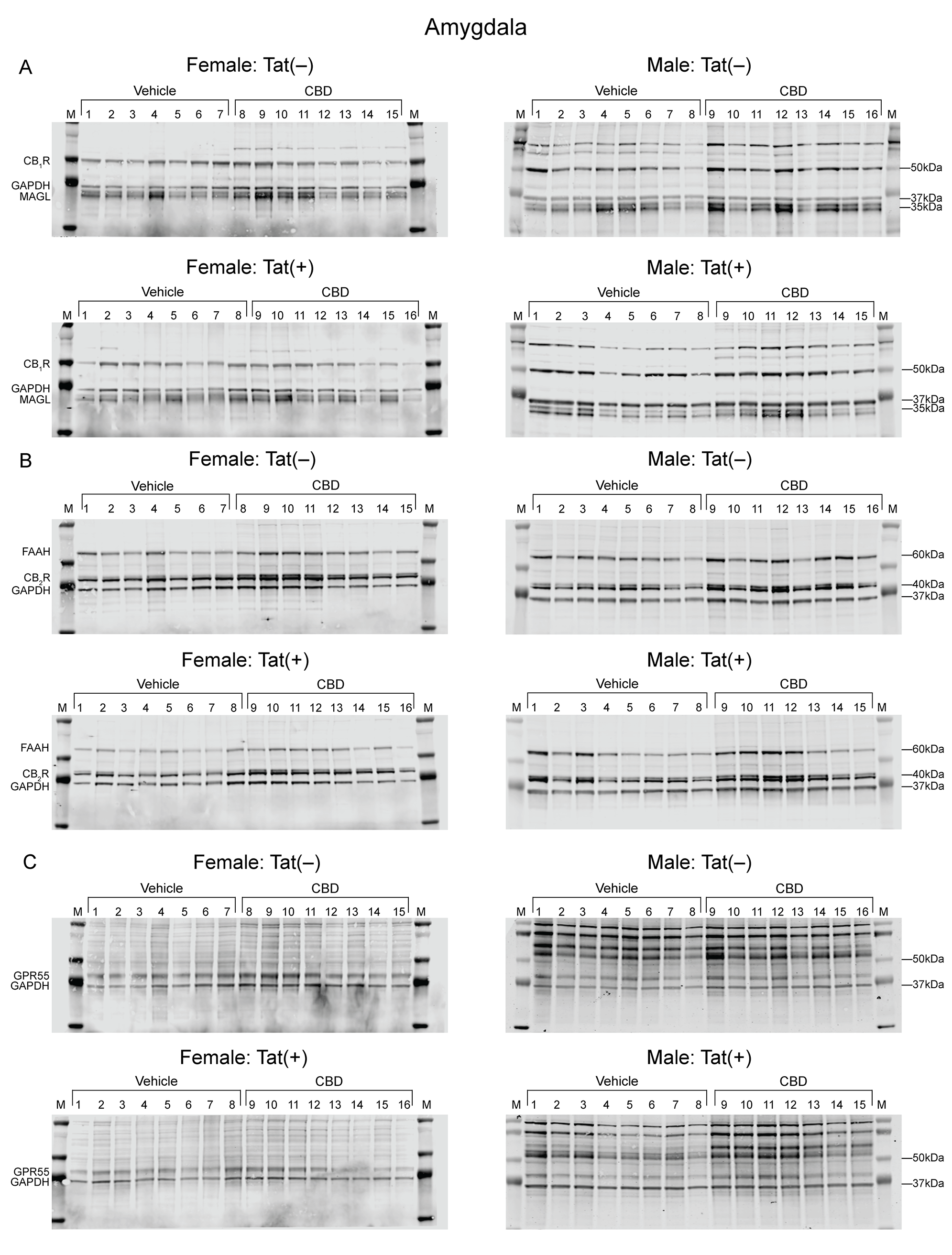

Supplement: S6_Fig — Images show (A) CB1R, MAGL, and GAPDH, (B) CB2R, FAAH, and GAPDH, and (C) GPR55 for female Tat(–), male Tat(–), female Tat(+), and male Tat(+) mice. M: molecular weights of marker protein (kDa). (TIF) [file pone.0353267.s010.tif]

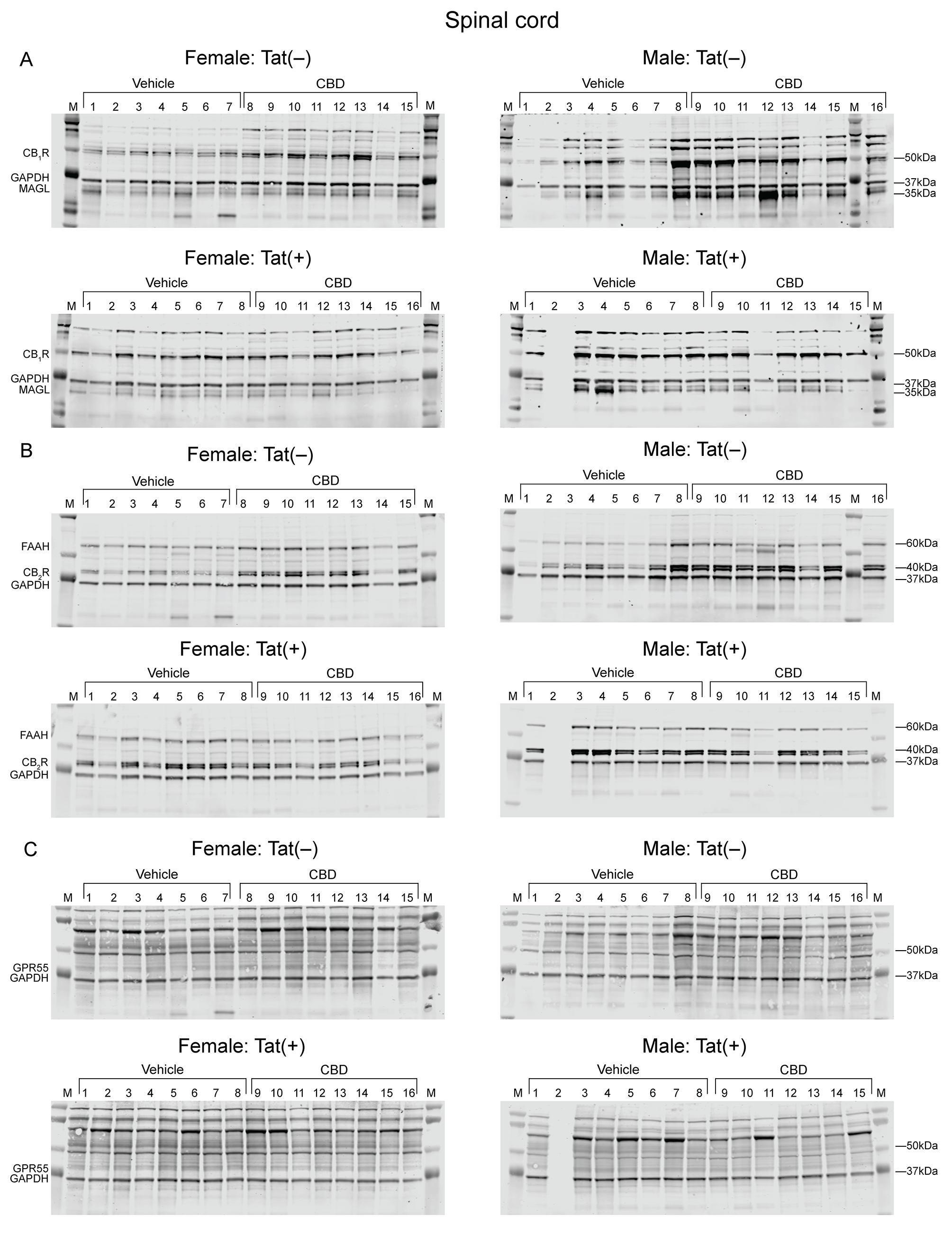

Supplement: S7_Fig — Images show (A) CB1R, MAGL, and GAPDH, (B) CB2R, FAAH, and GAPDH, and (C) GPR55 for female Tat(–), male Tat(–), female Tat(+), and male Tat(+) mice. M: molecular weights of marker protein (kDa). (TIF) [file pone.0353267.s011.tif]

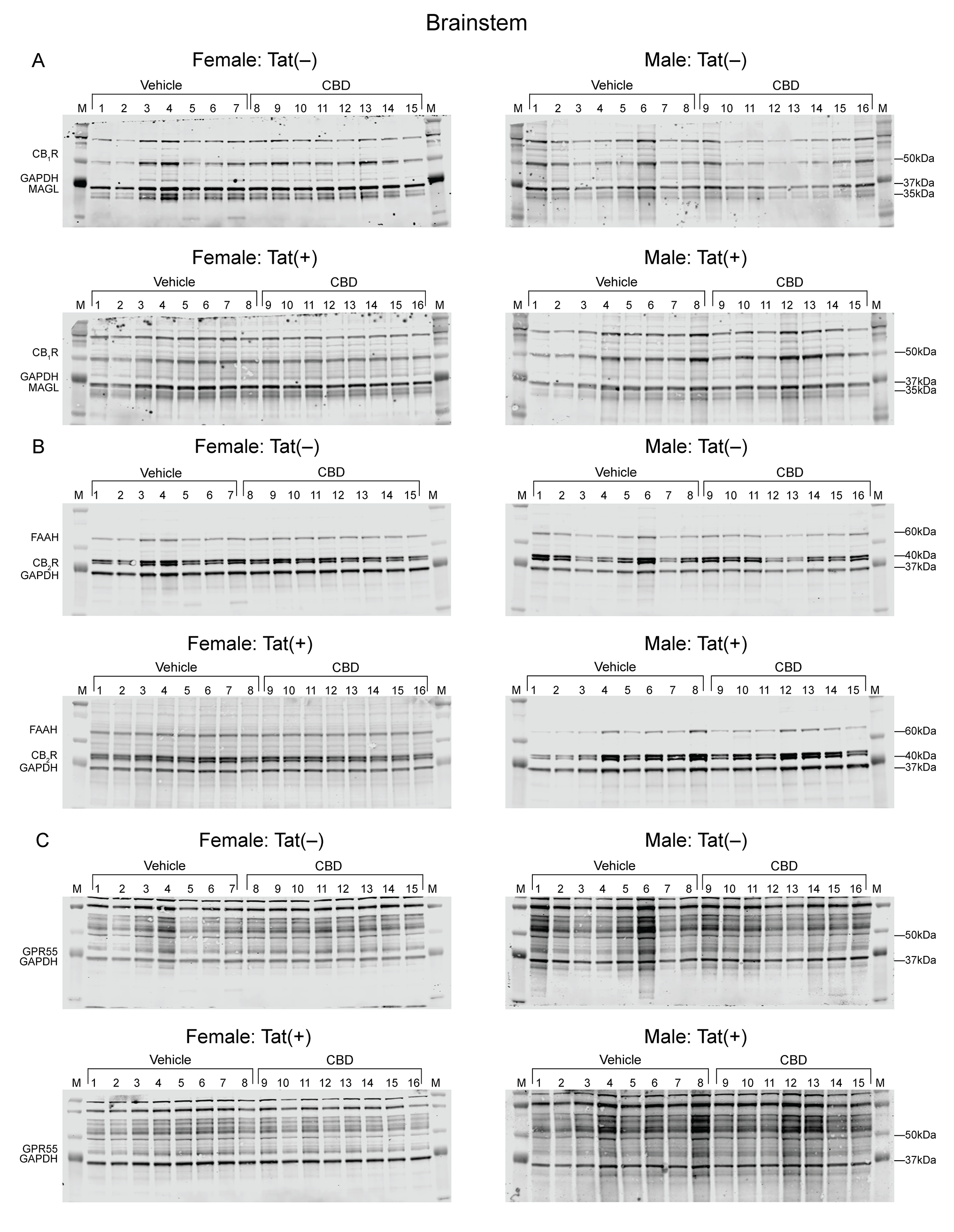

Supplement: S8_Fig — Images show (A) CB1R, MAGL, and GAPDH, (B) CB2R, FAAH, and GAPDH, and (C) GPR55 for female Tat(–), male Tat(–), female Tat(+), and male Tat(+) mice. M: molecular weights of marker protein (kDa). (TIF) [file pone.0353267.s012.tif]
