# Supplementary material for: Chronic CBD treatment differentially modulates neurobehavioral outcomes and endocannabinoid signaling in an aged HIV-1 Tat transgenic mouse model
Source: PLoS One. 2026 Jul 20;21(7):e0353267. doi: 10.1371/journal.pone.0353267 (PMC13384326; doi:10.1371/journal.pone.0353267)
Supplement: Attachment — Submitted filename: Chronic CBD_Response to Reviewers.docx [file pone.0353267.s014.docx]

**Response to Reviewers**

**Submission title: Chronic CBD treatment differentially modulates neurobehavioral outcomes and endocannabinoid signaling in an aged HIV-1 Tat transgenic mouse model**

We sincerely appreciate the effort in reviewing the manuscript and greatly appreciate all the reviewers’ suggestions and comments, which have significantly improved the quality of the manuscript. In revising the manuscript, we also noticed and corrected several additional minor errors. *All* *our responses to the reviewers are noted in blue, italicized text below.* Changes made in the text of the revised manuscript are highlighted in orange (non-italicized).

Reviewer #1: The study addresses a clinically relevant and understudied question—the effects of prolonged CBD exposure on neuroHIV in an aging context and represents a meaningful extension of the group’s prior work on acute CBD exposure in this model. The inclusion of sex as a biological variable across behavioral, lipidomic, and proteomic endpoints strengthens the translational relevance of the findings. However, several limitations should be addressed and/or discussed.

1. While all very important factors, the sample size is small to assess sex*genotype*treatment. It is impressive that the differences in readouts reached significance, but a post-hoc power analysis should be included with discussion of plans to affirm the findings in a larger cohort.

*We thank the reviewer for this constructive point. We agree that a complex three-way interaction (sex x genotype x treatment) requires careful power considerations. As requested, we have conducted a post-hoc power analysis using G*Power (see image below) for all three-way interactions, which are now detailed in Supplemental Tables S1, S2, and S3.*

*Our analysis revealed that the majority of the readouts achieved a power >80%. However, we acknowledge that certain measures, including AEA and AA in the amygdala and GPR55 expression in the brainstem, fell below the 80% threshold despite reaching statistical significance. We recognize that these specific findings, while significant, should be interpreted with caution. Accordingly, we have updated our Materials and Methods to reflect the power analysis protocol and have added a statement to the Discussion section acknowledging the exploratory nature of these lower-powered interactions and the necessity for future validation in a larger cohort.*

*We have included the following statement in the Statistical analysis part of the Materials and Material and methods*

“To assess the statistical reliability of the reported three-way interactions (sex x genotype x treatment), a post-hoc power analysis was conducted using G*Power 3.1. Achieved power for these interactions is reported in Supplemental Tables S1–S3.”

*Additionally, the following statement is now added to the discussion.*

“Sixth, although the study was sufficiently powered to detect major effects, some regional molecular shifts achieved significance with power below 80%. Future investigations with expanded sample sizes should be used to further confirm the strength of these specific three-way interactions.”

2. There is much discussion about the potential protective effects of CBD on the endocannabinoid system (ECS) and brain function, but there is little acknowledgement of the literature on state of the ECS in the brain (and systemic) in people with HIV. Considering the study is on the effects of CBD in a tat mouse, this seems pertinent to the intro and discussion.

*We appreciate this suggestion and agree that incorporating clinical data on the eCB system in PLWH strengthens the manuscript's rationale. We have added these pertinent details to the Introduction to better frame our study within the context of human pathology.*

“Clinical evidence indicates a severe disruption in eCB tone, marked by significantly lower levels of AEA (PMID: 39156649) and OEA (PMID: 37093248) potentially driven by enhanced FAAH-mediated degradation in addition to altered 2-AG dynamics (PMID: 39156649). Furthermore, post-mortem tissue from individuals with HIV encephalitis (HIVE) and HAND on cART reveals a marked upregulation of CB_1_R and CB_2_R receptors in microglia, macrophages, and astrocytes (PMID: 21450051, PMID: 34578323), as well as a pathological shift in neuronal CB_1_R localization from a punctate distribution to the soma that correlates with cognitive decline (PMID: 34578323). This altered pathological landscape underscores the necessity of targeting the eCB system.”

3. The data availability section appears to be missing important information... XXX

*Thank you for bringing this to our attention. All raw data generated in this manuscript is now deposited on the UNC Dataverse and will be published by the resubmission of this manuscript and can be found as Yadav-Samudrala, Barkha. 2026. “Chronic CBD Treatment Differentially Modulates Neurobehavioral Outcomes and Endocannabinoid Signaling in an Aged HIV-1 Tat Transgenic Mouse Model.” UNC Dataverse.* [*https://doi.org/10.15139/S3/WGHAYI*](https://doi.org/10.15139/S3/WGHAYI)*. Following information has been added to the “Data availability” section:*

“All raw data and western blot files generated in this study can be found at the UNC Dataverse: Yadav-Samudrala, Barkha. 2026. “Chronic CBD Treatment Differentially Modulates Neurobehavioral Outcomes and Endocannabinoid Signaling in an Aged HIV-1 Tat Transgenic Mouse Model.” UNC Dataverse. <https://doi.org/10.15139/S3/WGHAYI>.”

4. Memory was tested in the mice and some lipids were measured in the hippocampus, but no western blots of the hippocampus. This should be added or discussed.

*We thank the reviewer for this suggestion. We have now performed Western blot analysis on hippocampal tissue for CB1R, CB2R, GPR55, FAAH, and MAGL to complement our lipidomic and behavioral data. These results have been added to the Supplemental Information (S3_Table, S2_Fig, S3_Fig). We have also added raw unedited western blot images (S5_Fig).*

5. While not entirely necessary, it is surprising that no measures of neuroinflammation were included, ie GFAP, IBA1, TNFa.

*We appreciate the reviewer’s perspective on the importance of neuroinflammatory markers such as GFAP, IBA1, and TNF-α. While we agree these are critical components of HAND pathology, the primary objective of this study was to provide a high-resolution characterization of the eCB system and behavioral alterations following chronic CBD exposure. Due to the technical requirements of quantifying eCB ligands via mass spectrometry and the comprehensive profiling of five distinct proteins (CB_1_R, CB_2_R, GPR55, FAAH, and MAGL) via Western blot, the available tissue was fully utilized for these specific measurements. While our laboratory has an established record of assessing neuroinflammatory cytokines (e.g., IFN-γ, IL-1β) in other models of HIV such as HIV-1 Tg26, including our recent work in HIV-1 Tg26 mice (PMID: 41654202), we elected to maintain a strict focus on the eCB system in this manuscript to ensure a cohesive and deep mechanistic analysis. We have now acknowledged the exclusion of these markers as a limitation in the revised Discussion.*

“Fifth, it is important to note that the current study focused exclusively on behavioral outcomes and the molecular profiling of the eCB system. Therefore, future studies integrating high-throughput eCB ligand analysis with localized cytokine profiling will be essential to fully elucidate the reciprocal relationship between CBD-induced eCB modulation and the attenuation of neuroinflammation in the Tat model.”

Reviewer #2: PONE-D-26-10019: Peer-Review Comments

This study examines the effects of chronic cannabidiol (CBD) treatment on aged HIV-1 Tat transgenic mice, investigating behavioral outcomes and changes in endocannabinoid system. The authors employed a 12-week CBD treatment protocol (3 mg/kg, s.c.) in 15-18 month-old mice, assessing cognition, anxiety-like behavior, nociception, and endocannabinoid signaling across multiple CNS regions. Key findings include gender-specific improvements in recognition memory (particularly in females), significantly altered thermal nociception, and complex region-dependent restructuring of the endocannabinoid system.
The work is supported by rigorous experimental design, clinically relevant aged model, multi-modal assessments across multiple brain regions, and inclusion of sex as a biological variable. The study's longitudinal approach with multi-modal assessments (behavioral, biochemical) across four CNS regions (prefrontal cortex, amygdala, brainstem, spinal cord) provides valuable temporal and mechanistic insights. The inclusion of both sexes as a biological variable is particularly commendable given known sex differences in cannabinoid responses. Using aged mice addresses a critical gap in the literature, as most prior studies focus on young adults. This is highly relevant given the aging population of people living with HIV. The endocannabinoid system mapping is thorough. The combination of mass spectrometry for endocannabinoid ligand quantification and Western blotting for protein expression provides robust molecular characterization. The authors acknowledge and report three-way interactions (sex × genotype × treatment), demonstrating statistical sophistication and avoiding oversimplification of complex biological phenomena. Authors also acknowledge important limitations, for example, authors acknowledge that bulk tissue analysis cannot distinguish neuronal vs. glial contributions, which is critical given that Tat expression is astrocyte-driven in this model.

Specific Comments:

1. The authors report CBD-induced hyperthermia as a "critical physiological finding" (Page 31) yet continue to interpret/ discuss hot plate results as hyperalgesia. If baseline body temperature is elevated, isn’t the thermal gradient between body and hot plate is reduced? mechanically decreasing latency regardless of true nociceptive sensitivity? While the authors acknowledge this confounding issue ("explaining the paradoxical reduction in hot-plate latencies"), authors may need to adjust their conclusions accordingly, and revise the entire supra-spinal nociception narrative or, at minimum, explicitly state the limitations in discussion with regards to the extent of reliability of thermal nociception assessed in this context. Focusing solely on the thermoregulatory mechanisms and removing interpretive claims about hyperalgesia should be considered.

*We sincerely appreciate the reviewer’s point regarding the physical mechanics of a thermal gradient; our empirical data demonstrates that the core temperature variance between groups was far too small to introduce a physical artifact.*

*In our study, the mean baseline body temperature for the chronic CBD group was 36.20°C, compared to 36.11°C in the vehicle control group. This represents an absolute physiological difference of only 0.09°C. Given that our hot-plate apparatus was maintained at a constant  55°C, this tiny baseline shift alters the physical thermal gradient (ΔT) between the animal's paw and the testing surface by less than 0.5%.*

*Calculation:*

- *Hot plate was set to 55°C:*
  - *Vehicle Gradient: 55 − 36.11 = 18.89°C*
  - *CBD Gradient: 55 − 36.20 = 18.80°C*
  - *The physical difference in the gradient = 0.09°C (0.47%)*

*Mathematically, less than* ***0.5% physical variance*** *in a thermal gradient cannot possibly account for a statistically significant change in behavioral latency. Instead, our results revealed a robust, statistically significant reduction in hot-plate latency following CBD treatment (p<0.05). Because the magnitude of the behavioral response vastly exceeds this 0.09****°****C physical baseline shift, the data strongly support a true alteration in supraspinal nociceptive sensitivity rather than a thermoregulatory artifact.*

*We have now added the following statement to the discussion:*

“It is important to note that while chronic CBD induced a statistically significant elevation in body temperature, the difference between vehicle- and CBD-treated groups was only 0.09 °C; this negligible (<0.5%) shift in the physical thermal gradient between the paw and the 55 °C testing surface cannot mathematically account for the robust reduction in hot-plate latencies, confirming a true alteration in supraspinal nociceptive sensitivity.”

2. The "psychostimulant-like effect" (Page 32) attributed to Tat-mediated dopaminergic dysregulation appears speculative without dopamine measurements. This aspect can be clearly stated. Clearly mentioning these as hypotheses based on preliminary observations requiring future testing is recommended.

*We thank the reviewer for pointing out the speculative nature of attributing the observed hyperactivity to a 'psychostimulant-like effect' without direct dopamine measurements. We agree that while this is a well-supported mechanism in the existing Tat literature, it remains a hypothesis within the scope of our current dataset.*

*Following your recommendation, we have revised this section of the Discussion to explicitly state that this mechanistic explanation is a hypothesis based on our behavioral observations. We have tempered the language to ensure it reads as a potential pathway requiring future neurochemical validation rather than a definitive conclusion*

“We hypothesize that this hyperactivity may be driven by Tat-mediated dysregulation of the dopaminergic system, specifically by interfering with dopamine transporters to increase synaptic dopamine levels, yielding a psychostimulant-like effect.”

“However, because direct dopamine kinetics were not quantified in the current study, this mechanism remains speculative and requires future empirical testing.”

3. All assessments occurred at week 16-17, at least 72 hours after the final injection, if I am correct. No information is provided about CBD half-life or acute/chronic pharmaco-physiological levels, making it unclear whether observed effects reflect drug presence or long-term neuroplastic changes (or both). I recommend discussing this briefly.

*We thank the reviewer for highlighting this important pharmacological distinction. The reviewer is correct that behavioral and biochemical assessments were conducted 16–17 weeks into the protocol, approximately 72 hours following the final subcutaneous injection. Because CBD is highly lipophilic, chronic administration over 12 weeks likely results in tissue accumulation and a prolonged elimination half-life, creating a 'depot effect' in adipose and brain tissue. Therefore, we agree that the observed phenotypes and endocannabinoid alterations at the 72-hour mark likely reflect a combination of sustained steady-state drug presence and downstream, long-term neuroplastic adaptations.*

*Following your recommendation, we have added a brief discussion of CBD's pharmacokinetics in senescent models to the limitations section, explicitly clarifying this timeline for the reader.*

“First, it is important to contextualize the temporal relationship between our dosing regimen and behavioral testing. All assessment were conducted approximately 72 hours after the final chronic CBD treatment. Given the highly lipophilic nature of CBD, prolonged 12-week treatment should cause significant accumulation in lipid-rich compartments, including adipose tissue and the CNS, which can substantially extend the drug’s elimination kinetics via redistribution from tissue depots [170, 171]. While the acute plasma half-life of CBD in mice is relatively brief, prolonged chronic dosing regimens yield highly persistent, measurable concentrations of the drug directly in brain tissue [172]. Consequently, it remains unclear whether the reported behavioral and biochemical outcomes reflect the continuous, steady-state presence of residual CBD, permanent long-term neuroplastic adaptations within the eCB system, or a combination of both. Future studies incorporating explicit plasma and brain tissue clearance kinetics at multiple post-dosing intervals will be necessary to fully dissociate the acute physiological levels of CBD from enduring structural neuroplasticity.”

4. Tables 1-3 containing Tukey post-hoc comparisons should be moved to supplemental material. This improves readability while maintaining statistical rigor. Readers can grasp key patterns from annotated figures without navigating lengthy tables, while those requiring detailed comparisons can consult the supplement.

*We appreciate the reviewer’s suggestion to move Tables 1–3 to the supplemental material to streamline the narrative. We agree that minimizing dense data blocks can enhance readability; however, we respectfully request to maintain these tables within the main text of the manuscript due to the high dimensionality of our data.*

*While we have made every effort to visually represent key differences within our figures, the figures alone cannot fully capture the multifaceted nature of the two-way (sex x genotype, sex x treatment) and three-way (sex x genotype x treatment) interactions. Because biological sex is a core biological variable in this study, the post-hoc comparisons broken down by sex provide critical context that is central to our main conclusions, rather than secondary or supporting information.*

*To address the reviewer's valid concern regarding readability, we have heavily optimized the formatting of Tables 1–3. We have clearly bolded major significant interactions so that readers can quickly extract the critical statistical patterns without being overwhelmed by the lengthy text. We hope the reviewer agrees that keeping these comprehensive breakdowns accessible in the main manuscript ensures maximal transparency for these complex interactions.*

5. The discussion is lengthy and somewhat repetitive. Integration of findings across brain regions occurs sporadically rather than systematically. A structured synthesis organized by behavioral domain (cognition, affect, pain) rather than region-by-region analysis would make it more succinct while improving clarity. Extensive discussion of CBD's antioxidant properties, cerebral blood flow effects, and 5-HT1A/TRPV1 interactions is provided; while referencing supporting literature is appropriate, the narrative sometimes implies these mechanisms were demonstrated in the current study when they were not.

*We appreciate the reviewer's perspective regarding the length and repetitive elements within our original Discussion. While we have deliberately maintained our region-by-region structural layout because each paragraph corresponds directly to an isolated behavioral/biochemical brain area mapping (PFC, Amygdala, Spinal Cord, and Brainstem), we recognize the need to make the narrative more succinct and less speculative.*

*As requested, we have edited the discussion to condense the narrative and significantly temper our discussion of external mechanisms (such as antioxidant cascades, 5-HT1A binding profiles, and TRPV1 signaling mechanics). We have explicitly framed these pathways as literature-based hypotheses rather than demonstrated outcomes of our current data.*

“Therefore, we hypothesize that this thermoregulatory shift may involve an imbalance between 5-HT1A-mediated cooling and TRPV1-mediated thermogenesis [148, 149]. As established, aging significantly declines 5-HT1A receptor density, which may compromise the brain's primary cooling mechanism [150, 151], leaving unmonitored TRPV1 pathways highly sensitive to cannabinoid-driven or Tat-sensitized thermogenic signaling [142] Furthermore, steady-state pharmacokinetic accumulation from chronic s.c. administration in senescent animals with reduced metabolic clearance could continuously drive these metabolic or adipose shifts [152, 153], though direct receptor kinetics remain to be verified.”

This manuscript addresses a clinically relevant question using an appropriately aged animal model and comprehensive methodology. The sex-specific cognitive benefits of chronic CBD in the context of HIV-associated neurological complications represent a meaningful contribution.

*Thank you. We appreciate this comment.*
