# Supplementary material for: Chronic CBD treatment differentially modulates neurobehavioral outcomes and endocannabinoid signaling in an aged HIV-1 Tat transgenic mouse model
Source: PLoS One. 2026 Jul 20;21(7):e0353267. doi: 10.1371/journal.pone.0353267 (PMC13384326; doi:10.1371/journal.pone.0353267)
Supplement: Peer review history

# Author comment: Author response to Decision Letter 0

**DOI:** 10.1371/journal.pone.0353267.r001

**Transfer Alert**

This paper was transferred from another journal. As a result, its full editorial history (including decision letters, peer reviews and author responses) may not be present.

5 Mar 2026

# Peer review: Decision Letter 0

**DOI:** 10.1371/journal.pone.0353267.r002

**Authors:** Vinod K. Yaragudri

15 Apr 2026

PONE-D-26-10019

Chronic CBD treatment differentially modulates neurobehavioral outcomes and endocannabinoid signaling in an aged HIV-1 Tat transgenic mouse model

PLOS One

Dear Dr. Yadav-Samudrala,

Thank you for submitting your manuscript to PLOS ONE. After careful consideration, we feel that it has merit but does not fully meet PLOS ONE’s publication criteria as it currently stands. Therefore, we invite you to submit a revised version of the manuscript that addresses the points raised during the review process. As you see the reviewers have made some important and constructive comments that need to be addressed.

Please submit your revised manuscript by May 30 2026 11:59PM. If you will need more time than this to complete your revisions, please reply to this message or contact the journal office at plosone@plos.org. When you're ready to submit your revision, log on to https://www.editorialmanager.com/pone/ and select the ’submissions Needing Revision' folder to locate your manuscript file.

Please include the following items when submitting your revised manuscript:

- A letter that responds to each point raised by the academic editor and reviewer(s). You should upload this letter as a separate file labeled 'Response to Reviewers'.
- A marked-up copy of your manuscript that highlights changes made to the original version. You should upload this as a separate file labeled 'Revised Manuscript with Track Changes'.
- An unmarked version of your revised paper without tracked changes. You should upload this as a separate file labeled 'Manuscript'.

If you would like to make changes to your financial disclosure, please include your updated statement in your cover letter. Guidelines for resubmitting your figure files are available below the reviewer comments at the end of this letter.

If applicable, we recommend that you deposit your laboratory protocols in protocols.io to enhance the reproducibility of your results. Protocols.io assigns your protocol its own identifier (DOI) so that it can be cited independently in the future. For instructions see: https://journals.plos.org/plosone/s/submission-guidelines#loc-laboratory-protocols. Additionally, PLOS ONE offers an option for publishing peer-reviewed Lab Protocol articles, which describe protocols hosted on protocols.io. Read more information on sharing protocols at https://plos.org/protocols?utm_medium=editorial-email&utm_source=authorletters&utm_campaign=protocols.

As the corresponding author, your ORCID iD is verified in the submission system and will appear in the published article. PLOS supports the use of ORCID, and we encourage all coauthors to register for an ORCID iD and use it as well. Please encourage your coauthors to verify their ORCID iD within the submission system before final acceptance, as unverified ORCID iDs will not appear in the published article. Only the individual author can complete the verification step; PLOS staff cannot verify ORCID iDs on behalf of authors.

We look forward to receiving your revised manuscript.

Kind regards,

Vinod K. Yaragudri, M.Sc., Ph.D

Academic Editor

PLOS One

Journal Requirements:

When submitting your revision, we need you to address these additional requirements.

1. Please ensure that your manuscript meets PLOS ONE’s style requirements, including those for file naming. The PLOS ONE style templates can be found at

https://journals.plos.org/plosone/s/file?id=wjVg/PLOSOne_formatting_sample_main_body.pdf and

https://journals.plos.org/plosone/s/file?id=ba62/PLOSOne_formatting_sample_title_authors_affiliations.pdf

2. Thank you for stating the following financial disclosure:

"This work was supported by NIDA R01 DA055523 (S.F. and W.J.), R01 DA064394 (S.F.), P30 DA033934 (J.LP.)."

Please state what role the funders took in the study.  If the funders had no role, please state: "The funders had no role in study design, data collection and analysis, decision to publish, or preparation of the manuscript."

If this statement is not correct you must amend it as needed.

Please include this amended Role of Funder statement in your cover letter; we will change the online submission form on your behalf.

3. PLOS ONE now requires that authors provide the original uncropped and unadjusted images underlying all blot or gel results reported in a submission’s figures or Supporting Information files. This policy and the journal’s other requirements for blot/gel reporting and figure preparation are described in detail at https://journals.plos.org/plosone/s/figures#loc-blot-and-gel-reporting-requirements and https://journals.plos.org/plosone/s/figures#loc-preparing-figures-from-image-files. When you submit your revised manuscript, please ensure that your figures adhere fully to these guidelines and provide the original underlying images for all blot or gel data reported in your submission. See the following link for instructions on providing the original image data: https://journals.plos.org/plosone/s/figures#loc-original-images-for-blots-and-gels.

In your cover letter, please note whether your blot/gel image data are in Supporting Information or posted at a public data repository, provide the repository URL if relevant, and provide specific details as to which raw blot/gel images, if any, are not available. Email us at plosone@plos.org if you have any questions.

4. When completing the data availability statement of the submission form, you indicated that you will make your data available on acceptance. We strongly recommend all authors decide on a data sharing plan before acceptance, as the process can be lengthy and hold up publication timelines. Please note that, though access restrictions are acceptable now, your entire data will need to be made freely accessible if your manuscript is accepted for publication. This policy applies to all data except where public deposition would breach compliance with the protocol approved by your research ethics board. If you are unable to adhere to our open data policy, please kindly revise your statement to explain your reasoning and we will seek the editor’s input on an exemption. Please be assured that, once you have provided your new statement, the assessment of your exemption will not hold up the peer review process.

5. If the reviewer comments include a recommendation to cite specific previously published works, please review and evaluate these publications to determine whether they are relevant and should be cited. There is no requirement to cite these works unless the editor has indicated otherwise.

[Note: HTML markup is below. Please do not edit.]

Reviewers' comments:

Reviewer’s Responses to Questions

**Comments to the Author**

1. Is the manuscript technically sound, and do the data support the conclusions?

The manuscript must describe a technically sound piece of scientific research with data that supports the conclusions. Experiments must have been conducted rigorously, with appropriate controls, replication, and sample sizes. The conclusions must be drawn appropriately based on the data presented.

Reviewer #1: Partly

Reviewer #2: Yes

**********

2. Has the statistical analysis been performed appropriately and rigorously?

Reviewer #1: Yes

Reviewer #2: Yes

**********

3. Have the authors made all data underlying the findings in their manuscript fully available?

The PLOS Data policy requires authors to make all data underlying the findings described in their manuscript fully available without restriction, with rare exception (please refer to the Data Availability Statement in the manuscript PDF file). The data should be provided as part of the manuscript or its supporting information, or deposited to a public repository. For example, in addition to summary statistics, the data points behind means, medians and variance measures should be available. If there are restrictions on publicly sharing data—e.g. participant privacy or use of data from a third party—those must be specified.

Reviewer #1: No

Reviewer #2: Yes

**********

4. Is the manuscript presented in an intelligible fashion and written in standard English?

PLOS ONE does not copyedit accepted manuscripts, so the language in submitted articles must be clear, correct, and unambiguous. Any typographical or grammatical errors should be corrected at revision, so please note any specific errors here.

Reviewer #1: Yes

Reviewer #2: Yes

**********

5. Review Comments to the Author

Please use the space provided to explain your answers to the questions above. You may also include additional comments for the author, including concerns about dual publication, research ethics, or publication ethics. (Please upload your review as an attachment if it exceeds 20,000 characters)

Reviewer #1: The study addresses a clinically relevant and understudied question—the effects of prolonged CBD exposure on neuroHIV in an aging context, and represents a meaningful extension of the group’s prior work on acute CBD exposure in this model. The inclusion of sex as a biological variable across behavioral, lipidomic, and proteomic endpoints strengthens the translational relevance of the findings. However, several limitations should be addressed and/or discussed.

1. While all very important factors, the sample size is small to assess sex*genotype*treatment. It is impressive that the differences in readouts reached significance, but a post-hoc power analysis should be included with discussion of plans to affirm the findings in a larger cohort.

2. There is much discussion about the potential protective effects of CBD on the endocannabinoid system (ECS) and brain function, but there is little acknowledgement of the literature on state of the ECS in the brain (and systemic) in people with HIV. Considering the study is on the effects of CBD in a tat mouse, this seems pertinent to the intro and discussion.

3. The data availability section appears to be missing important information... XXX

4. Memory was tested in the mice and some lipids were measured in the hippocampus, but no western blots of the hippocampus. This should be added or discussed.

5. While not entirely necessary, it is surprising that no measures of neuroinflammation were included, ie GFAP, IBA1, TNFa.

Reviewer #2: PONE-D-26-10019: Peer-Review Comments

This study examines the effects of chronic cannabidiol (CBD) treatment on aged HIV-1 Tat transgenic mice, investigating behavioral outcomes and changes in endocannabinoid system. The authors employed a 12-week CBD treatment protocol (3 mg/kg, s.c.) in 15-18 month-old mice, assessing cognition, anxiety-like behavior, nociception, and endocannabinoid signaling across multiple CNS regions. Key findings include gender-specific improvements in recognition memory (particularly in females), significantly altered thermal nociception, and complex region-dependent restructuring of the endocannabinoid system.

The work is supported by rigorous experimental design, clinically relevant aged model, multi-modal assessments across multiple brain regions, and inclusion of sex as a biological variable. The study’s longitudinal approach with multi-modal assessments (behavioral, biochemical) across four CNS regions (prefrontal cortex, amygdala, brainstem, spinal cord) provides valuable temporal and mechanistic insights. The inclusion of both sexes as a biological variable is particularly commendable given known sex differences in cannabinoid responses. Using aged mice addresses a critical gap in the literature, as most prior studies focus on young adults. This is highly relevant given the aging population of people living with HIV. The endocannabinoid system mapping is thorough. The combination of mass spectrometry for endocannabinoid ligand quantification and Western blotting for protein expression provides robust molecular characterization. The authors acknowledge and report three-way interactions (sex × genotype × treatment), demonstrating statistical sophistication and avoiding oversimplification of complex biological phenomena. Authors also acknowledge important limitations, for example, authors acknowledge that bulk tissue analysis cannot distinguish neuronal vs. glial contributions, which is critical given that Tat expression is astrocyte-driven in this model.

Specific Comments:

1. The authors report CBD-induced hyperthermia as a "critical physiological finding" (Page 31) yet continue to interpret/ discuss hot plate results as hyperalgesia. If baseline body temperature is elevated, isn’t the thermal gradient between body and hot plate is reduced? mechanically decreasing latency regardless of true nociceptive sensitivity? While the authors acknowledge this confounding issue ("explaining the paradoxical reduction in hot-plate latencies"), authors may need to adjust their conclusions accordingly, and revise the entire supra-spinal nociception narrative or, at minimum, explicitly state the limitations in discussion with regards to the extent of reliability of thermal nociception assessed in this context. Focusing solely on the thermoregulatory mechanisms and removing interpretive claims about hyperalgesia should be considered.

2. The "psychostimulant-like effect" (Page 32) attributed to Tat-mediated dopaminergic dysregulation appears speculative without dopamine measurements. This aspect can be clearly stated. Clearly mentioning these as hypotheses based on preliminary observations requiring future testing is recommended.

3. All assessments occurred at week 16-17, at least 72 hours after the final injection, if I am correct. No information is provided about CBD half-life or acute/chronic pharmaco-physiological levels, making it unclear whether observed effects reflect drug presence or long-term neuroplastic changes (or both). I recommend discussing this briefly.

4. Tables 1-3 containing Tukey post-hoc comparisons should be moved to supplemental material. This improves readability while maintaining statistical rigor. Readers can grasp key patterns from annotated figures without navigating lengthy tables, while those requiring detailed comparisons can consult the supplement.

5. The discussion is lengthy and somewhat repetitive. Integration of findings across brain regions occurs sporadically rather than systematically. A structured synthesis organized by behavioral domain (cognition, affect, pain) rather than region-by-region analysis would make it more succinct while improving clarity. Extensive discussion of CBD’s antioxidant properties, cerebral blood flow effects, and 5-HT1A/TRPV1 interactions is provided; while referencing supporting literature is appropriate, the narrative sometimes implies these mechanisms were demonstrated in the current study when they were not.

This manuscript addresses a clinically relevant question using an appropriately aged animal model and comprehensive methodology. The sex-specific cognitive benefits of chronic CBD in the context of HIV-associated neurological complications represent a meaningful contribution.

**********

6. PLOS authors have the option to publish the peer review history of their article (what does this mean?). If published, this will include your full peer review and any attached files.

If you choose “no”, your identity will remain anonymous but your review may still be made public.

**Do you want your identity to be public for this peer review?**  For information about this choice, including consent withdrawal, please see our Privacy Policy.

Reviewer #1: **Yes:** Jerel Adam Fields

Reviewer #2: No

**********

[NOTE: If reviewer comments were submitted as an attachment file, they will be attached to this email and accessible via the submission site. Please log into your account, locate the manuscript record, and check for the action link "View Attachments". If this link does not appear, there are no attachment files.]

To ensure your figures meet our technical requirements, please review our figure guidelines: https://journals.plos.org/plosone/s/figures

You may also use PLOS’s free figure tool, NAAS, to help you prepare publication quality figures: https://journals.plos.org/plosone/s/figures#loc-tools-for-figure-preparation.

NAAS will assess whether your figures meet our technical requirements by comparing each figure against our figure specifications.

# Author comment: Author response to Decision Letter 1

**DOI:** 10.1371/journal.pone.0353267.r003

2 Jun 2026

Response to Reviewers

Submission title: Chronic CBD treatment differentially modulates neurobehavioral outcomes and endocannabinoid signaling in an aged HIV-1 Tat transgenic mouse model

We sincerely appreciate the effort in reviewing the manuscript and greatly appreciate all the reviewers’ suggestions and comments, which have significantly improved the quality of the manuscript. In revising the manuscript, we also noticed and corrected several additional minor errors. All our responses to the reviewers are noted in blue, italicized text below. Changes made in the text of the revised manuscript are highlighted in orange (non-italicized).

Reviewer #1: The study addresses a clinically relevant and understudied question—the effects of prolonged CBD exposure on neuroHIV in an aging context and represents a meaningful extension of the group’s prior work on acute CBD exposure in this model. The inclusion of sex as a biological variable across behavioral, lipidomic, and proteomic endpoints strengthens the translational relevance of the findings. However, several limitations should be addressed and/or discussed.

1. While all very important factors, the sample size is small to assess sex*genotype*treatment. It is impressive that the differences in readouts reached significance, but a post-hoc power analysis should be included with discussion of plans to affirm the findings in a larger cohort.

We thank the reviewer for this constructive point. We agree that a complex three-way interaction (sex x genotype x treatment) requires careful power considerations. As requested, we have conducted a post-hoc power analysis using G*Power (see image below) for all three-way interactions, which are now detailed in Supplemental Tables S1, S2, and S3.

Our analysis revealed that the majority of the readouts achieved a power >80%. However, we acknowledge that certain measures, including AEA and AA in the amygdala and GPR55 expression in the brainstem, fell below the 80% threshold despite reaching statistical significance. We recognize that these specific findings, while significant, should be interpreted with caution. Accordingly, we have updated our Materials and Methods to reflect the power analysis protocol and have added a statement to the Discussion section acknowledging the exploratory nature of these lower-powered interactions and the necessity for future validation in a larger cohort.

We have included the following statement in the Statistical analysis part of the Materials and Material and methods

“To assess the statistical reliability of the reported three-way interactions (sex x genotype x treatment), a post-hoc power analysis was conducted using G*Power 3.1. Achieved power for these interactions is reported in Supplemental Tables S1–S3.”

Additionally, the following statement is now added to the discussion.

“Sixth, although the study was sufficiently powered to detect major effects, some regional molecular shifts achieved significance with power below 80%. Future investigations with expanded sample sizes should be used to further confirm the strength of these specific three-way interactions.”

2. There is much discussion about the potential protective effects of CBD on the endocannabinoid system (ECS) and brain function, but there is little acknowledgement of the literature on state of the ECS in the brain (and systemic) in people with HIV. Considering the study is on the effects of CBD in a tat mouse, this seems pertinent to the intro and discussion.

We appreciate this suggestion and agree that incorporating clinical data on the eCB system in PLWH strengthens the manuscript’s rationale. We have added these pertinent details to the Introduction to better frame our study within the context of human pathology.

“Clinical evidence indicates a severe disruption in eCB tone, marked by significantly lower levels of AEA (PMID: 39156649) and OEA (PMID: 37093248) potentially driven by enhanced FAAH-mediated degradation in addition to altered 2-AG dynamics (PMID: 39156649). Furthermore, post-mortem tissue from individuals with HIV encephalitis (HIVE) and HAND on cART reveals a marked upregulation of CB1R and CB2R receptors in microglia, macrophages, and astrocytes (PMID: 21450051, PMID: 34578323), as well as a pathological shift in neuronal CB1R localization from a punctate distribution to the soma that correlates with cognitive decline (PMID: 34578323). This altered pathological landscape underscores the necessity of targeting the eCB system.”

3. The data availability section appears to be missing important information... XXX

Thank you for bringing this to our attention. All raw data generated in this manuscript is now deposited on the UNC Dataverse and will be published by the resubmission of this manuscript and can be found as Yadav-Samudrala, Barkha. 2026. “Chronic CBD Treatment Differentially Modulates Neurobehavioral Outcomes and Endocannabinoid Signaling in an Aged HIV-1 Tat Transgenic Mouse Model.” UNC Dataverse. https://doi.org/10.15139/S3/WGHAYI. Following information has been added to the “Data availability” section:

“All raw data and western blot files generated in this study can be found at the UNC Dataverse: Yadav-Samudrala, Barkha. 2026. “Chronic CBD Treatment Differentially Modulates Neurobehavioral Outcomes and Endocannabinoid Signaling in an Aged HIV-1 Tat Transgenic Mouse Model.” UNC Dataverse. https://doi.org/10.15139/S3/WGHAYI.”

4. Memory was tested in the mice and some lipids were measured in the hippocampus, but no western blots of the hippocampus. This should be added or discussed.

We thank the reviewer for this suggestion. We have now performed Western blot analysis on hippocampal tissue for CB1R, CB2R, GPR55, FAAH, and MAGL to complement our lipidomic and behavioral data. These results have been added to the Supplemental Information (S3_Table, S2_Fig, S3_Fig). We have also added raw unedited western blot images (S5_Fig).

5. While not entirely necessary, it is surprising that no measures of neuroinflammation were included, ie GFAP, IBA1, TNFa.

We appreciate the reviewer’s perspective on the importance of neuroinflammatory markers such as GFAP, IBA1, and TNF-α. While we agree these are critical components of HAND pathology, the primary objective of this study was to provide a high-resolution characterization of the eCB system and behavioral alterations following chronic CBD exposure. Due to the technical requirements of quantifying eCB ligands via mass spectrometry and the comprehensive profiling of five distinct proteins (CB1R, CB2R, GPR55, FAAH, and MAGL) via Western blot, the available tissue was fully utilized for these specific measurements. While our laboratory has an established record of assessing neuroinflammatory cytokines (e.g., IFN-γ, IL-1β) in other models of HIV such as HIV-1 Tg26, including our recent work in HIV-1 Tg26 mice (PMID: 41654202), we elected to maintain a strict focus on the eCB system in this manuscript to ensure a cohesive and deep mechanistic analysis. We have now acknowledged the exclusion of these markers as a limitation in the revised Discussion.

“Fifth, it is important to note that the current study focused exclusively on behavioral outcomes and the molecular profiling of the eCB system. Therefore, future studies integrating high-throughput eCB ligand analysis with localized cytokine profiling will be essential to fully elucidate the reciprocal relationship between CBD-induced eCB modulation and the attenuation of neuroinflammation in the Tat model.”

Reviewer #2: PONE-D-26-10019: Peer-Review Comments

This study examines the effects of chronic cannabidiol (CBD) treatment on aged HIV-1 Tat transgenic mice, investigating behavioral outcomes and changes in endocannabinoid system. The authors employed a 12-week CBD treatment protocol (3 mg/kg, s.c.) in 15-18 month-old mice, assessing cognition, anxiety-like behavior, nociception, and endocannabinoid signaling across multiple CNS regions. Key findings include gender-specific improvements in recognition memory (particularly in females), significantly altered thermal nociception, and complex region-dependent restructuring of the endocannabinoid system.

The work is supported by rigorous experimental design, clinically relevant aged model, multi-modal assessments across multiple brain regions, and inclusion of sex as a biological variable. The study’s longitudinal approach with multi-modal assessments (behavioral, biochemical) across four CNS regions (prefrontal cortex, amygdala, brainstem, spinal cord) provides valuable temporal and mechanistic insights. The inclusion of both sexes as a biological variable is particularly commendable given known sex differences in cannabinoid responses. Using aged mice addresses a critical gap in the literature, as most prior studies focus on young adults. This is highly relevant given the aging population of people living with HIV. The endocannabinoid system mapping is thorough. The combination of mass spectrometry for endocannabinoid ligand quantification and Western blotting for protein expression provides robust molecular characterization. The authors acknowledge and report three-way interactions (sex × genotype × treatment), demonstrating statistical sophistication and avoiding oversimplification of complex biological phenomena. Authors also acknowledge important limitations, for example, authors acknowledge that bulk tissue analysis cannot distinguish neuronal vs. glial contributions, which is critical given that Tat expression is astrocyte-driven in this model.

Specific Comments:

1. The authors report CBD-induced hyperthermia as a "critical physiological finding" (Page 31) yet continue to interpret/ discuss hot plate results as hyperalgesia. If baseline body temperature is elevated, isn’t the thermal gradient between body and hot plate is reduced? mechanically decreasing latency regardless of true nociceptive sensitivity? While the authors acknowledge this confounding issue ("explaining the paradoxical reduction in hot-plate latencies"), authors may need to adjust their conclusions accordingly, and revise the entire supra-spinal nociception narrative or, at minimum, explicitly state the limitations in discussion with regards to the extent of reliability of thermal nociception assessed in this context. Focusing solely on the thermoregulatory mechanisms and removing interpretive claims about hyperalgesia should be considered.

We sincerely appreciate the reviewer’s point regarding the physical mechanics of a thermal gradient; our empirical data demonstrates that the core temperature variance between groups was far too small to introduce a physical artifact.

In our study, the mean baseline body temperature for the chronic CBD group was 36.20°C, compared to 36.11°C in the vehicle control group. This represents an absolute physiological difference of only 0.09°C. Given that our hot-plate apparatus was maintained at a constant 55°C, this tiny baseline shift alters the physical thermal gradient (ΔT) between the animal’s paw and the testing surface by less than 0.5%.

Calculation:

• Hot plate was set to 55°C:

o Vehicle Gradient: 55 − 36.11 = 18.89°C

o CBD Gradient: 55 − 36.20 = 18.80°C

o The physical difference in the gradient = 0.09°C (0.47%)

Mathematically, less than 0.5% physical variance in a thermal gradient cannot possibly account for a statistically significant change in behavioral latency. Instead, our results revealed a robust, statistically significant reduction in hot-plate latency following CBD treatment (p<0.05). Because the magnitude of the behavioral response vastly exceeds this 0.09°C physical baseline shift, the data strongly support a true alteration in supraspinal nociceptive sensitivity rather than a thermoregulatory artifact.

We have now added the following statement to the discussion:

“It is important to note that while chronic CBD induced a statistically significant elevation in body temperature, the difference between vehicle- and CBD-treated groups was only 0.09 °C; this negligible (<0.5%) shift in the physical thermal gradient between the paw and the 55 °C testing surface cannot mathematically account for the robust reduction in hot-plate latencies, confirming a true alteration in supraspinal nociceptive sensitivity.”

2. The "psychostimulant-like effect" (Page 32) attributed to Tat-mediated dopaminergic dysregulation appears speculative without dopamine measurements. This aspect can be clearly stated. Clearly mentioning these as hypotheses based on preliminary observations requiring future testing is recommended.

We thank the reviewer for pointing out the speculative nature of attributing the observed hyperactivity to a 'psychostimulant-like effect' without direct dopamine measurements. We agree that while this is a well-supported mechanism in the existing Tat literature, it remains a hypothesis within the scope of our current dataset.

Following your recommendation, we have revised this section of the Discussion to explicitly state that this mechanistic explanation is a hypothesis based on our behavioral observations. We have tempered the language to ensure it reads as a potential pathway requiring future neurochemical validation rather than a definitive conclusion

“We hypothesize that this hyperactivity may be driven by Tat-mediated dysregulation of the dopaminergic system, specifically by interfering with dopamine transporters to increase synaptic dopamine levels, yielding a psychostimulant-like effect.”

“However, because direct dopamine kinetics were not quantified in the current study, this mechanism remains speculative and requires future empirical testing.”

3. All assessments occurred at week 16-17, at least 72 hours after the final injection, if I am correct. No information is provided about CBD half-life or acute/chronic pharmaco-physiological levels, making it unclear whether observed effects reflect drug presence or long-term neuroplastic changes (or both). I recommend discussing this briefly.

We thank the reviewer for highlighting this important pharmacological distinction. The reviewer is correct that behavioral and biochemical assessments were conducted 16–17 weeks into the protocol, approximately 72 hours following the final subcutaneous injection. Because CBD is highly lipophilic, chronic administration over 12 weeks likely results in tissue accumulation and a prolonged elimination half-life, creating a 'depot effect' in adipose and brain tissue. Therefore, we agree that the observed phenotypes and endocannabinoid alterations at the 72-hour mark likely reflect a combination of sustained steady-state drug presence and downstream, long-term neuroplastic adaptations.

Following your recommendation, we have added a brief discussion of CBD’s pharmacokinetics in senescent models to the limitations section, explicitly clarifying this timeline for the reader.

“First, it is important to contextualize the temporal relationship between our dosing regimen and behavioral testing. All assessment were conducted approximately 72 hours after the final chronic CBD treatment. Given the highly lipophilic nature of CBD, prolonged 12-week treatment should cause significant accumulation in lipid-rich compartments, including adipose tissue and the CNS, which can substantially extend the drug’s elimination kinetics via redistribution from tissue depots [170, 171]. While the acute plasma half-life of CBD in mice is relatively brief, prolonged chronic dosing regimens yield highly persistent, measurable concentrations of the drug directly in brain tissue [172]. Consequently, it remains unclear whether the reported behavioral and biochemical outcomes reflect the continuous, steady-state presence of residual CBD, permanent long-term neuroplastic adaptations within the eCB system, or a combination of both. Future studies incorporating explicit plasma and brain tissue clearance kinetics at multiple post-dosing intervals will be necessary to fully dissociate the acute physiological levels of CBD from enduring structural neuroplasticity.”

4. Tables 1-3 containing Tukey post-hoc comparisons should be moved to supplemental material. This improves readability while maintaining statistical rigor. Readers can grasp key pa

Attachment Submitted filename: Chronic CBD_Response to Reviewers.docx

# Peer review: Decision Letter 1

**DOI:** 10.1371/journal.pone.0353267.r004

**Authors:** Vinod K. Yaragudri

22 Jun 2026

Chronic CBD treatment differentially modulates neurobehavioral outcomes and endocannabinoid signaling in an aged HIV-1 Tat transgenic mouse model

PONE-D-26-10019R1

Dear Dr. Yadav-Samudrala,

We’re pleased to inform you that your manuscript has been judged scientifically suitable for publication and will be formally accepted for publication once it meets all outstanding technical requirements.

Within one week, you’ll receive an e-mail detailing the required amendments. When these have been addressed, you’ll receive a formal acceptance letter and your manuscript will be scheduled for publication.

An invoice will be generated when your article is formally accepted. Please note, if your institution has a publishing partnership with PLOS and your article meets the relevant criteria, all or part of your publication costs will be covered. Please make sure your user information is up-to-date by logging into Editorial Manager at Editorial Manager® and clicking the ‘Update My Information' link at the top of the page. For questions related to billing, please contact billing support.

If your institution or institutions have a press office, please notify them about your upcoming paper to help maximize its impact. If they’ll be preparing press materials, please inform our press team as soon as possible -- no later than 48 hours after receiving the formal acceptance. Your manuscript will remain under strict press embargo until 2 pm Eastern Time on the date of publication. For more information, please contact onepress@plos.org.

Kind regards,

Vinod K. Yaragudri, M.Sc., Ph.D

Academic Editor

PLOS One

Additional Editor Comments (optional):

Reviewers' comments:

Reviewer’s Responses to Questions

**Comments to the Author**

1. If the authors have adequately addressed your comments raised in a previous round of review and you feel that this manuscript is now acceptable for publication, you may indicate that here to bypass the “Comments to the Author” section, enter your conflict of interest statement in the “Confidential to Editor” section, and submit your "Accept" recommendation.

Reviewer #1: All comments have been addressed

Reviewer #2: All comments have been addressed

**********

2. Is the manuscript technically sound, and do the data support the conclusions?

The manuscript must describe a technically sound piece of scientific research with data that supports the conclusions. Experiments must have been conducted rigorously, with appropriate controls, replication, and sample sizes. The conclusions must be drawn appropriately based on the data presented.

Reviewer #1: Yes

Reviewer #2: Yes

**********

3. Has the statistical analysis been performed appropriately and rigorously?

Reviewer #1: Yes

Reviewer #2: Yes

**********

4. Have the authors made all data underlying the findings in their manuscript fully available?

The PLOS Data policy requires authors to make all data underlying the findings described in their manuscript fully available without restriction, with rare exception (please refer to the Data Availability Statement in the manuscript PDF file). The data should be provided as part of the manuscript or its supporting information, or deposited to a public repository. For example, in addition to summary statistics, the data points behind means, medians and variance measures should be available. If there are restrictions on publicly sharing data—e.g. participant privacy or use of data from a third party—those must be specified.

Reviewer #1: Yes

Reviewer #2: Yes

**********

5. Is the manuscript presented in an intelligible fashion and written in standard English?

PLOS ONE does not copyedit accepted manuscripts, so the language in submitted articles must be clear, correct, and unambiguous. Any typographical or grammatical errors should be corrected at revision, so please note any specific errors here.

Reviewer #1: Yes

Reviewer #2: Yes

**********

6. Review Comments to the Author

Please use the space provided to explain your answers to the questions above. You may also include additional comments for the author, including concerns about dual publication, research ethics, or publication ethics. (Please upload your review as an attachment if it exceeds 20,000 characters)

Reviewer #1: The authors did a nice job responding to critiques. The major and minor concerns were addressed and the manuscript is much improved.

Reviewer #2: Revision is adequate. Authors have made a sincere effort and have satisfactorily addressed the reviewers' comments.

**********

7. PLOS authors have the option to publish the peer review history of their article (what does this mean?). If published, this will include your full peer review and any attached files.

If you choose “no”, your identity will remain anonymous but your review may still be made public.

**Do you want your identity to be public for this peer review?**  For information about this choice, including consent withdrawal, please see our Privacy Policy.

Reviewer #1: **Yes:** Jerel Adam Fields

Reviewer #2: No

**********

# Editor report: Acceptance letter

**DOI:** 10.1371/journal.pone.0353267.r005

**Authors:** Vinod K. Yaragudri

PONE-D-26-10019R1

PLOS One

Dear Dr. Yadav-Samudrala,

I'm pleased to inform you that your manuscript has been deemed suitable for publication in PLOS One. Congratulations! Your manuscript is now being handed over to our production team.

At this stage, our production department will prepare your paper for publication. This includes ensuring the following:

* All references, tables, and figures are properly cited

* All relevant supporting information is included in the manuscript submission,

* There are no issues that prevent the paper from being properly typeset

You will receive further instructions from the production team, including instructions on how to review your proof when it is ready. Please keep in mind that we are working through a large volume of accepted articles, so please give us a few days to review your paper and let you know the next and final steps.

Lastly, if your institution or institutions have a press office, please let them know about your upcoming paper now to help maximize its impact. If they'll be preparing press materials, please inform our press team within the next 48 hours. Your manuscript will remain under strict press embargo until 2 pm Eastern Time on the date of publication. For more information, please contact onepress@plos.org.

You will receive an invoice from PLOS for your publication fee after your manuscript has reached the completed accept phase. If you receive an email requesting payment before acceptance or for any other service, this may be a phishing scheme. Learn how to identify phishing emails and protect your accounts at https://explore.plos.org/phishing.

If we can help with anything else, please email us at customercare@plos.org.

Thank you for submitting your work to PLOS ONE and supporting open access.

Kind regards,

PLOS ONE Editorial Office Staff

on behalf of

Dr. Vinod K. Yaragudri

Academic Editor

PLOS One
